# Supplementary material for: Quantifying ecosystem states and state transitions of the Upper Mississippi River System using topological data analysis
Source: PLoS Comput Biol. 2023 Jun 7;19(6):e1011147. doi: 10.1371/journal.pcbi.1011147 (PMC10246787; doi:10.1371/journal.pcbi.1011147)

**Title: Quantifying ecosystem states and state transitions of the Upper Mississippi River System using topological data analysis**

## SUPPORTING INFORMATION

**S1 Fig**. Graphical output from topological data analysis (TDA Mapper) showing a strong seasonal component to the water quality data for the Upper Mississippi River System, USA. Data inputs included 8 water quality variables collected four times each year during 1993–2020 at a total of 69,307 sampling sites. The graph is composed of nodes (circles), which cluster sampling sites along the river based on water quality similarities, and the edges (lines) show statistical connections among nodes. Different areas of the TDA structure are highlighted depending on season, indicating the eight water quality variables are affected by season.


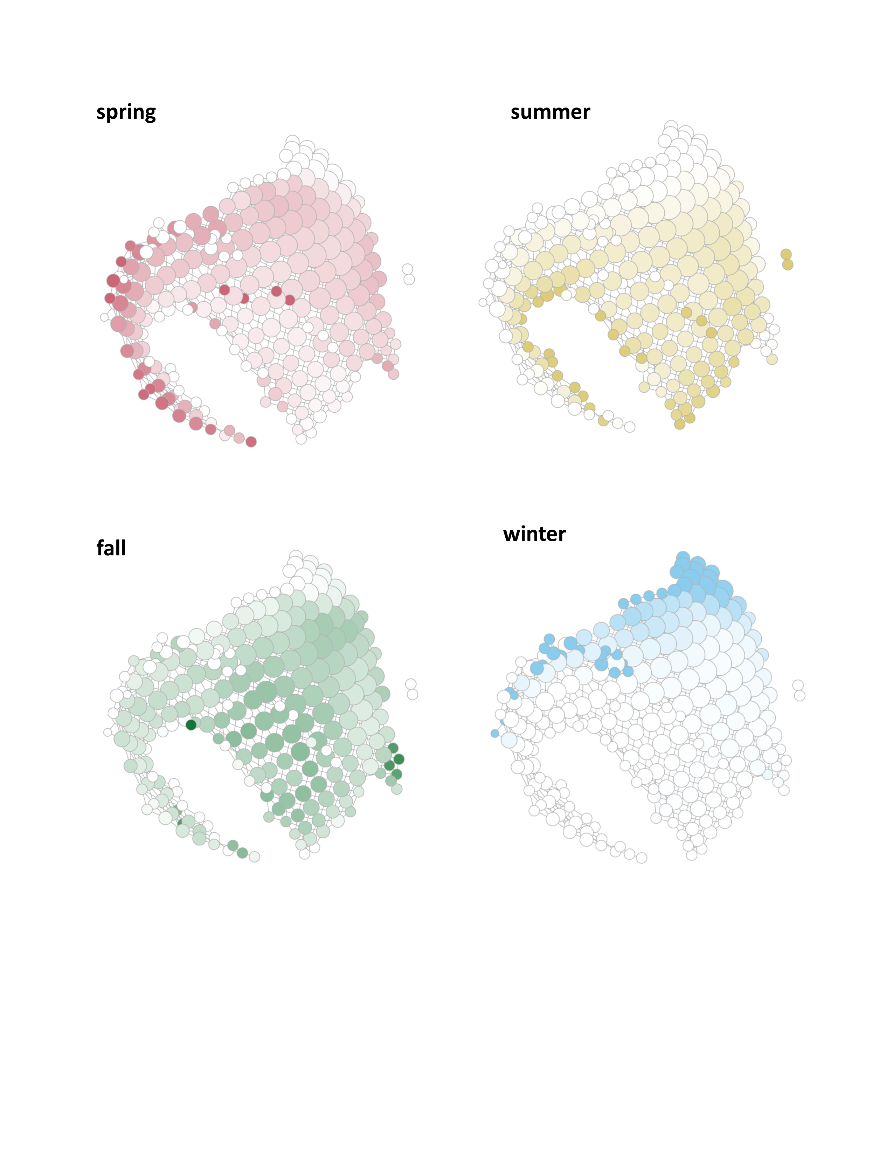

Supplement: S1 Fig — Data inputs included 8 water quality variables collected four times each year during 1993–2020 at a total of 69,307 sampling sites. The graph is composed of nodes (circles), which cluster sampling sites along the river based on water quality similarities, and the edges (lines) show statistical connections among nodes. We highlighted in color the seasons in which sites were sampled. Seasonality can be connected to ecosystem states by referencing Fig 1 positions; for example, winter is typically characterized by State 1. (DOCX) [file pcbi.1011147.s001.docx]
