# Supplementary material for: Quantifying ecosystem states and state transitions of the Upper Mississippi River System using topological data analysis
Source: PLoS Comput Biol. 2023 Jun 7;19(6):e1011147. doi: 10.1371/journal.pcbi.1011147 (PMC10246787; doi:10.1371/journal.pcbi.1011147)
Supplement: Attachment — Submitted filename: PCOMPBIOL-D-22-01462_rev_v2_responses to reviewer3.pdf [file pcbi.1011147.s004.pdf]

PLOS Computational Biology

Quantifying ecosystem states and state transitions of the Upper Mississippi River using topological data analysis

--Manuscript Draft--

**Manuscript Number:**	PCOMPBIOL-D-22-01462
**Full Title:**	Quantifying ecosystem states and state transitions of the Upper Mississippi River using topological data analysis
**Short Title:**	quantifying riverine states via topology
**Article Type:**	Research Article
**Keywords:**	ecological state; floodplain river; regime shift; state change; topology; topological state; water quality; vulnerability
**Abstract:**	Aquatic systems worldwide can exist in multiple ecosystem states (i.e., a recurring collection of biological and chemical attributes), and effectively characterizing multidimensional states will aid protection of desirable states and guide restorations. The Upper Mississippi River System is a large floodplain river spanning 2200 km with a 30-year water quality monitoring data to depict ecosystem states and guide river restoration. We coupled this highly dimensional dataset with multiple topological data analysis (TDA) techniques to classify ecosystem states, identify state variables, and detect state transitions over 30 years. Across the entire river system, TDA identified five ecosystem states. State 1 was characterized by exceptionally clear, clean, and cold water (i.e., a 'clear-water state'); State 2 had the greatest range of environmental conditions; and States 3, 4, and 5 had very high concentrations of suspended solids (i.e., 'turbid-states'). The TDA mapped clear patterns of the ecosystem states across several riverine navigation pools, seasons, and habitat types that furthered ecological understanding. State variables were identified as suspended solids, chlorophyll a, and total phosphorus. Some pools underwent seasonal state transitions each year, entering State 1 in winter and State 2 in other seasons. One pool transitioned sporadically several times between States 2 and 3. The TDA was a novel technique that showed the river was characterized by predictable ecosystem states and a few state variables, and pools experienced state transitions based on seasonal or episodic events. These results can inform decision making and guide actions for regulatory and restoration agencies by assessing the status and trends of this important river and providing quantitative targets for state variables if management aims to purposely shift the turbid-state to more desirable conditions like States 1 and 2. The TDA correlation function may serve as a new tool for predicting the vulnerability to undesirable state transitions. These state concepts and TDA methodologies can be transferred to any ecosystem to help classify states and possibly predict vulnerability and causes of state transitions.
**Additional Information:**	
**Question**	**Response**
**Government Employee**<br><br>Are you or any of the contributing authors an employee of the United States government?<br><br>Manuscripts authored by one or more US Government employees are not copyrighted, but are licensed under a CC0 Public Domain Dedication, which allows unlimited distribution and reuse of the	Yes - One or more authors are employees of the U.S. government.

Powered by Editorial Manager® and ProduXion Manager® from Aries Systems Corporation

article for any lawful purpose. This is a legal requirement for US Government employees.<br><br>This will be typeset if the manuscript is accepted for publication.	
**Financial Disclosure**<br><br>Enter a financial disclosure statement that describes the sources of funding for the work included in this submission and the role the funder(s) played. This includes grants and any commercial funding of the work or authors.<br><br>This statement will be typeset if the manuscript is accepted for publication.<br><br>Review the submission guidelines and the instructions link below for detailed requirements and guidance.	The data collection, data analysis, manuscript preparations, and decision to publish were funded as part of the U.S. Army Corps of Engineers' Upper Mississippi River Restoration Program (https://umesc.usgs.gov/ltrm-home.html). The National Science Foundation's "Research Experience for Undergraduates" (REU) Program Award Number:1559663 funded authors CM, AS, AL, KD, FM for data analysis and manuscript preparation (https://beta.nsf.gov/funding/opportunities/research-experiences-undergraduates-reu).
**Competing Interests**<br><br>On behalf of all authors, disclose any competing interests that could be perceived to bias this work.<br><br>This statement will be typeset if the manuscript is accepted for publication.<br><br>Review the instructions link below and PLOS Computational Biology's competing interests policy to determine what information must be disclosed at submission.	none
**Data and Code Availability**<br><br>From the time of publication, Authors are required to make fully available and without restriction all data and computational code underlying their findings. Please see our PLOS Data Policy page for detailed policy information, and our Code Sharing page for specific	All data were publicly accessed on 1 June, 2021 through the Upper Mississippi River Restoration Program's Long-term Resource Monitoring (https://umesc.usgs.gov/ltrm-home.html). The data used in this analysis is provided in the attached Supporting Information file, 'Mississippi River TDA data set.csv.' Our final datasets and analysis scripts will be archived in GitLab (DOI: XXXXXXXX) following peer review through the U.S. Geological Survey (in progress) and prior to publication.

Powered by Editorial Manager® and ProduXion Manager® from Aries Systems Corporation

information on code sharing.<br><br>A **Data Availability Statement**, detailing where the data (and code, if applicable) can be accessed, is required at first submission. Insert your Data Availability Statement in the box below. The statement you provide **will be published in the article**, if accepted.<br><br>Please see the Data Reporting section of our submission guidelines for instructions on what you need to include in your Data Availability Statement.<br><br>If the data and code are all contained in your submission files, please state: *All relevant data are within the manuscript and its Supporting Information files.*<br><br>**PLOS allows rare exemptions to address legal and ethical concerns.** If you have legal or ethical restrictions, please detail these in your Data Availability Statement below for the Journal team to consider.	

Powered by Editorial Manager® and ProduXion Manager® from Aries Systems Corporation

Manuscript and figures

Click here to access/download;Manuscript;Larson TDA river PLOS 03Oct2022 review.docx

Title: Quantifying ecosystem states and state transitions of the Upper Mississippi River using topological data analysis

Authors: Danelle M. Larson[1‡],Wako Bungula[2], Casey McKean[3], Alaina Stockdill[3], Amber Lee[3], Frederick Miller[3], Killian Davis[3]

Affiliations:

[1] U.S. Geological Survey, 2630 Fanta Reed Road, La Crosse, WI 54603; dmlarson@usgs.gov; no fax; ORCID: 0000-0001-6349-6267; ‡ Corresponding Author: dmlarson@usgs.gov

[2] University of Wisconsin La-Crosse, Department of Mathematics and Statistics, 1027 Cowley Hall, La Crosse, WI 54603

[3] University of Wisconsin La-Crosse, Research Experience for Undergraduates Program, 1027 Cowley Hall, La Crosse, WI 54603

Corresponding author: Danelle M. Larson, dmlarson@usgs.gov

Running title: quantifying riverine states via topology

ABSTRACT

Aquatic systems worldwide can exist in multiple ecosystem states (i.e., a recurring collection of biological and chemical attributes), and effectively characterizing multidimensional states will aid protection of desirable states and guide restorations. The Upper Mississippi River System is a large floodplain river spanning 2200 km with a 30-year water quality monitoring data to depict ecosystem states and guide river restoration. We coupled this highly dimensional dataset with multiple topological data analysis (TDA) techniques to classify ecosystem states, identify state variables, and detect state transitions over 30 years. Across the entire river system, TDA identified five ecosystem states. State 1 was characterized by exceptionally clear, clean, and cold water (i.e., a 'clear-water state'); State 2 had the greatest range of environmental conditions; and States 3, 4, and 5 had very high concentrations of suspended solids (i.e., 'turbid-states'). The TDA mapped clear patterns of the ecosystem states across several riverine navigation pools, seasons, and habitat types that furthered ecological understanding. State variables were identified as suspended solids, chlorophyll *a*, and total phosphorus. Some pools underwent seasonal state transitions each year, entering State 1 in winter and State 2 in other seasons. One pool transitioned sporadically several times between States 2 and 3. The TDA was a novel technique that showed the river was characterized by predictable ecosystem states and a few state variables, and pools experienced state transitions based on seasonal or episodic events. These results can inform decision making and guide actions for regulatory and restoration agencies by assessing the status and trends of this important river and providing quantitative targets for state variables if management aims to purposely shift the turbid-state to more desirable conditions like States 1 and 2. The TDA correlation function may serve as a new tool for predicting the vulnerability to undesirable state transitions. These state concepts and TDA methodologies can be transferred to any ecosystem to help classify states and possibly predict vulnerability and causes of state transitions.

Keywords: ecological state, floodplain river, regime shift, state change, topology, topological state, water quality, vulnerability

AUTHOR SUMMARY

The Mississippi River in the USA is a large, complex ecosystem that had many changes to water quality since the 1990s. We used a new set of mathematical tools called 'topological data analysis' to help identify common water quality conditions or 'states' of this important river. We also explored when the states have changed over the past 25 years and vulnerability to change. The tools identified five common water quality states, distinguished by either by clear waters or muddy waters. The state changes were not abrupt but rather had improved or declined gradually over many years, depending on the river reach. River reaches named Pool 4 and Pool 8 had become clearer over time. Pool 13 became muddier over time. Three other river reaches were consistently muddy. Our study is a good example of how these topological data analysis tools may help others managing ecosystems by improving communications about what states exist and which are desirable (for example, the clear water state is desirable). Using these tools with long-term data can also alert ecosystem managers to the ongoing, complex changes and vulnerability to undesirable changes so they can intervene with restoration.

Author contributions:

DL and WB conceptualized study, secured funding, curated data, and shared data; all authors prepared data and analyzed data; AS, AL, and CM wrote final analysis codes and created figures; DL and WB wrote first paper draft; all authors edited paper drafts and approved of journal submission.

Data availability statement:

All data were publicly accessed on 1 June, 2021 through the Upper Mississippi River Restoration Program's *Long-term Resource Monitoring* (https://umesc.usgs.gov/ltrm-home.html). The data used in this analysis is provided in the attached Supporting Information file, 'Mississippi River TDA data set.csv.'

Our final datasets and analysis scripts will be archived in GitLab (DOI: XXXXXXXX) following peer review through the U.S. Geological Survey (in progress) and prior to publication.

INTRODUCTION

Many ecosystems worldwide are undergoing severe and rapid changes and managing ecosystem states and states transitions are a time-sensitive and difficult challenge (1). Large floodplain rivers are especially degraded and continue to experience major changes to hydrology and water quality (2–5). Monitoring ecosystem states and states transitions with long term data and advanced analytical tools is critical for protecting and restoring ecosystems that humanity values or depends on (6).

Ecosystem states (i.e., a recurring collection of biological and chemical attributes) are well defined in many types of ecological systems, but not for large rivers. Many biomes have at least two states defined by the lower trophic levels, such as the plant-dominated or phytoplankton-dominated states (7,8), marine kelp forest or deforestation (9), mesic grasslands or desertification (6), mesic grasslands or shrublands (10), and diverse coral reefs or macroalgal-dominated reefs (11). State characterization is always multidimensional, and typically has only a few state variables although sometimes is defined by complex biological communities (12). A few examples of state transitions include the restoration of aquatic vegetation and water quality in shallow lakes (8), altered disturbance regimes and subsequent restorations in tallgrass prairies (13,14), and successive state shifts and a novel state arising from multiple stressors in a deep lake (15).

Ecosystem states have been conceptualized (16,17) but not well tested in rivers until recently (18,19). The global scientific and management community calls for applying an ecosystem states framework to rivers, while acknowledging most rivers do not have sufficient data to properly evaluate states (20,21). When big data are available, identifying the states and their constraints is key for ecological restoration planning, post-project monitoring, and adaptive management (22).

State concepts are complex and multidimensional, and therefore requires substantial data, advanced and novel analysis techniques for disentangling patterns, and expertise for its ecological interpretations. More analytical tools for 'big data' that can reasonably predict ecosystem states and

impending state transitions will help conserve, restore, and manage ecosystem resilience (6). Topological data analysis (TDA) provides a powerful suite of tools for disentangling patterns and relationships among multidimensional data and presenting data visually (23). Tools of TDA have effectively shown changes in the human gut following bacterial disturbance (24), predict wildfire severity (25), and assess vulnerability of residential homes to climatic changes (26). The use of TDA remains rare in ecology, although TDA may be an ideal tool for many ecological applications because of its multidimensional framework (27).

Objectives and Hypotheses:

We provide an analysis of ecosystem states and transitions using topological data analysis (TDA) tools to better characterize the Upper Mississippi River System in the USA, which is a significant ecosystem for humans and wildlife (28). We hypothesized that this river would be in at least two states (17,21): a 'clear-water state' characterized by clean and clear water, and a 'turbid-state' characterized by high turbidity due to high suspended solids and chlorophyll *a* (Hypothesis 1). We expected at least three state variables based on nutrients and turbidity (Hypothesis 2) but included up to eight hypothesized state variables within TDA. We explored whether different riverine pools were in different states (Hypothesis 3) given their differing water quality conditions and restoration priorities (28,29). We expected TDA would detect an abrupt state transition around years 2008–2010 when aquatic vegetation rebounded (30) and water quality conditions improved (28) (Hypothesis 4a), and that TDA could also detect gradual water quality improvements based on the proportion of sites entering a 'clear-water state' (Hypothesis 4b).

RESULTS

How many ecosystem states were in the UMRS?

The TDA Mapper graph output has six connected components where the main component represented more than 99% of the data (Figure 1). Only <1% of the data points and were excluded from the main TDA structure as 'outliers' or 'noise' (Figure 1 dark violet nodes). The main component had a "body" and a "tail," suggesting there were at least two main ecosystem states as determined by topology (in support of Hypothesis 1).

Figure 1. Graphical output from topological data analysis (TDA Mapper) of the potential ecosystem states for the Upper Mississippi River System, USA. Data inputs included 8 water quality state variables collected four times each year from 1993–2020 at 69,307 sampling sites. TDA Mapper showed five topological states (color coded, which can be conceptualized as ecosystem states), and most sites were within State 2. The dark purple nodes not connected to the main structure (<1% of sites) were outliers and not classified within a state.

The topological states algorithm classified 76,669 sites from the UMRS over 27 consecutive years into five ecosystem states (Figure 1) by using 8 water quality variables. These five distinct states were refined and nested within the body and tail of the TDA structure; States 1 and 2 were nested within the body, and States 3–5 were nested within the tail. There were five, small transition nodes in the TDA structure, which lied between states 1 and 2. States 1, 2, 3, 4, 5 contained 20%, 85%, 2%, <1%, and <1% of data, respectively.

What were the state variables that defined and distinguished riverine ecosystem states?

State 2 contained most sites (Table 1), and the state variables differentiating State 2 from others included total phosphorus, chlorophyll *a*, and suspended solids (in support of Hypothesis 2). However, State 2 had the greatest variability and many statistical outliers for all state variables. State 1 may be

considered the 'clear-water state' because this state had the lowest total phosphorus, highest dissolved oxygen, lowest chlorophyll *a*, coldest water temperatures, slowest water velocity, and lowest suspended solids relative to the other four states (Figure 2). State 1 was differentiated from State 2 principally by its colder water temperatures and lower suspended solids (Figures 2f, 2h). State 2 typically had lower nutrients but higher chlorophyll *a* relative to States 3, 4, and 5.

Table 1. The distribution of sites in each upper Mississippi River pool (column 1: Pool name, field station city and state) that were classified into a topological/ecosystem state within years 1993–2019. **Table 1a** shows the proportion (%) of sites in each state according to river pool for comparisons of ecosystem states among the six pools. **Table 1b** shows the proportion (%) of sites classified within a pool to compare the distribution o[illegible]tes within each pool. Sites sometimes can exist in more than one node, and therefore the percentage of sites within a state may sum to greater than 100%.

TABLE 1a

River pool	State 1	State 2	State 3	State 4	State 5
**Pool 4 (Lake City, MN)**	31%	16%	0%	0%	0%
**Pool 8 (Onalaska, WI)**	35%	17%	<1%	0%	0%
**Pool 13 (Bellevue, IA)**	26%	18%	<1%	0%	0%
**Pool 26 (Brighton, IL)**	5%	16%	8%	5%	6%
**Open River (Jackson, MO)**	<1%	17%	90%	95%	93%
**La Grange (Havana, IL)**	2%	16%	<1%	0%	<1%
**Total sites**	**100%**	**100%**	**100%**	**100%**	**100%**

TABLE 1b

River Pool	State 1	State 2	State 3	State 4	State 5
**Pool 4 (Lake City, MN)**	37	81	0	0	0
**Pool 8 (Onalaska, WI)**	39	78	<1	0	0
**Pool 13 (Bellevue, IA)**	27	82	<1	0	0
**Pool 26 (Brighton, IL)**	7	95	<1	<1	<1
**Open River (Jackson, MO)**	<1	87	9	3	1
**La Grange (Havana, IL)**	5	92	<1	0	<1

Figure 2. Graphical outputs from topological data analysis (TDA Mapper) showing ecological gradients of 8 water quality state variables in the Upper Mississippi River System, USA (*n*=69,307 sampling sites from 1993–2020). The color coding represents relative values for each variable; dark violet colors are relatively low values, whereas greens and yellows are relatively high values. The boxplots below each TDA Mapper graph showed the data distributions for each ecosystem state (that corresponds to the states in Figure 1). Boxplots are standardized by boxes (containing the 25th, 50th (median), and 75th percentiles), the whiskers (containing the 0th and 100th percentiles, excluding outliers), and the dots (statistical outliers). The x-axis on the boxplots represents the five states (States 1–5). The y-axis represents variable units (total nitrogen = mg L^{-1}; total phosphorus = mg L^{-1}; dissolved oxygen = mg L^{-1}; chlorophyll *a* = µg L^{-1}; water depth = cm; temperature = °C; velocity = m sec^{-1}; suspended solids = mg L^{-1}.

States 3, 4, 5 within the structure's tail resembled the 'turbid-state.' These three states were like each other for seven water quality variables (Figure 2a–2g) and differentiated from one another by the relative concentrations of suspended solids (Figure 2h). Compared to States 1 and 2, the States 3–5 had greater total phosphorus, lower dissolved oxygen, greater water depths and velocities, and orders of magnitude greater suspended solids. Total nitrogen was the only variable that didn't vary across the five state classifications (Figure 2a), so we determined total nitrogen was not a state variable.

Did pools exist in different ecosystem states?

The six study pools spanning 2200 km of river were classified into different ecosystem states (in support of Hypothesis 3). Evidence for the different states were shown by the highlighting in different locations on the main TDA structure (Figure 3) and the percentage of sites in each pool classified to a state (Table 1). Pool 4 (Minnesota), Pool 8 (Wisconsin), Pool 13 (Iowa), and La Grange Pool (Havana, Illinois; Illinois River) were within States 1 and 2 across the 27-year record. Therefore, Pools 4, 8, and 13 were principally characterized as having a broad range of ecological conditions that ~~sometimes~~ included the 'clear-water' state (27–39% sites within State 1). Pool 26 (Brighton, Illinois) existed in all five states but State 2 was dominate. Open River (Jackson, Missouri) was the principal ~~pool~~ within States 3-5. Open

River was rarely in State 1, mostly within state 2, and 13% of sites were within the turbid-states of States 3–5.

Figure 3. Topological data analysis (TDA) showed one primary data structure for 8 water quality state variables from six navigation pools on the Upper Mississippi River System (years 1993–2020), and the green colors highlighted where sampling sites within each pool grouped onto the primary TDA Mapper structure. Dark green nodes had relatively many sites within the node compared to the lighter green nodes containing fewer sites. Different positions on these TDA Mapper graphs were highlighted in green according to pool, indicating these pools have belong to different ecosystem states based on water quality. Pools can be classified into specific ecosystem states by referencing their positions within Figure 1; for example, the Pool 4 was within States 1 and 2 because the green highlights are on the left side of the TDA structure (panel a). As another example, Open River pool was principally within States 3, 4, and 5 because the green highlights are on the right side of the TDA structure and its tail (panel f). Pools can also be characterized by water quality state variables in Figure 2; for example, Open River pool is typically characterized by high velocity and suspended solids.

Did pools undergo state transitions or was there state stability over the past 30 years?

Pool 4 and Pool 8 shifted towards State 1 through time, albeit gradually. The temporal correlation function did not detect a dramatic ecological regime shift around years 2008–2010 in Pools 4 and 8 (refuting Hypothesis 4a), as indicated a lack of monotonic decrease in correlations between States 1 and 2 (Figure 4). However, Pool 4 and Pool 8 had increasing minimum correlation values through time for State 1, especially after year 2010. The increasing correlation values indicated State 1 became more prevalent over time and thus water quality conditions improved in those two pools (in support of Hypothesis 4b).

Figure 4. Temporal correlation function plots showed the transitions in ecosystem states within six pools of the Upper Mississippi River System from years 1993–2020. The correlation (y axes) is the proportion of sites within each state compared to the number of sites collected during that season (~125 sites per season per year per pool). Occasionally, sites were classified into multiple states, and therefore the correlation sum may exceed 1.0. If correlation lines from States 1–5 never crossed in the time series, that pool had state stability and had not experienced state changes. If correlation lines crossed temporarily, this indicated instable and transient dynamics due to seasonal effects or episodic events. If a state had a monotonic decrease in correlation and then correlation lines crossed, this could have indicated a long-

lasting state change or ecological ‘regime shift.’ Points that fall directly on the year were collected in winter, followed by spring, summer, and autumn samples. Data from 2003 was missing ~~due to logistical constraints for collection~~.

The six pools varied substantially in their ecosystem state stability and the frequency of state transitions, if any (Figure 4). Pool 26 and La Grange Pool were stable within State 2 and occasionally State 1 and did not undergo state transitions. Pools 4, 8 and 13 displayed cyclic dynamics between State 1 and State 2 that were recurring annually due to seasonal changes. State 1 dominated in winter of most years and State 2 dominated in the other three seasons. State 1 was uncommon but possible outside of winter, and State 1 was especially rare in summer (indicated by low correlation values during summers). State 1 was seasonally dynamic as indicated by cyclic correlation values that were greatest in winter, declined by spring, and then increased by the following autumn and winter.

Pool 13 showed different patterns in state transitions (Figure 4). The increased minimum correlation values over time in State 1 was less pronounced than in Pools 4 and 8, indicating less water quality improvements. Since 2015 in Pool 13, the expected pattern of transitioning to State 1 in winter had become less common and predictable compared to previous years. State 3 (a turbid-state with very high suspended solids) was first entered in 2017 and again in 2019 at some Pool 13 sites, indicating declining water quality conditions from increasing suspended solids.

Open River (Jackson, Missouri) transitioned between State 2 and State 3 four times sporadically over 27 years. ~~State 3 became predominate, which only lasted one or two seasons before returning to State 2.~~ The four transitions occurred across various seasons and years (specifically, spring-summer 1999; winter-spring 2010; winter 2014; autumn 2015).

DISCUSSION

This is one of the first studies to use powerful topology tools to quantify ecosystem states and state transitions in landscapes and riverscapes. Our topological analyses successfully revealed multiple

riverine states, the defining state variables, and differences among pools in the Upper Mississippi River System (in support of our Hypotheses 1–3 and visualized in Figures 2–4). The TDA detected short-term state changes based on seasonality and episodic events and provided evidence of gradual, long-term changes due to water quality improvements over three decades (Figure 4). We discuss how TDA may be used as a new technique for describing ecosystem states at multiple spatiotemporal scales, how the results will inform ecological restoration and management, and how the state concepts tested with TDA methods may be transferred to any ecosystem that exists in one or more states.

TDA as a novel technique for ecosystem states

Testing ecosystem state concepts with TDA were inspired by past research of the human-gut microbiome (24,31), and our study shows TDA can also effectively disentangle states from big data of large ecosystems. Our TDA results aligns well with previous studies of Upper Mississippi River System that examined broad ecological gradients among pools and water quality changes through time in some pools (28,32,33). The TDA results supported a hypothesis that the Upper Mississippi River System exists in two alternate regimes of clear-water and turbid-states as defined by state variables of water clarity due to suspended solids and chlorophyll *a* (17). However, State 2 had very high variability across all state variables to be of practical use unless comparing directly to States 3–5. Likewise, the main differentiation between States 3–5 were based solely on suspended solid concentrations, which may be better represented as an ecological gradient than firm state classification via TDA. Our results expand the existing knowledge by using a multivariate approach to classify nearly 70,000 sites into multiple ecosystem states, identify water quality state variables, and explore state transitions. The TDA trend analysis examined multivariate (state) transitions (Figure 4 and temporal correlation results) rather than assessments of single variables and their changes as in Houser (2022).

The TDA allowed exploration of whether state transitions were dramatic and long-standing (i.e., an abrupt regime shift) or whether transitions were temporary state changes (i.e., transient dynamics) (Hypothesis 4). The TDA detected frequent transient dynamics based on seasonality and episodic state changes (Figure 4) but did not detect abrupt state changes with sharp break points in time. Future TDA analyses could include data on submersed aquatic plant biomass or species composition (34) because aquatic plants are likely a state variable (17) that may reveal abrupt shifts around 2010 in support of our Hypothesis 4. Other TDA tools like 'persistence homology' can detect change points (35), and abrupt state transitions are hypothesized to occur in rivers with long retention times (36) or following ecological restoration (17). Alternatively, the river's ecology may be multidimensional and complex so that there was not a single point in time where major and abrupt changes happened, but rather changes were gradual over decades and most accurately portrayed with the temporal correlation function used herein (Figure 4). The lack of abrupt regime shifts in the Upper Mississippi River System would be different than is commonly observed within nearby shallow lake systems, which can transition to the alternative state within a few days and persist for years (7,8).

The TDA also allows extraction of information at finer spatiotemporal scales but was beyond the scope of this paper. For example, information from the data within the nodes can further describe how specific sites, seasons, riverine habitat strata, and years are classified within each state to further ecological understanding and prioritize ecological restorations. Sites classified within multiple states, transition nodes, or the TDA outliers could be further explored for unusual site characteristics and assessing site-level variability that affects ecosystem state.

Ecosystem states results can guide ecological management & restoration

Coupling ecosystem state theories with scientific models can provide urgently needed guidance for ecological restoration and management (37,38). Previously applied uses of state theory included

predicting pending state transitions (39–41), managing systems for resilience and preventing undesirable transitions (40), and managing state variables to restore or protect resilience of desirable ecosystem states (8,42). However, state classification and state transition models are rarely used in large monitoring programs because of modeling complexities and the inability of institutions to practically apply model outputs (6).

We used big data (70,000 sites and 8 variables) from long-term river monitoring (43), and these TDA results can guide structured decision making, adaptive management, and vulnerability assessments in the Upper Mississippi River System. This is the first study in this large watershed to classify ecosystem states, which is foundational for describing the system's ecological structures and functions. Having defined ecosystem states allows practitioners to communicate which states are desirable for preservation and which regimes are undesirable for mitigation, as initiated in (17,29,32). We identified the state variables (specifically total phosphorus, suspended solids, and chlorophyll *a*), which can guide decision making and actions for regulatory and restoration agencies by providing quantitative targets. Currently, specific regional water quality criteria are recommended for total phosphorus and chlorophyll *a*, but not suspended solids (44). There remains a lack of a specified targets for suspended solids concentrations although water clarity remains a common restoration goal (29), and our TDA results echo the importance of suspended solids a state variable.

The TDA's correlation function can be a novel tool for assessing state transitions and vulnerability as new, long-term data are gathered. This function effectively signaled state changes in the Upper Mississippi River System due to seasonality, episodic events, and gradual changes to water quality (Figure 4). In addition to assessing individual water quality variables through time and across ecological gradients (28), TDA can describe the status and trends of the holistic ecosystem due to natural changes and restoration impacts. The state transition modeling can continue to reveal gradual improvements to water quality and signal vulnerability to undesirable state transitions if States 3–5 become more

prominent (Figure 4). The correlation function gave an early warning signal of imminent state transitions in Pool 13. The TDA tool indicated Pool 13 may be vulnerable to transitioning to the undesirable turbid-state (State 3; Figure 4) because it has transitioned to State 3 repeatedly only in the last few years of a 30-year record and the high suspended solid concentrations characteristic of State 3 are undesirable in this system (29). The TDA could detect if more sites within Pool 13 continue to enter State 3 more frequently or persistently in the near future. Lastly, the states could become a categorical response variable in future models that aim to assess the environmental drivers and threshold responses, although researchers should carefully consider whether such information is better addressed with gradient analyses instead of state classification (45).

Transferability of TDA to other ecosystems

Overall, TDA was a novel technique that effectively addressed many hypotheses regarding the Upper Mississippi River System as characterized by predictable ecosystem states, a few state variables, and state transitions. The TDA may provide an early warning indication of vulnerability to undesirable ecosystem transitions. Testing ecosystem states in freshwater systems (beyond well-studied shallow lakes) is important, while being cognizant to the limitations and misuses of the theories (45). The TDA is advantageous in that model outputs can be derived for a variety of spatial scales (e.g., site-, habitat-, pool-, and systemic-levels) to match management objectives and a manager's geographical jurisdiction. Selection of state variables for TDA should be ecologically meaningful and allow managers to influence those variables to advance conservation (46). The state concepts and TDA methodologies can be transferred to any ecosystem to help classify states and predict their vulnerability to state transitions.

MATERIALS AND METHODS

Study Area, Ecosystem State Context, and Long-term Data

The upper Mississippi River flows ~2200 km across the USA and its 29 locks and dams have created ‘pools’ upriver of each impoundment. Lock and dams were installed in the late 1930’s. Aquatic plants were abundant and then were abruptly lost in the late 1980’s for reasons unknown. Aquatic plants gradually returned and has remained resilient since 2010 even under extended flood conditions from 2017-2019 (Larson et al. 2021). We studied six navigation pools (herein called ‘pools’ for brevity) named Pool 4, Pool 8, Pool 13, Pool 26, Pool La Grange (Illinois River) and Open River that span multiple environmental gradients like geomorphology, hydrology, and water quality. The study area was a high restoration priority and had long-term data on water quality.

We assessed ecosystem states based on water quality data at 69,307 sampling sites across the six pools (collected four times annually in each season from years 1993–2020), which is a 27-year consecutive record. The water quality data was collected by a long-term, standardized protocol whereby sites were selected by a stratified random sampling design and strata were habitat types like main channel borders, contiguous backwaters, impounded areas above the lock and dams, and lotic side channels (47). Using ecological literature and *a priori* hypotheses, we chose eight water quality variables thought to be important for defining ecosystem states and ensured the variables were not highly correlated (Pearson’s $r < 0.45$). The 8 variables included: total nitrogen = mg L^{-1}; total phosphorus = mg L^{-1}; dissolved oxygen = mg L^{-1}; chlorophyll a = µg L^{-1}; water depth = cm; temperature = °C; velocity = m sec^{-1}; suspended solids = mg L^{-1}.

Quantifying ecosystem states and the state variables

To quantify the ecosystem states, we performed four main tasks (Figure 5): 1.) data pre-processing, 2.) TDA Mapper algorithm, 3.) density-based state assignment, and 4.) identifying state

variables. To detect state transitions over 27 years, we performed a final task: 5.) Temporal Correlation Function.

Figure 5: Flow chart that summarized our topological data analysis (TDA) methods in sequential order (arrows and gray text) and outputted products (boxes with black text and images) for quantifying ecological states and state transitions of the Upper Mississippi River. The original- and TDA-ready datasets are available here (DOI: XXXX). In the “Undirected TDA Mapper” box, nodes and edges are connected to create a single TDA structure. In “Directed TDA Mapper” box, the example output describes two local maxima nodes defining two states: a red state containing two nodes and a blue state containing one node. The grey node is a transition node and does not belong to state. In the “change detection” box, we provide a hypothetical output of where the dominance of two states (red and blue dashed lines, which are the proportion of sites within those states) temporarily transition from the dominant red state to the blue state around years 13 to 17.

1. Data Pre-processing

The original water quality dataset (accessed 06/01/2021 from https://umesc.usgs.gov/data_library/water_quality/water1_query.shtml), had 204,345 rows (site data) and 133 columns (variables), and contained data from 1993 to 2020, except 2003. We filtered the data by stratified random sampling scheme, surface water, and 8 continuous variables. Negative values, which was <0.2% of the data, were set to the minimum value of that variable in the entire dataset, and quality flag (QF) codes of ‘0’, ‘A’, ‘8’, or ‘64’ were set to ‘NA.’ Duplicate site identifiers were combined with the arithmetic mean value replacing the existing column values. After we removed three unreasonable and extreme outliers (TN > 25 mg/L), the final data size was 69,307 rows by 8 columns. ~~The three variables with ~66% of the values per the study design were total nitrogen, total phosphorus, and water velocity.~~ To address missingness, we compared seven interpolation methods and utilized a random forests algorithm as the top preforming method. The other occasional missing values were imputed with the median value for each variable. ~~Two water clarity variables, turbidity and secchi disk~~

depth, were not used because they were highly correlated with suspended solids. We used robust scaling so each variable's differing measurement units were accounted for in the multivariate analysis.

2. The TDA Mapper Algorithm & Topological States Definition

The Topological Data Analysis (TDA) Mapper algorithm visualizes high dimensional data by incorporating dimension reduction techniques and clustering algorithms (48). The TDA Mapper algorithm takes in point cloud data and produces a 'simplicial complex', which is graph showing a TDA structure from a collection of nodes and edges (Figure 5). We used Keppler Mapper (a TDA Mapper) written in Python (van Veen et al. 2019) on the TDA-ready data set. The TDA Mapper algorithm and topological state algorithm include (24,48):

[TDA Mapper algorithm]

Input: X, input dataset of continuous variables
Filter X using $f : X \rightarrow Z$, where dim(Z) < dim(X)
Cover f with a finite cover U
Obtain $f^{-1}(U)$, a finite cover of X
Apply DBSCAN on the overlapping bins
Output: a simplicial complex (undirected graph) S, where nodes represent clusters and edges represent non-empty intersection between two nodes.

[Topological State definition & algorithm]

Input: Undirected Mapper graph, S
Compute density of each node of S
Output: Create a directed graph G by creating directed edges from lower density nodes to higher density nodes
Determine number of topological states by number of local maxima
Assign each site to the state associated with the nearest local maxima

The parameters of the TDA Mapper algorithm are chosen by the user and include filter function, size of the hypercubes, percent overlap, and clustering algorithm. As of 2022, there were not established methods for selecting parameters. The first two principal components (as determined from principal component analysis, PCA) were used as the filter function. As such, we used the following heuristics to

determine the optimal cube size and percent overlap parameters like Chang et al. (2020): each node must contain no more than 10% of the data and at least 90% of the data must be used in the main structure. We used the ratio between the explained variances of the first 2 PCA components of our filter function as the ratio of the components in the cube size parameter. Since the explained variances of the first two PCA components were [0.427, 0.195], we used the cube size of [273, 125]. We also used 50% overlap. For our clustering algorithm, we chose the default parameter 'DBSCAN' within Keppler Mapper. To determine DBSCAN's optimal minimum samples and epsilon parameters, we used the Elbow method that uses a minimum samples parameter of two times the number of data attributes. The epsilon values are selected by the following process:

1. Finding the average distance of each data points k-nearest neighbors (kNN)
2. Sorting and plotting the averages kNN distances for all data points
3. Finding the point of greatest curvature. Since eight water quality variables were used to produce the simplicial complex, we used 16 as our minimum samples. The epsilon value used was 1.5 and calculated using the Kneed package (Satopa et al. 2022).

The TDA Mapper outputs graphs should be consistent and insensitive to relatively small changes in parameter values. To ensure the shape of the TDA graph persists for parameter choices near the values stated above, we ran the TDA Mapper for many combinations of these parameter values: cube size ([219,100], [273.75, 125], [328.5, 150], [383.25, 175], [438, 200]) and percent overlap (30%, 40%, and 50%). As expected, the TDA Mapper graphs persisted through these parameter changes and verified our final parameter selections were appropriate.

3. Density-based state assignment

We applied a density algorithm to the undirected TDA Mapper graph to produce a directed graph with defined states as adopted and modified from Chang et al. (2020). We used k-nearest neighbor

(kNN) distance. Each node is assigned a density of $D(V) = \frac{n^2}{\sum_{i\in V} \ kNN(i,k)}$, where n^2 counts for differing node sizes and $kNN(i,k)$ is the kNN distance of point i defined as $kNN(i,k) = \frac{\sum_{j\in N(k)_i} \ d_{ij}}{k}$ where $N(k)_i$ is the set of k nearest points to the point i, k is 10% of the number of samples in the dataset, d_{ij} is the distance between points i and j. This density assignment allows converting the undirected TDA Mapper graph to a directed graph by replacing the edge between two nodes by an arrow pointing from the node with smaller density to the node with larger density. The "Topological State Definition" in algorithm 1 and Figure 5 shows how a Mapper graph is converted to a directed graph to quantify local maxima nodes.

The directed graph is then grouped into topological states (and conceptually considered ecosystem states herein), and these states are determined by *local maxima* nodes towards which every arrow connected to them points (Figure 5). In other words, the number of local maxima nodes is equal to the number of states detected. The distance between a node and a local maxima node is defined to be the smallest possible number of edges connecting them. A water quality site may sometimes be placed into more than one node, and thus classified in more than one state, if two nodes in different states have an edge between them and intersection of points. If a node is equidistant from two or more local maxima, that node is called a 'transition node' and is assigned to both local maxima (i.e., classified in both states).

4. Identifying state variables

We included eight water quality variables for the TDA to capture the multidimensionality of ecosystem state concepts. To determine state variables, we examined the gradients of each water quality variable's data ranges displayed on the TDA Mapper structure in relation to where the state was defined on the TDA Mapper graph that classified states. We also compared the data distributions among the five state classifications using boxplots for each variable. If the median (50th percentile) and boxplot

whiskers (25th–75th percentiles) did not overlap among the states, we considered that a likely state variable. We did not run formal statistical analysis for 'statistical significance' among the five boxplots (e.g., analysis of variance among the states) because the high number of data points (*n*=76,669) and degrees of freedom precluded that option.

Quantifying ecosystem state transitions

5. Temporal Correlation Function

We tested whether states were stable or transitioned over 27 years. We analyzed the state composition of each pool during each season (autumn, winter, spring, and summer). On average, each season contained 122 datapoints from each pool that were collected within a range of 15 days. We defined a temporal correlation function for a pool-state pair as:

$$f(x_{state}) = \frac{x_{state}}{n}$$

where x_{state} is the number of sites collected that lie within nodes of that state during that season, and n is the total number of sites collected during that season. In other words, the temporal correlation function is the proportion of sites in that state compared to the total number of sites collected during that season. A hypothetical example showing a state transition between two states is found in Figure 5.

ACKNOWLEDGMENTS

This work and authors DL and WB were funded as part of the U.S. Army Corps of Engineers' Upper Mississippi River Restoration Program. The National Science Foundation's "Research Experience for Undergraduates" (REU) Program funded authors CM, AS, AL, KD, FM. We sincerely thank Gregory Sandland, James Pierce, Roger Haro, and Richard Erickson for REU Program leadership and support in 2021. We thank Douglas Krouth (UWL) for initiating the first TDA Mappers. This research was performed in part using computational resources supported by the Academic & Research Computing group at

Worcester Polytechnic Institute. Any use of trade, firm, or product names is for descriptive purposes only and does not imply endorsement by the U.S. Government.

REFERENCES

1. Chapin FS, Carpenter SR, Kofinas GP, Folke C, Abel N, Clark WC, et al. Ecosystem stewardship: sustainability strategies for a rapidly changing planet. Trends Ecol Evol. 2010 Apr;25(4):241–9.
2. Harper DM. Eutrophication of freshwaters. 1st ed. London: Chapman and Hall; 1992. 1–327 p.
3. Nilsson C, Reidy CA, Dynesisu M, Revenga C. Fragmentation and Flow Regulation of the World's Large River Systems. Science (80-). 2005;308(5720):405–8.
4. Dodds WK, Whiles MR. Freshwater Ecology: Concepts and Environmental Applications of Limnology. 3rd ed. Academic Press; 2019.
5. Larson DM, Lund E, Carhart A, Drake D, Houser J, Bales K, et al. Status and Trends of Upper Mississippi River's Aquatic Vegetation from 1998 to 2019. In: Houser J, Bouska K, De Jager N, Ickes B, Jankowski KJ, Larson D, et al., editors. Status and Trends of the Upper Mississippi and Illinois Rivers. 3rd ed. U.S. Geological Sur-vey Open-File Report 2022–1039; 2022.
6. Bestelmeyer B, Ash A, Brown JR, Densambuu B, Fernández-Giménez M, Johanson J, et al. State and transition models: theory, applications, and challenges. In: Briske D, editor. Rangeland Systems: Processes, Management and Challenges [Internet]. Cham: Springer; 2017. p. 303–45. Available from: http://www.springer.com/series/412
7. Scheffer M. Ecology of shallow lakes. 1st ed. London: Kluwer Academic Publishers; 1993.
8. Larson DM, Cordts SD, Hansel-Welch N. Shallow lake management enhanced habitat and attracted waterbirds during fall migration. Hydrobiologia. 2020;846(16):3364–79.
9. Steneck RS, Graham MH, Bourque BJ, Corbett D, Erlandson JM, Estes JA, et al. Kelp forest ecosystems: biodiversity, stability, resilience and future. Environ Conserv [Internet]. 2002;29(4):436–59. Available from: https://about.jstor.org/terms
10. Briggs JM, Blair JM, Mccarron J. An ecosystem in transition:cause and consequences of the conversion of mesic grassland to shrubland. Bioscience. 2005;55(7):561–72.
11. Mumby PJ, Hastings A, Edwards HJ. Thresholds and the resilience of Caribbean coral reefs. Nature. 2007 Nov 1;450(7166):98–101.
12. Benedek Z, Jordán F, Báldi A. Topological keystone species complexes in ecological interaction networks. Community Ecol. 2007 Jun;8(1):1–7.
13. Larson DM, Dodds WK, Veach AM. Removal of Woody Riparian Vegetation Substantially Altered a Stream Ecosystem in an Otherwise Undisturbed Grassland Watershed. Ecosystems. 2019;22(1):64–76.
14. Zinnert JC, Nippert JB, Rudgers JA, Pennings SC, González G, Alber M, et al. State changes: insights from the U.S. Long Term Ecological Research Network. Ecosphere. 2021 May 1;12(5).

15. Martin BE, Walsh JR, Vander Zanden MJ. Rise of a native apex predator and an invasive zooplankton cause successive ecological regime shifts in a North Temperate Lake. Limnol Oceanogr. 2022 Feb 1;67(S1):S163–72.

16. Dent CL, Cumming GS, Carpenter SR. Multiple states in river and lake ecosystems. Philos Trans R Soc B Biol Sci. 2002 May 29;357(1421):635–45.

17. Bouska KL, Houser JN, De Jager NR, Drake DC, Collins SF, Gibson-Reinemer DK, et al. Conceptualizing alternate regimes in a large floodplain-river ecosystem: Water clarity, invasive fish, and floodplain vegetation. J Environ Manage [Internet]. 2020 Jun;264:110516. Available from: https://linkinghub.elsevier.com/retrieve/pii/S0301479720304503

18. Shang J, Zhang W, Chen X, Li Y, Niu L, Wang L, et al. How environmental stress leads to alternative microbiota states in a river ecosystem: A new insight into river restoration. Water Res. 2021 Sep 15;203:1–9.

19. Diamond JS, Moatar F, Cohen MJ, Poirel A, Martinet C, Maire A, et al. Metabolic regime shifts and ecosystem state changes are decoupled in a large river. Limnol Oceanogr. 2022 Feb 1;67(S1):S54–70.

20. Bouska KL, Houser JN, De Jager NR, Van Appledorn M, Rogala JT. Applying concepts of general resilience to large river ecosystems: A case study from the Upper Mississippi and Illinois rivers. Ecol Indic. 2019;101(January):1094–110.

21. Ibanez C, Penuelas J. Changing nutrients changing rivers. Science (80-). 2019 Aug 16;365(6454):637–8.

22. Suding KN, Gross KL, Houseman GR. Alternative states and positive feedbacks in restoration ecology. Vol. 19, Trends in Ecology and Evolution. Elsevier Ltd; 2004. p. 46–53.

23. Dale MRT. Applying Graph Theory in Ecological Research. 1st ed. Cambridge: Cambridge University Press; 2017.

24. Chang WK, VanInsberghe D, Kelly L. Topological analysis reveals state transitions in human gut and marine bacterial communities. npj Biofilms Microbiomes. 2020 Dec 1;6(1):1–10.

25. Bendick R, Hoylman ZH. Topological data analysis reveals parameters with prognostic skill for extreme wildfire size. Environ Res Lett. 2020 Oct 1;15(10).

26. Yuvaraj M, Dey AK, Lyubchich V, Gel YR, Poor VH. Topological clustering of multilayer networks. Proc Natl Acad Sci. 2021;118(21):1–9.

27. Blevins AS. Extending topological data analysis in biological systems. University of Pennsylvania; 2019.

28. Houser J, Bouska K, De Jager N, Larson D, Ickes B, Jankowski K, et al. Ecological Status and Trends of the Upper Mississippi and Illinois Rivers. U.S. Geological Sur-vey Open-File Report 2022–1039; 2022. 1–218 p.

29. McCain K, Schmueker S, De Jager N. Habitat Needs Assessment - II : Linking Science to Management Perspectives. Rock Island, IL: U.S. Army Corps of Engineers; 2018.

30. Bouska KL, Larson DM, Drake DC, Lund EM, Carhart AM, Bales KR. Aquatic vegetation dynamics in the Upper Mississippi River over 2 decades spanning vegetation recovery. Freshw Sci [Internet].

2022 Nov 18;41(1):33–44. Available from: https://www.journals.uchicago.edu/doi/10.1086/717867

31. Greenblum S, Turnbaugh PJ, Borenstein E. Metagenomic systems biology of the human gut microbiome reveals topological shifts associated with obesity and inflammatory bowel disease. Proc Natl Acad Sci U S A. 2012 Jan 10;109(2):594–9.

32. De Jager NR, Rogala JT, Rohweder JJ, Van Appledorn M, Bouska KL, Houser JN, et al. Indicators of Ecosystem Structure and Function for the Upper Mississippi River System. OFR20181143. 2018.

33. Burdis RM, DeLain SA, Lund EM, Moore MJC, Popp WA. Decadal trends and ecological shifts in backwater lakes of a large floodplain river: Upper Mississippi River. Aquat Sci. 2020 Apr 1;82(2):1–19.

34. Mason TJ, Keith DA, Letten AD. Detecting state changes for ecosystem conservation with long-term monitoring of species composition: Ecol Appl. 2017 Mar 1;27(2):458–68.

35. Islambekov U, Yuvaraj M, Gel YR. Harnessing the power of topological data analysis to detect change points. In: Environmetrics. John Wiley and Sons Ltd; 2020.

36. Hilt S, Köhler J, Kozerski HP, van Nes EH, Scheffer M. Abrupt regime shifts in space and time along rivers and connected lake systems. Oikos. 2011 May;120(5):766–75.

37. Scheffer M, Bascompte J, Brock W, Brovkin V. Early-warning signals for critical transitions. Nature [Internet]. 2009 [cited 2017 Aug 21];461(7260):53–9. Available from: http://search.proquest.com/openview/7734e5b77c0e9dd2198216184823e65d/1?pq-origsite=gscholar&cbl=40569

38. Suding KN, Hobbs RJ. Threshold models in restoration and conservation: a developing framework. Vol. 24, Trends in Ecology and Evolution. 2009. p. 271–9.

39. Scheffer M, Carpenter SR. Catastrophic regime shifts in ecosystems: Linking theory to observation. Trends Ecol Evol. 2003;18(12):648–56.

40. Biggs R, Carpenter SR, Brock WA. Turning back from the brink: Detecting an impending regime shift in time to avert it. Proc Natl Acad Sci [Internet]. 2009;106(3):826–31. Available from: www.pnas.org/cgi/content/full/

41. Jiang J, Huang ZG, Seager TP, Lin W, Grebogi C, Hastings A, et al. Predicting tipping points in mutualistic networks through dimension reduction. Proc Natl Acad Sci U S A. 2018 Jan 23;115(4):E639–47.

42. Samhouri JF, Levin PS, Ainsworth CH. Identifying thresholds for ecosystem-based management. PLoS One. 2010 Jan 26;5(1):1–10.

43. U.S. Army Corps of Engineers. Upper Mississippi River Restoration Program [Internet]. 2022 [cited 2022 Aug 29]. Available from: https://www.mvr.usace.army.mil/Missions/Environmental-Stewardship/Upper-Mississippi-River-Restoration/

44. U.S. Environmental Protection Agency. Ambient Water Quality Criteria Recommendations: Information Supporting the Development of State and Tribal Nutrient Criteria Lakes and Reservoirs in Nutrient Ecoregion IX. 2000;EPA 822-B-00-019(December):1–108.

45. Capon SJ, Lynch AJJ, Bond N, Chessman BC, Davis J, Davidson N, et al. Regime shifts, thresholds

and multiple stable states in freshwater ecosystems; a critical appraisal of the evidence. Sci Total Environ. 2015 Nov 15;534:122–30.

46. Groffman PM, Baron JS, Blett T, Gold AJ, Goodman I, Gunderson LH, et al. Ecological thresholds: The key to successful environmental management or an important concept with no practical application? Ecosystems. 2006;9(1):1–13.

47. Soballe DM, Fischer JR. Long Term Resource Monitoring Program Procedures: Water Quality Monitoring. U.S. Army Corps of Engineers Technical Report 2004-T002-01; 2004. 1–134 p.

48. Singh G, Mémoli F, Carlsson G. Topological Methods for the Analysis of High Dimensional Data Sets and 3D Object Recognition. Eurographics Symposium on Point-Based Graphics. Eurographics Symposium on Point-Based Graphics; 2007.

FIGURES

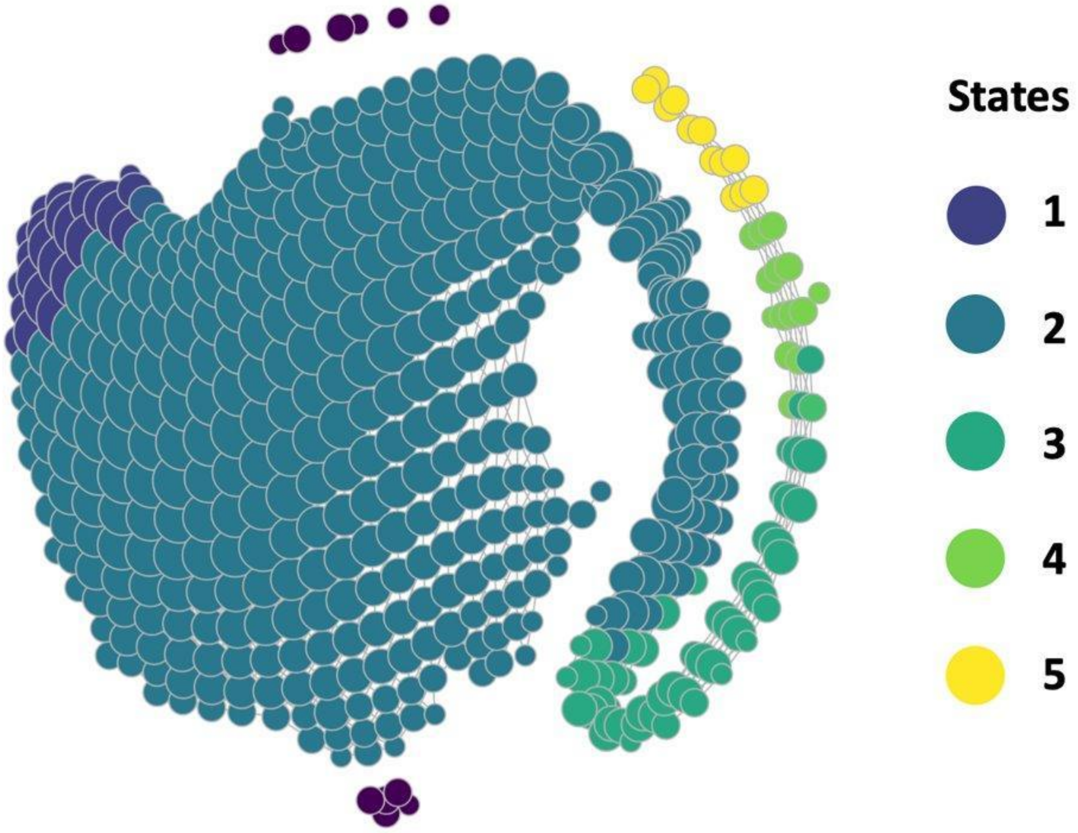

Figure 1. Graphical output from topological data analysis (TDA Mapper) of the potential ecosystem states for the Upper Mississippi River System, USA. Data inputs included 8 water quality state variables collected four times each year from 1993–2020 at 69,307 sampling sites. TDA Mapper showed five topological states (color coded, which can be conceptualized as ecosystem states), and most sites were within State 2. The dark purple nodes not connected to the main structure (<1% of sites) were outliers and not classified within a state.

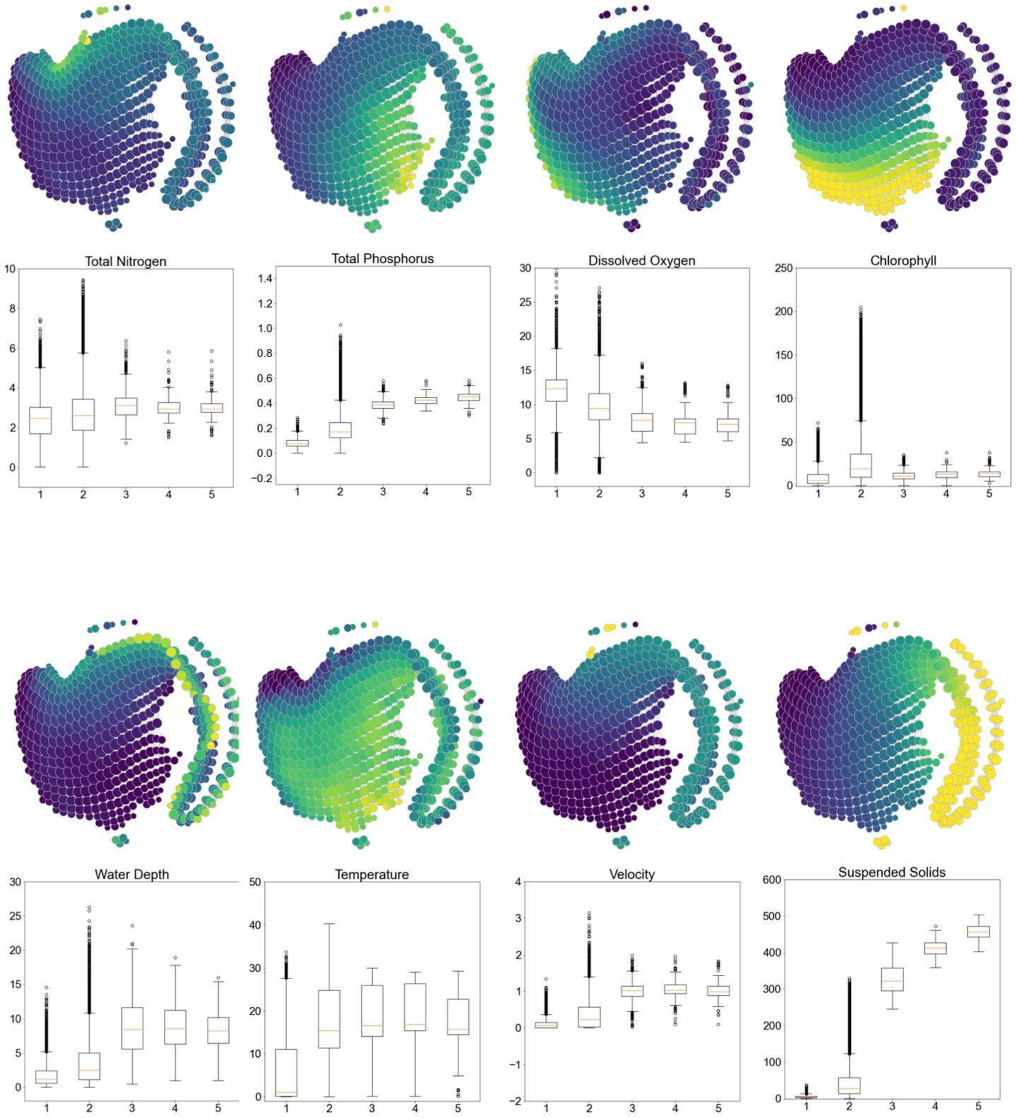

Figure 2. Graphical outputs from topological data analysis (TDA Mapper) showing ecological gradients of 8 water quality state variables in the Upper Mississippi River System, USA (*n*=69,307 sampling sites from 1993–2020). The color coding represents relative values for each variable; dark violet colors are relatively low values, whereas greens and yellows are relatively high values. The boxplots below each TDA Mapper graph showed the data distributions for each ecosystem state (that corresponds to the states in Figure 1). Boxplots are standardized by boxes (containing the 25th, 50th (median), and 75th percentiles), the whiskers (containing the 0th and 100th percentiles, excluding outliers), and the dots (statistical outliers). The x-axis on the boxplots represents the five states (States 1–5). The y-axis represents variable units (total nitrogen = mg L^{-1}; total phosphorus = mg L^{-1}; dissolved oxygen = mg L^{-1}; chlorophyll *a* = µg L^{-1}; water depth = cm; temperature = °C; velocity = m sec^{-1}; suspended solids = mg L^{-1}.

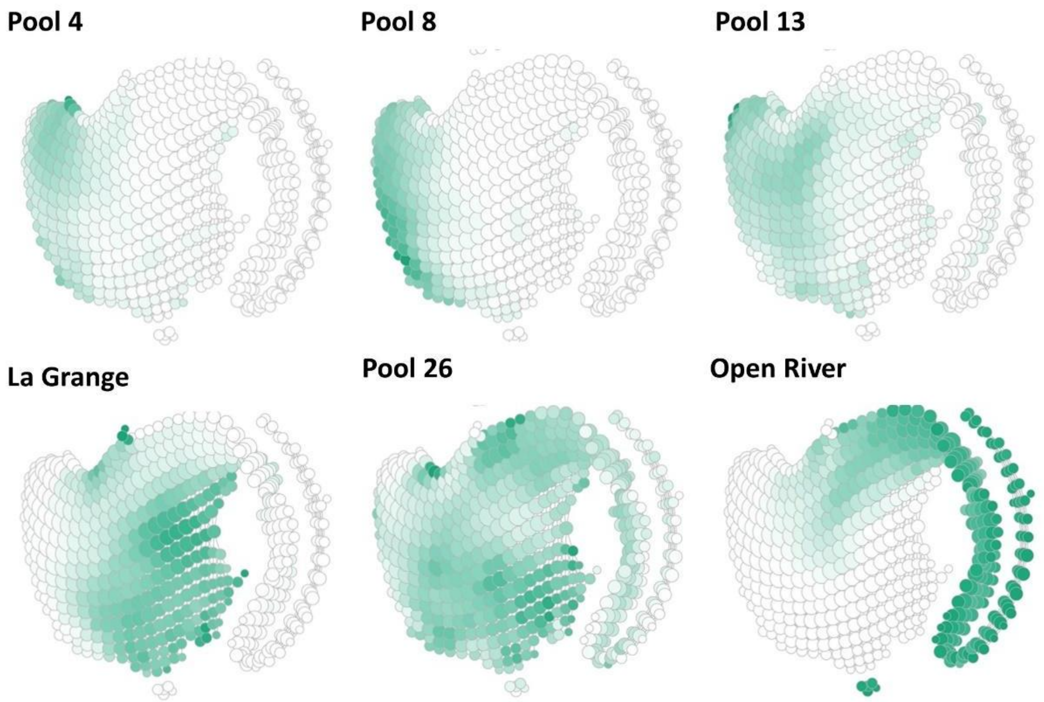

Figure 3. Topological data analysis (TDA) showed one primary data structure for 8 water quality state variables from six navigation pools on the Upper Mississippi River System (years 1993–2020), and the green colors highlighted where sampling sites within each pool grouped onto the primary TDA Mapper structure. Dark green nodes had relatively many sites within the node compared to the lighter green nodes containing fewer sites. Different positions on these TDA Mapper graphs were highlighted in green according to pool, indicating these pools have belong to different ecosystem states based on water quality. Pools can be classified into specific ecosystem states by referencing their positions within Figure 2; for example, the Pool 4 was within States 1 and 2 because the green highlights are on the left side of the TDA structure (panel a). As another example, Open River pool was principally within States 3, 4, and 5 because the green highlights are on the right side of the TDA structure and its tail (panel f). Pools can also be characterized by water quality state variables in Figure 3; for example, Open River pool is typically characterized by high velocity and suspended solids.

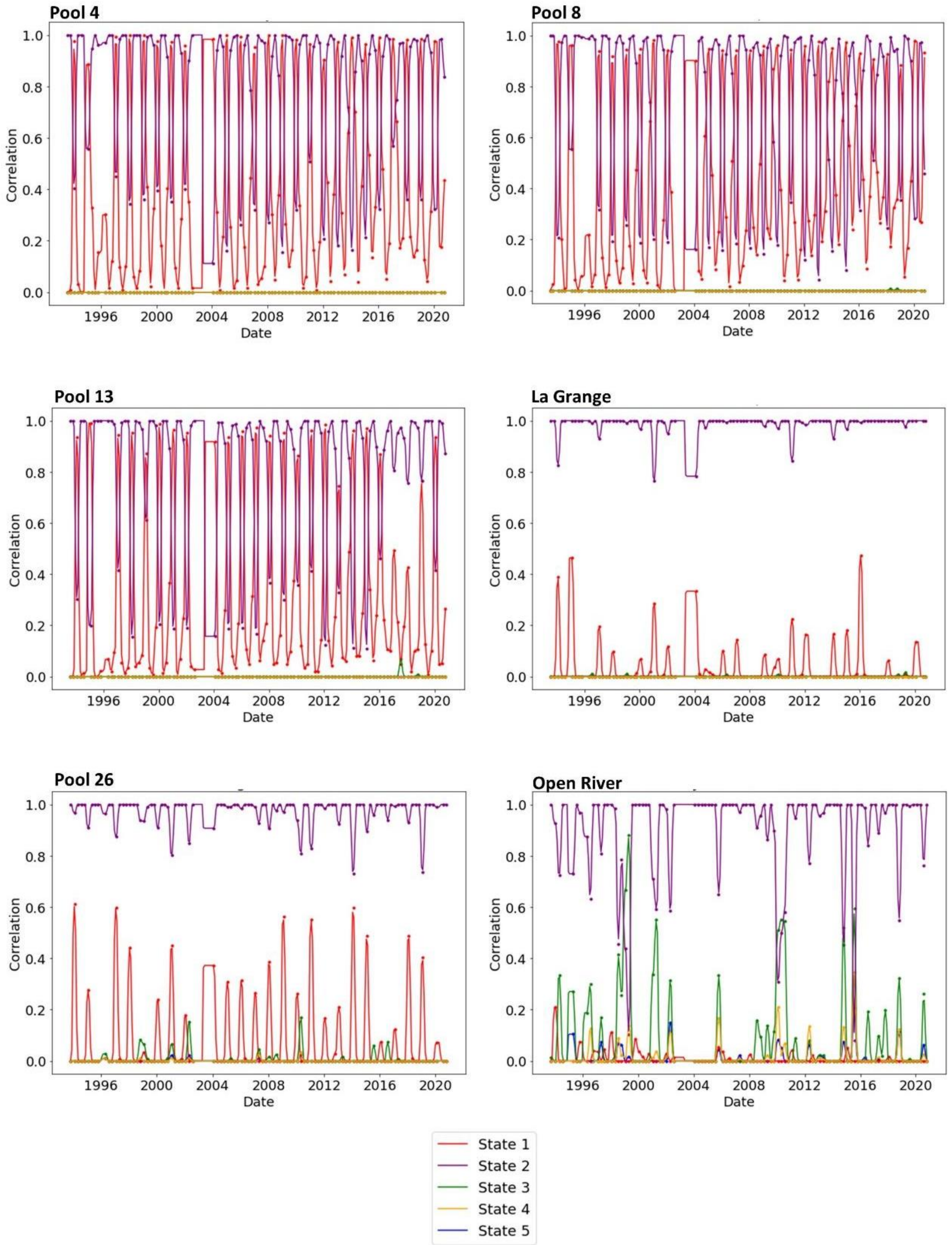

Figure 4. Temporal correlation function plots showed the transitions in ecosystem states within six pools of the Upper Mississippi River System from years 1993–2020. The correlation (y axes) is the proportion of sites within each state compared to the number of sites collected during that season (~125 sites per season per year per pool). Occasionally, sites were classified into multiple states, and therefore the correlation sum may exceed 1.0. If correlation lines from

States 1–5 never crossed in the time series, that pool had state stability and had not experienced state changes. If correlation lines crossed temporarily, this indicated instable and transient dynamics due to seasonal effects or episodic events. If a state had a monotonic decrease in correlation and then correlation lines crossed, this could have indicated a long-lasting state change or ecological 'regime shift.' Points that fall directly on the year were collected in winter, followed by spring, summer, and autumn samples. Data from 2003 was missing due to logistical constraints for collection.

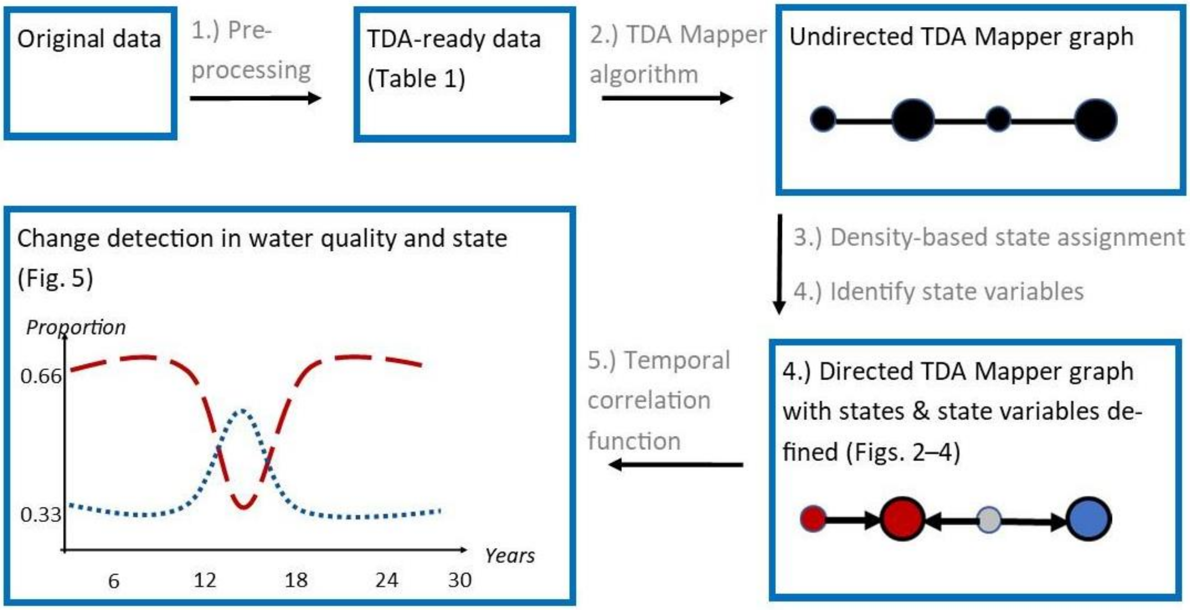

Figure 5: Flow chart that summarized our topological data analysis (TDA) methods in sequential order (arrows and gray text) and outputted products (boxes with black text and images) for quantifying ecological states and state transitions of the Upper Mississippi River. The original- and TDA-ready datasets are available here (DOI: XXXX). In the “Undirected TDA Mapper” box, nodes and edges are connected to create a single TDA structure. In “Directed TDA Mapper” box, the example output describes two local maxima nodes defining two states: a red state containing two nodes and a blue state containing one node. The grey node is a transition node and does not belong to state. In the “change detection” box, we provide a hypothetical output of where the dominance of two states (red and blue dashed lines, which are the proportion of sites within those states) temporarily transition from the dominant red state to the blue state around years 13 to 17.
