# Supplementary material for: Quantifying ecosystem states and state transitions of the Upper Mississippi River System using topological data analysis
Source: PLoS Comput Biol. 2023 Jun 7;19(6):e1011147. doi: 10.1371/journal.pcbi.1011147 (PMC10246787; doi:10.1371/journal.pcbi.1011147)
Supplement: Attachment — Submitted filename: PCOMPBIOL-D-22-01462_R1_rev_v3.pdf [file pcbi.1011147.s005.pdf]

PLOS Computational Biology

# Quantifying ecosystem states and state transitions of the Upper Mississippi River System using topological data analysis

--Manuscript Draft--

| | |
|---|---|
| **Manuscript Number:** | PCOMPBIOL-D-22-01462R1 |
| **Full Title:** | Quantifying ecosystem states and state transitions of the Upper Mississippi River System using topological data analysis |
| **Short Title:** | quantifying riverine states via topology |
| **Article Type:** | Research Article |
| **Keywords:** | alternative stable ecological state; ecological regime shift; ecosystem state change; topology; topological state; water quality; vulnerability |
| **Abstract:** | Aquatic systems worldwide can exist in multiple ecosystem states (i.e., a recurring collection of biological and chemical attributes), and effectively characterizing multidimensional states will aid protection of desirable states and guide restoration. The Upper Mississippi River System is a large, floodplain river spanning 2200 km with a 30-year water quality monitoring data set and and ecosystem states need depicting that can guide river restoration. We coupled this highly dimensional dataset with multiple topological data analysis (TDA) techniques to classify ecosystem states, identify state variables, and detect state transitions over 30 years. Across the entire system, TDA identified five ecosystem states. State 1 was characterized by exceptionally clear, clean, and cold-water conditions typical of winter (i.e., a ‘clear-water state’); State 2 had the greatest range of environmental conditions (i.e., a ‘status-quo state’); and States 3, 4, and 5 had extremely high concentrations of suspended solids (i.e., ‘turbid-states’, with State 5 as the most turbid). The TDA mapped clear patterns of the ecosystem states across several riverine navigation reaches and seasons that furthered ecological understanding. State variables were identified as suspended solids, chlorophyll a, and total phosphorus, which are also state variables of shallow lakes worldwide. The TDA change detection function showed short-term state transitions based on seasonality and episodic events, and also provided evidence of gradual, long-term changes due to water quality improvements over three decades. These results can inform decision making and guide actions for regulatory and restoration agencies by assessing the status and trends of this important river and provide quantitative targets for state variables. The TDA change detection function may serve as a new tool for predicting the vulnerability to undesirable state transitions in this system and other ecosystems with sufficient data. Coupling ecosystem state concepts and TDA tools can be transferred to any ecosystem with large data to help classify states and understand their vulnerability to state transitions. |
| **Additional Information:** | |
| **Question** | **Response** |
| **Government Employee**<br><br>Are you or any of the contributing authors an employee of the United States government?<br><br>Manuscripts authored by one or more US Government employees are not copyrighted, but are licensed under a CC0 Public Domain Dedication, which allows unlimited distribution and reuse of the article for any lawful purpose. This is a | Yes - One or more authors are employees of the U.S. government. |

Powered by Editorial Manager® and ProduXion Manager® from Aries Systems Corporation

| | |
|---|---|
| legal requirement for US Government employees.<br><br>This will be typeset if the manuscript is accepted for publication. | |
| **Financial Disclosure**<br><br>Enter a financial disclosure statement that describes the sources of funding for the work included in this submission and the role the funder(s) played. This includes grants and any commercial funding of the work or authors.<br><br>This statement will be typeset if the manuscript is accepted for publication.<br><br>Review the submission guidelines and the instructions link below for detailed requirements and guidance. | The salaries of authors DL and WB, the data collection, data analysis, and decision to publish were funded as part of the U.S. Army Corps of Engineers' Upper Mississippi River Restoration Program. The National Science Foundation's "Research Experience for Undergraduates" Program Award Number:1559663 funded authors CM, AS, AL, KD, FM for data analysis and manuscript preparation. |
| **Competing Interests**<br><br>On behalf of all authors, disclose any competing interests that could be perceived to bias this work.<br><br>This statement will be typeset if the manuscript is accepted for publication.<br><br>Review the instructions link below and PLOS Computational Biology's competing interests policy to determine what information must be disclosed at submission. | none |
| **Data and Code Availability**<br><br>From the time of publication, Authors are required to make fully available and without restriction all data and computational code underlying their findings. Please see our PLOS Data Policy page for detailed policy information, and our Code Sharing page for specific information on code sharing. | All data were publicly accessed on 1 June, 2021 through the Upper Mississippi River Restoration Program's Long-term Resource Monitoring (https://umesc.usgs.gov/ltrm-home.html). Our final datasets and analysis scripts are publicly and permanently archived in ScienceBase (DOI: 10.5066/P976O6PP). |

*Powered by Editorial Manager® and ProduXion Manager® from Aries Systems Corporation*

| | |
|---|---|
| A **Data Availability Statement**, detailing where the data (and code, if applicable) can be accessed, is required at first submission. Insert your Data Availability Statement in the box below. The statement you provide **will be published in the article**, if accepted.<br><br>Please see the Data Reporting section of our submission guidelines for instructions on what you need to include in your Data Availability Statement.<br><br>If the data and code are all contained in your submission files, please state: *All relevant data are within the manuscript and its Supporting Information files.*<br><br>**PLOS allows rare exemptions to address legal and ethical concerns.** If you have legal or ethical restrictions, please detail these in your Data Availability Statement below for the Journal team to consider. | |

Powered by Editorial Manager® and ProduXion Manager® from Aries Systems Corporation

**From:** em.pcompbiol.0.80eca6.f3efa0ea@editorialmanager.com
<em.pcompbiol.0.80eca6.f3efa0ea@editorialmanager.com> on behalf of PLOS Computational Biology
<em@editorialmanager.com>
**Sent:** Tuesday, January 24, 2023 8:20 PM
**To:** Larson, Danelle M <dmlarson@usgs.gov>
**Subject:** [EXTERNAL] Decision from PLOS Computational Biology (PCOMPBIOL-D-22-01462) - [EMID:177b034513490cb9]

CC: "Wako Bungula" wkbungula@uwlax.edu, "Casey McKean" caseyjmckean@gmail.com, "Alaina Stockdill" astockdill22@my.whitworth.edu, "Amber Lee" apla2018@mymail.pomona.edu, "Frederick Miller" fimiller@wpi.edu, "Killian Davis" killiad@g.clemson.edu

Dear Dr. Larson,
Thank you very much for submitting your manuscript "Quantifying ecosystem states and state transitions of the Upper Mississippi River using topological data analysis" for consideration at PLOS Computational Biology.
As with all papers reviewed by the journal, your manuscript was reviewed by members of the editorial board and by several independent reviewers. In light of the reviews (below this email), we would like to invite the resubmission of a significantly-revised version that takes into account the reviewers' comments.

We cannot make any decision about publication until we have seen the revised manuscript and your response to the reviewers' comments. Your revised manuscript is also likely to be sent to reviewers for further evaluation.
When you are ready to resubmit, please upload the following:
[1] A letter containing a detailed list of your responses to the review comments and a description of the changes you have made in the manuscript. Please note while forming your response, if your article is accepted, you may have the opportunity to make the peer review history publicly available. The record will include editor decision letters (with reviews) and your responses to reviewer comments. If eligible, we will contact you to opt in or out.
[2] Two versions of the revised manuscript: one with either highlights or tracked changes denoting where the text has been changed; the other a clean version (uploaded as the manuscript file).
Important additional instructions are given below your reviewer comments.
Please prepare and submit your revised manuscript within 60 days. If you anticipate any delay, please let us know the expected resubmission date by replying to this email. Please note that revised manuscripts received after the 60-day due date may require evaluation and peer review similar to newly submitted manuscripts.
Thank you again for your submission. We hope that our editorial process has been constructive so far, and we welcome your feedback at any time. Please don't hesitate to contact us if you have any questions or comments.
Sincerely,

Eric Lofgren, MSPH, PhD
Academic Editor
PLOS Computational Biology

Natalia Komarova
Section Editor
PLOS Computational Biology

************************

Reviewer's Responses to Questions
**Comments to the Authors:**
**Please note here if the review is uploaded as an attachment.**

**Reviewer #1**: The article is well organized, and the method section is clearly described. Excellent, thank you for your careful review and thoughtful questions and suggestions.

Can you please clarify the following questions:

1) In line 376: "To address missingness, we compared seven interpolation methods and utilized a random forests algorithm as the top preforming method"-- Correct "preforming" ->"performing". Can you please show the top performance value and which metric you used as the performance metrics? Also what other methods are used along with random forest? Absolutely. We hope a paper describing the process is available soon, but we now better describe the interpolation process in this paper as well. We revised to read: "The TDA does not allow missing values, therefore we compared multiple interpolation methods including kriging, inverse distance weighting of various distance thresholds, polynomial regression of various degrees, regression trees, and random forests. We chose a random forests algorithm as the top performing interpolation method (when compared to the mean absolute error to the other methods), which had > 93% accuracy for all interpolated variables. The other occasional missing values were imputed with the median value for each variable."

2) In line 401: "As of 2022, there were not established methods for selecting parameters". Persistence homology can be used to find the appropriate overlapping value and stable TDA object. The supplementary part of this paper (https://ieeexplore.ieee.org/document/8880514) shows how persistence homology helps to find a stable TDA object, and you can refer to this paper. We thank the reviewer for this idea and enjoyed the referenced paper. We note that the two algorithms are different: the TDA Mapper (which we used) and the persistence homology ("PH", which are the basis in this reference paper). The growing circles around each data point overlap in PH, whereas the pre-image of the cover of *f*, filter function, overlap in Mapper. The reviewer is correct in saying that a radius (the only parameter in PH) can be chosen that gives a stable object. If we used PH and found a radius, we would still lack the key parameters in TDA Mapper like the number/size of intervals (to cover the image of *f).* We don't think PH and Mapper are compatible given they are different equations with different parameters, but now reference the paper in this line to be thorough.

**<u>Reviewer #2:</u>** review is uploaded as an attachment
We found the email attachment with reviewer 2's comments. We copied that here to keep reviews consolidated.

This study focuses on analysis of a large-scale publicly-available dataset of water quality metrics collected from multiple sites across the Mississippi River over a span of 30 years. The authors' goal is to synthesize the multi-dimensional data into meaningful classifications of water quality 'states'. To do so, they apply topological data analysis (TDA) and identify 5 main ecosystem states, one of which they deem 'healthy', while the others were indicative of varying levels of 'undesirable' conditions. The authors propose 5 hypotheses relevant to this analysis, and briefly address each in the text. According to the authors, this is the first application of TDA to a river ecosystem, and they expect that this analysis will help those invested in the river's health to better communicate river conditions and make decisions about restorative actions.

While the use of TDA to better understand the river system seems appropriate, this study fell short on a number of levels. 1) The methods were poorly described such that replicating the analysis would be impossible, 2) the interpretation of the results was often vague and not backed by statistics, 3) the analysis itself was very limited in scope, and 4) the discussion overstates the usefulness and generality of the findings. We sincerely thank anonymous reviewer #2 for their review and synthesis here. We carefully assessed the critiques and made improvements when possible throughout. We address each of these listed points where they are repeated below.

1) The methods section states that data was downloaded from the usgs.gov website, and originally contained 204,345 rows (observations) and 133 columns (measured water quality variables). The description of how the data was filtered is very vague (p18. lines 369-374: *We filtered the data by stratified random sampling scheme, surface water, and 8 continuous variables. …)*, yet after filtering, the '*final data size was 69,307 rows by 8 columns'*. It is completely unclear how and why the 204k observations were filtered to only 69k, and they do provide an account of which sites were included in the final set. Thank you. The data pre-processing steps will be fully available in the RMarkdown analysis scripts that we provide, so a user could fully replicate the study. In the methods (lines 448-449), we added more detail: "We filtered the data by stratified random sampling scheme (and filtered out 'fixed sample sites', which contained ~60% of the data set), surface water collections…" Furthermore, of the 133 variables, the authors '*chose eight water quality variables thought to be important for defining ecosystem states*' (p17, ln 344-5). Why? Isn't the point of TDA to find patterns in highly multidimensional data, and determine which suites of variables are important? The TDA could handle including all 133 variables (if they are not highly correlated), but ecological relevance and ecological interpretation of the outputs would be questionable. We suggest it's important to cull variables to those ecologically relevant. We added to lines 413-419: "Using ecological literature and *a priori* hypotheses (as stated in the Introduction), we chose eight water quality variables thought to be important for defining ecosystem states and ensured the variables were not highly correlated (Pearson's $r < 0.45$). The 8 variables included: total nitrogen = mg $L^{-1}$; total phosphorus = mg $L^{-1}$; dissolved oxygen = mg $L^{-1}$; chlorophyll *a* = µg $L^{-1}$; water depth = cm; temperature = °C; velocity = m $sec^{-1}$; suspended solids = mg $L^{-1}$. Although the TDA can incorporate more than 8 variables, we carefully selected 8 variables based on ecological relevance to state theory and those with management applications to manipulate in restoration (e.g., water velocity and nutrient inputs; see McCain et al. 2018)." The authors say they tested

seven interpolation methods to address missingness but give no further details or results, nor do they provide any detail on how the one they chose (random forest algorithm) was implemented. We added several sentences to explain the interpolation process and decisions. We also have a paper with analysis in press to cite with more details now. Thank you! Lines 454-459 were added to clarify: "Three of the variables (total nitrogen, total phosphorus and velocity), per the study design, were only collected at two thirds of the sampling sites. The TDA does not allow missing values, therefore we compared multiple interpolation methods including kriging, inverse distance weighting of various distance thresholds, polynomial regression of various degrees, regression trees, and random forests. We chose a random forests algorithm as the top performing interpolation method (when compared to the mean absolute error to the other methods), which had > 93% accuracy for all interpolated variables (Larson et al. in press). The other occasional missing values were imputed with the median value for each variable. The points above are major omissions in just the pre-processing section – the rest of the methods section was not any clearer. We have revised the entire methods section with more details and improved grammar. We now repeatedly reference Figure 5 that shows the roadmap of many different processes and outputs. We will also share RMarkdown codes with every processing step that contains notes with justification and interpretations.

2) TDA was performed and identified 5 states, but no confidence metrics were reported. We appreciate this suggestion, as confidence metrics are always preferred when possible. We carefully considered this suggestion but determined that quantifying confidence metrics is highly involved and requires advanced super-computing power that we don't have access to currently. We also suspect that our dataset (n=70,000 sites) is not large enough for confidence assessment (Carriere et al. 2018; 17-291.pdf (mit.edu)). Although the modules of continuity (omega) for our PCA filter function is known (specifically: 1-Lipschitz), we cannot use the confidence set because our data set is very likely not large enough (Carriere et al. 2018); this paper used 10 million data points! However, we improved our writing to show we have confidence in the results because we chose a sequence of parameters (intervals, percent overlap, epsilon / DBSCAN) and the shape persisted with many parameter combinations, as described on lines 477-487. So, we are confident (without formal confidence metrics) that the TDA Mapper algorithm captured the true shape of the underlying data space. The authors report that 'total nitrogen was the only variable that didn't vary across the five state classifications' (line 171) but no statistics are used to validate that assertion, which they used to support one of their hypotheses. The statement of total nitrogen not representing a state variable was supported by reference to Figure 2, which has boxplots. The boxplots show the statistical distribution of data, and the distribution was very similar across all five states. We discussed running an analysis of variance (ANOVA) statistical test on the boxplots among states, but visually the trends are clear and we have too much data for traditional statistics, which would cause a Type 1 error. We discuss in the methods how to we used statistics to determine state variables (lines 539-549). Similarly no statistics we provided to support the claims that 'State 1 dominated in winter most of the years' (line 222) or that 'the increased minimum correlation values over time in State 1 was less pronounced than in Pools 4 and 8' (line 227-8). We added a supplemental figure (S1 Figure) to show that state 1 is typically in winter, thank you. The correlation/proportion values are the formal test statistic for the 'temporal correlation analysis' (now called 'change detection analysis'), and the mathematics are described in the Methods. We are unsure what types of statistics would improve reader confidence of these results. We reference Figure 4 to back the claim that increased minimum correlation values were less pronounced in Pool 13. These are just a few examples of many - overall no formal downstream analysis was performed beyond obtaining results from the TDA mapper algorithm.

We respectfully disagree with the review on this point. We have went beyond TDA Mapper by employing a topological states definition to refine and further explores states, identify state variables, and assess state transitions over 30 years. Together, this was a robust, quantitative approach for the research questions (see Figure 5 roadmap). We are receptive to suggestions for next steps for statistical analysis, but the reviewer did not suggest what types of analysis would improve support for our hypotheses. However, we have very carefully revised the Materials and Methods for clarity and now better reference Figure 5 that shows that many analyses and outputs we delivered.

3) The TDA results could have been used in a number of ways to provide insight into key markers of turning points in ecosystem health, and an assessment of the accuracy of these markers for future predictions or other systems. We appreciate the prospects for furthering improving markers of ecosystem health based this comment, but do not understand how to use the TDA in these new ways. More elaboration would help us to understand and possibly implement these ideas for this paper. Instead, the authors state that: '*the states could become a categorical response variable in future models that aim to assess the environmental drivers and threshold responses'* (line 315) yet do not perform this analysis themselves, and '*The TDA also allows extraction of information at finer spatiotemporal scales but was beyond the scope of this paper'* (line 276-7). We gave ideas how TDA could further be used, but feel that its outside the scope and word limits to do this here. The introduction of three TDA tools to define ecosystem states and detect state transitions was substantial contribution and implementing these ideas would be too much. The authors did not remark on whether these same states might apply to other river systems or do any analysis of whether there were meaningful substates within the 5 main states. We agreed here on expanding results to other rivers. First, we added a full new paragraph to the discussion (lines 365-378): "Ecologists have emphasized the need to apply ecosystem states concepts to rivers worldwide, while acknowledging most rivers do not have sufficient data to properly evaluate states (Biggs et al. 2018, Bouska et al. 2019, Ibanez and Penuelas 2019). The Mississippi River has exceptional long-term data, and our results supported the concepts of alternative states in this particular river (Bouska et al. 2020). We suspect that similar ecosystem states based on water quality state variables can be found in other rivers, although we are unaware of other case studies to-date other than 'clear-water' and 'turbid-states' based on aquatic plant abundance and water quality state variables (Ibanez and Penuelas 2019). The Regime Shifts Database (Biggs et al. 2018) could serve as a data source to test our TDA ecosystem states framework in other rivers with similar water quality data (e.g., known hypoxia regime shifts within the Amite River, Brazo River, Chester River, and Indian River in the USA)."

In response to the substates concern, we added to line 308: "Although we could have altered the TDA density algorithm to explore more sub-states within these five states, we didn't have justification for refinement and wanted to provide a reasonable amount of states that were clearly differentiated for restoration applications (McCain et al. 2018)."

We also better explain in the Methods sections 3. And 4. how we have a undirected graph (producing 2 states) and a directed graph (producing 5 states, or substates within the undirected graph); also see Figure 5.

4) The authors claim these results will be useful for making decisions about restorative actions for the Mississippi river, but do not give any clear examples of how one would go about this. Their discussion section on transferability of TDA to other ecosystems says that *'TDA may provide an*

*early warning indication of vulnerability'*, but do not say what those warnings might be in this system, or whether the same variables would be a warning in other systems. Good point. We clarified in the Discussion when we reference the Mississippi River versus other aquatic systems more generally. We also added on lines 357-360: "The state variables of total phosphorus, chlorophyll a, and suspended solids identified for the Mississippi River are the same as those of lake ecosystems (Scheffer 1993), and so assessing vulnerability to state transitions using these variables could be a warning in other aquatic systems beyond the Mississippi River." If the whole analysis must be performed from scratch to gain insight into other systems, it is unclear whether the methodology is in fact generalizable; since this dataset is exceptional in terms of scope and depth of data collection, it seems unlikely that this same analysis would succeed in other less well-sampled systems. The last sentence of the paper is that *'state concepts and TDA methodologies can be transferred to any ecosystem …'* but that was true regardless of the work presented here. Overall it is unclear whether this work offers a significant contribution. We carefully considered and addressed all these comments here. The Discussion (all sections) was heavily revised and includes several new paragraphs to describe the novelty and transferability of the TDA approach in other systems. We provide examples of how our findings can inform restorations, including defining the ecosystem states, determining which are desirable or not, setting quantitative targets for restoration and water quality regulations, and how assessing vulnerability can aid in prioritizing restorations. Thank you.

Other points:

- The phrase 'state variable' was used many times, but the definition was never given, nor were criteria defined for. Agreed. We created a new paragraph in the Introduction (lines 79-88) that define ecosystem states, state variables, and cite seminal references. We also now better describe in the Methods (lines 539-549) how we used TDA Mapper and data distribution statistics to define 'state variables.' Why were three state variables hypothesized (Line 109)? What was this number based on? Why were 8 variables chosen for the analysis? We added a full paragraph devoted to why 8 variables were chosen in the analysis, beginning on line 413: "Using ecological literature and *a priori* hypotheses (as stated in the Introduction), we chose eight water quality variables thought to be important for defining ecosystem states and ensured the variables were not highly correlated (Pearson's $r < 0.45$). The 8 variables included: total nitrogen = mg $L^{-1}$; total phosphorus = mg $L^{-1}$; dissolved oxygen = mg $L^{-1}$; chlorophyll *a* = µg $L^{-1}$; water depth = cm; temperature = °C; velocity = m $sec^{-1}$; suspended solids = mg $L^{-1}$. Although the TDA can incorporate more than 8 variables, we carefully selected 8 variables based on ecological relevance to state theory and those with management applications to manipulate in restoration (e.g., water velocity and nutrient inputs; see McCain et al. 2018)."
- The methods should be summarized succinctly in the main text so the description of results has context. As is, the first sentence of the results section is '*The TDA Mapper graph output has six connected components…*' (line 118), but neither the TDA Mapper tool itself nor its expected output has been described. What do nodes and components represent? We have a detailed "Materials and Methods" section within the main text. We heavily revised this based on reviewer comments for more details, including describing TDA Mappers and its nodes and connections. For the target journal PLOS Computational Biology, they require the "Materials and Methods" section after the results.

- Line 125 (and elsewhere) states that there were 69,307 sites, while line 130 states that there were 76,669 sites – which is correct? Thank you for the notice to detail. The correct sample size is 69,307. We corrected this throughout the manuscript.
- The caption for Table 1 is not sufficient to clearly explain the difference between the two tables (1a and 1b) – this is made more confusing by the fact that '%' signs are included in 1a but not 1b, while both are supposed to be proportions. We apologize and revised the figure caption to explain the differences of Table 1a and 1b. We removed the % symbol in the table, and changed 'proportion' to 'percentage.'
- Captions for figures 3 and 4 should contain description of the plots, not interpretation – that belongs in the main text. We removed all interpretation for the figure captions.
- Line 310: *The TDA tool indicated Pool 13 may be vulnerable to transitioning to the undesirable turbid state (State 3; Figure 4) because it has transitioned to State 3 repeatedly only in the last few years of a 30-year record*… So, transitioning to state 3 is an early warning sign of transitioning to state 3? Yes, the statement was not insightful. We deleted this sentence and replaced with: "The TDA tool detected changes to the variability of the number of sites within a state as the correlation values, and a change in time series variability can indicate ecological transitions (Carpenter and Brock, 2006). The TDA could detect if more sites within Pool 13 continue to enter State 3 more frequently or persistently in the near future."
- Line 451-453: *We did not run formal statistical analysis for 'statistical significance' among the five boxplots (e.g., analysis of variance among the states) because the high number of data points (n=76,669) and degrees of freedom precluded that option.* It does not make sense to me why statistical tests were not used to compare the distributions; a large number of observations should not be a problem. A large number of observations used in a traditional statistics (like analysis of variance) will cause Type 1 error. We added on line 549: "We did not run formal statistical analysis for 'statistical significance' among the five boxplots (e.g., analysis of variance among the states) because the large number of data points (*n*=76,669) and degrees of freedom precluded that option due to risk of Type 1 error (false positive)."
- Furthermore, *n* is reported elsewhere as 69,307 – where did the number reported here come from? Thank you for the notice to detail. The correct sample size is 69,307. We corrected this throughout the manuscript.
- Lines 455-464: The authors define a 'temporal correlation function' as simply the proportion of all measurements that are assigned to a given state. I do not see how this can be called a 'correlation' – yet this is exactly what is plotted in Figure 4, and the y-axis is labeled 'correlation'. We agree that 'correlation' wasn't the best term. We initially used a temporal correlation function and used the term 'correlation values' based on previous literature (Chang et al. 2020). We had subsequently modified the original temporal correlation function, and so now call that a 'change detection function' as described in Methods subsection 5. Further, 'correlation values' were changed to 'proportion values' in the text and figures as you suggested. Thank you much!

**Reviewer #3:** Review of Larson et al. Quantifying ecosystem states and state transitions of the Upper Mississippi River using topological data analysis.

The authors present the results of a novel application of topological data analysis (TDA) – its use to assess a large data set for evidence consistent with the existence of differing ecological states. The quantitative method was able to classify data from ~70K site visits as belonging to one of 5 classes. The authors interpret those results as indicating the classes represent 5 ecosystem states. Starting with eight candidate variables, the analysis indicated that the primary variables determining the ecosystem classification were: suspended solids, chlorophyll a and total phosphorus. Temporal patterns were also assessed for evidence of abrupt changes in the ecological state; results suggested that changes were better described as gradual than abrupt. We thank the reviewer for the clear and accurate synopsis of this paper. The quantitative results are for the most part clearly explained (exceptions noted in my attached comments), are consistent with other studies of this system, and new insights. The figures and text provide reasonable support for the quantitative results. As described in my general comments below, there are some aspects of the conceptual interpretation of those quantitative results that need to be better explained and possibly more carefully considered. I also provided detailed minor comments on the ms pdf (uploaded as an attachment) that should be considered during revisions. We found the pdf attachment called "PCOMPBIOL-D-22-01462_rev_v2_response to reviewer3.pdf" with 86 comments. We responded to each comment in the pdf and used tracked changes in the Word document. Thanks. Assuming that these comments can be reasonably addressed, the manuscript will provide an interesting example of a novel application of an existing quantitative method to address questions in aquatic systems and more generally in ecosystem ecology. We very much thank the reviewer for the careful reading, thoughtful comments for improvements, and balance of compliment and critique. We address each comment below and in the reviewer's attachment.

General Comments

1. The authors need to clearly define how they are using "ecosystem state". In much of the paper is seems to be used to mean a collection of certain conditions in the ecosystem. I think that is reasonable. We agree that terminology should be clearly defined, so we added a new Introduction paragraph (starting on line 81) that defines ecosystem state, state variables, and cites seminal work on this big topic. Thank you! In other places it is used interchangeably with "alternate regimes", and in a few places the term "alternate regimes" is used. I don't think it is correct to refer to regular, annual seasonal changes as "alternate regimes" as the term is usually used. Providing a clear statement of how "ecosystem state" is being used in this ms and a citation or two in support of that use would clear this up. We agree that the use of 'alternative regime' is inappropriate for a seasonal transition and do not use that here. We note the temporal correlation function found state changes related to season and stochastic events, but not 'abrupt regime shifts.' Now, we only refer to alternative regimes once in the Discussion section based on the terminology used by Bouska et al. 2020 to conceptualize the two ecosystem states we presented.

2. Related to comment #1, the likely scenario that all (or nearly all) of the state 1 observations occurred in winter is treated irregularly throughout the ms. In places it is explained clearly; in other places the text leaves open the possibility that these observations could represent a regime shift similar to those seen during the growing season in other shallow aquatic systems. For example, on line 252, the text suggests that the clear vs turbid states detected by TDA are the typical clear/turbid states observed in shallow aquatic ecosystems. We understand the concern, and rid of any conflation between the State 1 found in winters with the 'clear-water state' as defined for other ecosystems (e.g., Scheffer 2004, Larson et al. 2020 in reference list). We reframed results to be clear that State 1 was usually found in winter

and almost never in the summer plant-growing season. We added phrases in the results to emphasize State 1 was in winter, and water quality improvements were found at that time: "The increasing correlation values indicated State 1 (clear-water conditions in winter) became more prevalent over time and thus water quality winter conditions improved in those two pools."

We also changed our hypotheses and description from a 'clear-water state' to 'status-quo state', because a clear-water state typically found in shallow lakes is unlikely to be observed in this (naturally and human-impacted) turbid river. Rather, we expected 'a typical state' (which we found as States 1 and 2) and a truly 'turbid-state' (which we found as States 3-5). Thank you!

3. Visually it seems like a substantial portion of state 2 could be considered part of the tail rather than the body. How is the tail/body border determined? This is especially relevant regarding interpretation of data from the Open River study area. Good question. The TDA Mapper algorithm discovered two states, graphed as a 'body' and a 'tail.' The topological states algorithm employs a density assignment of states, which found 5 states (Figure 5). It's typical that states in the density algorithm can overlap in the body and tail of TDA Mapper (e.g., Chang et al. 2020) because they are different algorithms for calculation. Open River is typically in State 2, which occurs in the body and the tail. About 15% of the time Open River is within States 3-5, or the extreme tip of the tail. A related question: Does requiring the parameter specifications to produce a result where at least 90% of the data must be in the main body have the potential to affect the # of ecosystem states that are detected? If so, should this be considered in the interpretation of your results? Good question. No, the number of states are not affected by the data requirement. We think there is miscommunication on the 90% data requirement, which is for the entire TDA Mapper and not just the 'main body' of the TDA Mapper. We revised and expanded to read on lines 567-569: "As such, we used the following heuristics to determine the optimal cube size and percent overlap parameters like Chang et al. (2020): each node must contain no more than 10% of the data, and at least 90% of the data must be used in the main TDA Mapper structure that includes the body, tail, and outliers. In other words, at least 90% of the data must be used to create the TDA Mapper and up to 10% of data could be acceptably lost as 'noise.' We quantified and reported in the results the amount of data expressed in the final TDA Mapper output." In Results line 149, we showed: "The TDA Mapper graph output had six connected components that included more than 99% of the data (Figure 1)."

4. A brief description of the stratified random sampling design, the decision to collapse the data set across all years, and the implications of both would aid the reader in interpreting the results. This would also provide useful context for the text beginning line 339. We altered the description of data and the sampling design as suggested.

5. Topological figures have x, y and z(color) dimensions. Interpretation of color is explained. Is there meaning that can be inferred from x and y dimensions? For example, does the relative location within the "body" relative to the "tail" indicate anything? Also, there a many more samples than circles in the figures, how many samples does each circle represent? Good questions. We added on line 470: "The nodes are clusters of sites that are similar based on the data (and the number of sites within each node can vary), and the edges signify interactions or connections among nodes. Nodes that are farther apart in 2 dimensional space indicate the sites are quite different. Essentially, the undirected TDA Mapper creates a single shape of all the data, consisting of nodes and edges."

---

**Have the authors made all data and (if applicable) computational code underlying the findings in their manuscript fully available?**

The PLOS Data policy requires authors to make all data and code underlying the findings described in their manuscript fully available without restriction, with rare exception (please refer to the Data Availability Statement in the manuscript PDF file). The data and code should be provided as part of the manuscript or its supporting information, or deposited to a public repository. For example, in addition to summary statistics, the data points behind means, medians and variance measures should be available. If there are restrictions on publicly sharing data or code —e.g. participant privacy or use of data from a third party—those must be specified.
Reviewer #1: None
Reviewer #2: **No:** draft says: Our final datasets and analysis scripts will be archived in GitLab (DOI: XXXXXXXX) following peer review
Reviewer #3: Yes
Our complete data set was provided at time of PLOS peer review. The data and analysis scripts are undergoing peer review through U.S. Geological Survey, per our Fundamental Science Practices policies to ensure rigorous documentation. The analysis code, data, and metadata will be publicly archived in ScienceBase with a unique identifier (DOI) alongside the public release of this paper. We respect and thank PLOS for having their clear Data policy.

---

PLOS authors have the option to publish the peer review history of their article (what does this mean?). If published, this will include your full peer review and any attached files.

If you choose "no", your identity will remain anonymous but your review may still be made public.

**Do you want your identity to be public for this peer review?** For information about this choice, including consent withdrawal, please see our Privacy Policy.
Reviewer #1: No
Reviewer #2: No
Reviewer #3: **Yes:** Jeffrey N Houser

Figure Files:
While revising your submission, please upload your figure files to the Preflight Analysis and Conversion Engine (PACE) digital diagnostic tool, https://pacev2.apexcovantage.com. PACE helps ensure that figures meet PLOS requirements. To use PACE, you must first register as a user. Then, login and navigate to the UPLOAD tab, where you will find detailed instructions on how to use the tool. If you encounter any issues or have any questions when using PACE, please email us at figures@plos.org. DONE
Data Requirements:
Please note that, as a condition of publication, PLOS' data policy requires that you make available all data used to draw the conclusions outlined in your manuscript. Data must be deposited in an appropriate repository, included within the body of the manuscript, or uploaded as supporting information. This includes all numerical values that were used to generate graphs, histograms etc.. For an example in PLOS Biology see here: http://www.plosbiology.org/article/info%3Adoi%2F10.1371%2Fjournal.pbio.1001908#s5.
Reproducibility:
To enhance the reproducibility of your results, we recommend that you deposit your laboratory protocols in protocols.io, where a protocol can be assigned its own identifier (DOI) such that it can be cited independently in the future. Additionally, PLOS ONE offers an option to publish peer-reviewed

clinical study protocols. Read more information on sharing protocols at https://plos.org/protocols?utm_medium=editorial-email&utm_source=authorletters&utm_campaign=protocols

Thank you for your willingness to serve as a peer reviewer for the Upper Midwest Environmental Sciences Center.

I have attached a copy of the manuscript to this email. If you have any questions or comments feel free to contact me. A copy of the instructions for reviewers of UMESC manuscripts is attached, after my contact information.

Heather Jensen, Biologist/Data Manager
USGS-Upper Midwest Environmental Sciences Center
2630 Fanta Reed Road
La Crosse, WI 54603
hjensen@usgs.gov

U.S. GEOLOGICAL SURVEY
Upper Midwest Environmental Sciences Center
2630 Fanta Reed Road
La Crosse, Wisconsin 54603
Phone 608-783-6451, Fax 608-783-6066
Due Date: **11/11/2022**

Thank you for agreeing to review the attached manuscript for the U.S. Geological Survey's Upper Midwest Environmental Sciences Center.

To maximize the value and impact of your review, please read and follow the INSTRUCTIONS TO REVIEWERS OF SCIENTIFIC DOCUMENTS. If you wish, your identity will be withheld from the author. Please indicate this when you return your comments.

Please complete the information at the bottom of this letter and return it along with your completed review by **11/11/22**. If you cannot complete your review within the allotted time frame, please let me know as soon as possible.

Once again, thank you for consenting to serve as a reviewer for this manuscript.

"Quantifying ecosystem states and state transitions of the Upper Mississippi River using topological data analysis," by Larson, D.M., Bungula, W., McKean, C., Stockdill, A., Lee, A., Miller, F., and Davis, K.

Sincerely,
Heather Jensen

Name of Reviewer: Kathi Jo Jankowski        Date of review 12/8/22

Area(s) of expertise: freshwater ecology

INSTRUCTIONS TO REVIEWERS OF SCIENTIFIC DOCUMENTS

Your responsibility as a reviewer for the Upper Midwest Environmental Sciences Center is to help us to improve the quality of our science by providing constructive, thoughtful, but forthright comments on the degree to which the attached document meets current standards recognized by the scientific community, and the degree to which the work is timely and relevant.

Please read the document carefully and evaluate it objectively. Restrict your technical comments to your area of expertise, but feel free to render opinions or raise questions about larger scientific issues that may be relevant. To the extent possible, justify your comments with supporting evidence just as you would do when presenting your own scientific work. Please do not refrain from offering relevant opinions, but also label them as such. Test your comments for fairness, objectivity and tone of delivery by asking yourself if you would be comfortable presenting your comments, face-to-face, to the author and a panel of your peers.

Do not consider prevailing opinion infallible and risk rejecting an important scientific document only because its method or conclusions are different from current orthodoxies. Creativity and innovation are important, and must be valued and weighed commensurately. On the other hand, do not be misled by persuasive writing if the document shows inadequate data or deficient analyses.

You may add your comments directly to the document, but also summarize and explain the important ones in the comment section of this form. Indicate which comments are mandatory and must be addressed in subsequent drafts.

This document is the intellectual product of the author and property of the U.S. Government. It is being distributed solely for purposes of scientific peer review. The document's contents are deliberative and predecisional, so they must not be disclosed or released by the product's reviewers. This document is still undergoing review for release by the U.S. Geological Survey (USGS). Until this review is complete, this document does not represent official USGS findings or policy.

Please address each of the following questions as you review the enclosed manuscript:

1. Is the objective of the study scientifically relevant and clearly stated? Yes

2. Are the study design and methods appropriate for the purposes of the study? Are the methods presented in sufficient detail to enable the reader to repeat the study?

   The authors use a novel technique that hasn't frequently been used to evaluate ecological data to address some interesting ecological questions. I do have some concerns about the clarity of their presentation, however, that are reflected in my comments in the text. I think if this is aimed at an ecological audience (rather than a statistical or modeling audience), there will need to more clarity in linking some of the methodological details to ecological concepts – for example, how does a topological state relate to an ecological

state?  What is a “state variable” in context of the analysis vs ecological state theory? Some clarity in those points and others in the methods description would be helpful.  We fully agree and are targeting an ecological audience (target journal: PLOS Computational Biology). We have carefully revised the Introduction and Methods sections to define the jargon like ecosystem states and state variables, and improve linkages to the methods.

3. Are there demonstrable errors of fact or interpretation?

   No

4. If your expertise includes statistics, do you find that the statistical methods are applied appropriately and interpreted accurately?  If your expertise does not include statistics, do the analyses convince you that the conclusions are correct?

   The methods seem robust, although this is certainly not a familiar method to me, so I cannot judge any of the decisions that were made regarding algorithm design, parameter selection, etc. Thank you. Author Dr. Wako Bungula is an expert in TDA, which was a great source of learning and confidence in our approaches.

5. Is the study innovative?

   Yes, it’s a neat application of a new statistical technique to these data that reveals some interesting patterns. Cool, thank you!

6. Is the writing clear, concise, and unambiguous?  If not, suggest by example how the document could be improved.

   Yes.

7. Has pertinent literature been used effectively and convincingly?   If not, provide specific recommendations.

   It would be helpful to relate some of the findings to existing knowledge of gradients in water quality parameters in the UMR as well as other large rivers. In the Discussion, we add 2 new paragraphs of how these results relate to other known aquatic systems (like shallow lakes) and other rivers worldwide. Thanks for this idea.

MANUSCRIPT REVIEW

REVIEW COMMENTS

General Comments:

This is an interesting and novel approach to understanding spatial and temporal variation in water quality in a large river and the paper is well-written. The approach facilitates a multivariate understanding of how conditions have changed and highlight relationships across areas and variables that are hard to elucidate using other methods. I commend the authors on applying a complex technique to this dataset to address interesting questions.

However, I have three main suggestions.

First, I think there should be a clearer link between the methods you use and ecological state theory if you're going to make the case that this method allows you to quantify ecological states and state transitions. In my understanding, there are pretty clear definitions of what those are in the literature (and people get very particular about them!), so I think you should clearly map things like "topological state" to "ecological state". Are they the same thing? Good point. We added new paragraphs to the Introduction with seminal citations to the terminology, and made better links between ecosystem state concepts and the new methods we used. We do suggest that topological state and ecological state are the same.

Second, I think in general more clarity on the methods would be helpful. Most likely people will not be familiar with this approach, so more clear wording and explanation of what things are and why you do them would be helpful for a naïve reader. What is a node? An edge? What do they mean? We substantially revised the Methods section based on your comment. Defining the node and edge is important, and we include that in the figure legends as well.

Finally, depending on whether your focus is on the method or the ecological results, you may want to shift the focus of the discussion a bit. For example, if the latter, I think there could be further exploration of the actual results in the discussion. What were the seasonal patterns – are winters shifting more than summers? Or further comparisons of your outputs to other existing studies that describe the gradients in these variables across the river ecosystem (e.g., Houser et al. 2010) – what does this approach tell us that previous work doesn't? A lot of the discussion is focused on how this is a novel technique that can inform restoration and management, which is potentially true, but I think can be shortened, especially depending on the particular journal you target. Thank you. We heavily revised the discussion that includes how the method revealed state transitions and applicability of results to other rivers. We emphasized what new information about this river was provided, as well as transferability of the results to other ecosystems.

<u>Specific Comments:</u>

Most of my comments are in the text, but two additional questions –

Do you evaluate differing numbers of variables needed to classify states more or less effectively? We modeled all 8 variables in TDA simultaneously, but the Figure 2 graphs and diagnostics helped to determine which variables were state variables. We did not try running TDA Mapper with less variables (backward variable selection approach) because TDA Mapper chose to use 99% of the data, which signals all variables are somewhat important in determining the 'shape' of water quality.

If others are going to apply this technique, do they need as much data as LTRM has?

This is a great question, given LTRM has exceptional ecosystem data and we discuss 'transferability of the TDA approach.' We add an entirely new paragraph based on this question, starting on line 365: "Ecologists have emphasized the need to apply ecosystem states concepts to rivers worldwide, while acknowledging most rivers do not have sufficient data to properly evaluate states (2,7,18,23). The Mississippi River has exceptional long-term data, and our results supported the concepts of alternative states in this particular river (18). We suspect that similar ecosystem states based on water quality state variables can be found in other rivers, although we are unaware of other case studies to-date other than 'clear-water' and 'turbid-states' based on aquatic plant abundance and water quality state variables (23). The Regime Shifts Database (7) could serve as a data source to test our TDA ecosystem states framework in other rivers with similar water quality data (e.g., known hypoxia regime shifts within the Amite River, Brazo River, Chester River, and Indian River in the USA). For ecosystems without the big data like the Mississippi River, monitoring system status and trends (like state transition warnings) could focus on the three state variables in aquatic systems: total phosphorus, chlorophyll a, and suspended solids. Transferability of the TDA approach (outlined in Figure 5) will be limited to ecosystems with sufficient data, although the number of sites and variables required for useful ecological assessments with TDA are not yet known."

Manuscript

Click here to access/download;Manuscript;Larson TDA river PLOS 15March2023 allrevisions_clean.docx

# Title: Quantifying ecosystem states and state transitions of the Upper Mississippi River System using topological data analysis

**Authors:** Danelle M. Larson[1‡],Wako Bungula[2], Casey McKean[3], Alaina Stockdill[3], Amber Lee[3], Frederick Miller[3], Killian Davis[3]

**Affiliations:**
[1] U.S. Geological Survey, 2630 Fanta Reed Road, La Crosse, WI 54603; dmlarson@usgs.gov; no fax; ORCID: 0000-0001-6349-6267; [‡] Corresponding Author: dmlarson@usgs.gov
[2] University of Wisconsin La-Crosse, Department of Mathematics and Statistics, 1027 Cowley Hall, La Crosse, WI 54603
[3] University of Wisconsin La-Crosse, Research Experience for Undergraduates Program, 1027 Cowley Hall, La Crosse, WI 54603

**Corresponding author:** Danelle M. Larson, dmlarson@usgs.gov

**Running title:** quantifying riverine states via topology

# ABSTRACT

Aquatic systems worldwide can exist in multiple ecosystem states (i.e., a recurring collection of biological and chemical attributes), and effectively characterizing multidimensional states will aid protection of desirable states and guide restoration. The Upper Mississippi River System is a large, floodplain river spanning 2200 km with a 30-year water quality monitoring data set and and ecosystem states need depicting that can guide river restoration. We coupled this highly dimensional dataset with multiple topological data analysis (TDA) techniques to classify ecosystem states, identify state variables, and detect state transitions over 30 years. Across the entire system, TDA identified five ecosystem states. State 1 was characterized by exceptionally clear, clean, and cold-water conditions typical of winter (i.e., a 'clear-water state'); State 2 had the greatest range of environmental conditions (i.e., a 'status-quo state'); and States 3, 4, and 5 had extremely high concentrations of suspended solids (i.e., 'turbid-states', with State 5 as the most turbid). The TDA mapped clear patterns of the ecosystem states across several riverine navigation reaches and seasons that furthered ecological understanding. State variables were identified as suspended solids, chlorophyll *a*, and total phosphorus, which are also state variables of shallow lakes worldwide. The TDA change detection function showed short-term state transitions based on seasonality and episodic events, and also provided evidence of gradual, long-term changes due to water quality improvements over three decades. These results can inform decision making and guide actions for regulatory and restoration agencies by assessing the status and trends of this important river and provide quantitative targets for state variables. The TDA change detection function may serve as a new tool for predicting the vulnerability to undesirable state transitions in this system and other ecosystems with sufficient data. Coupling ecosystem state concepts and TDA tools can be transferred to any ecosystem with large data to help classify states and understand their vulnerability to state transitions.

**Keywords:** alternative stable ecological state, ecological regime shift, ecosystem state change, topology, topological state, water quality, vulnerability

## AUTHOR SUMMARY

The Mississippi River in the USA is a large, complex ecosystem that had many changes to water quality since the 1990s. We used a new set of mathematical tools called 'topological data analysis' to help identify common water quality conditions or 'states' of this important river. We also explored when and why the states have changed over the past 25 years. The tools identified five common water quality states, distinguished by nutrients and clear waters or muddy waters. State changes were commonly detected as seasonal transitions in the higher latitude reaches. The state changes also showed that water quality had improved or declined gradually over many years, depending on the river reach. Reaches named Pool 4 and Pool 8 had become clearer over time. Pool 13 became muddier over time with several undesirable state changes. Three other river reaches were consistently muddy. Our study is a good example of how these topological data analysis tools may help others managing ecosystems by improving communications about what states exist and which are desirable. Using these tools with long-term data can also alert ecosystem managers to the ongoing, complex changes and vulnerability to undesirable changes so they may intervene with restoration.

## Author contributions:

Larson and Bungula contributed to conceptualization, funding acquisition, formal analysis, data curation, supervision, and writing-original draft preparation. All authors contributed to data curation, investigation, visualization, and writing-review and editing.

**Data availability statement:**

All data were publicly accessed on 1 June, 2021 through the Upper Mississippi River Restoration Program's *Long-term Resource Monitoring* (https://umesc.usgs.gov/ltrm-home.html). Our final datasets and analysis scripts are publicly and permanently archived in ScienceBase (DOI: 10.5066/P976O6PP).

## INTRODUCTION

Many ecosystems worldwide are undergoing severe and rapid changes and managing ecosystem states and states transitions are a time-sensitive and difficult challenge. Large floodplain rivers are especially degraded and continue to experience major changes to hydrology and water quality (1,2). Monitoring ecosystem states and states transitions with long term data and advanced analytical tools is critical for protecting and restoring ecosystems that humanity values or depends on (3).

Ecosystem states, or alternative states, are a recurring collection of biological and chemical attributes that arise from structuring processes and have some resilience to changes within its state space (4–6). Characterizing and defining the state is always multidimensional, but typically only a few state variables can properly characterize the ecosystem state. State variables are features of the biological community and physical environment that characterize the structure and functions of an ecosystem (6). State transitions are either temporary or permanent changes to state variables that alter the ecosystem state. In contrast, 'regime shifts' are state transitions that are substantial, persistent through time, and difficult to reverse with restoration (7). Identifying ecosystem states and the associated state variables is essential for describing the ecosystem and setting clear and reasonable ecological restoration targets.

Ecosystem states are well defined in many types of ecological systems, but not for large rivers. Many ecosystems have at least two alternative states often defined by the lower trophic levels, such as the plant-dominated or phytoplankton-dominated states in lakes (8,9), marine kelp forest or kelp-deforestation (10), mesic grasslands or desertification (11), mesic grasslands or shrublands (12), and

diverse coral reefs or macroalgal-dominated reefs (13). A few examples of state transitions include the restoration of aquatic vegetation and water quality in shallow lakes (9), altered disturbance regimes and subsequent restorations in tallgrass prairies (14,15), and successive state shifts and a novel state arising from multiple stressors in a deep lake (16).

Ecosystem states have been conceptualized (17,18) but not well tested in rivers until recently (19–22). The lack of research may be attributed to the difficulty in defining a riverine states because the multivariate methodology to tackle a state definition is complex and data is often sparse. The global scientific and management community calls for applying an ecosystem states framework to rivers, while acknowledging most rivers do not have sufficient data to properly evaluate states (7,18,23). When sufficient data are available, identifying the states and drivers of the transition between states is key for ecological restoration planning, post-project monitoring, and adaptive management (6).

State concepts are complex and multidimensional, and therefore requires substantial data, advanced and novel analysis techniques for disentangling patterns, and expertise for its ecological interpretations. More analytical tools for 'big data' that can reasonably predict ecosystem states and impending state transitions will help conserve, restore, and manage ecosystem resilience (3). Topological data analysis (TDA) provides a powerful suite of tools for disentangling patterns and relationships among multidimensional data and presenting data visually (24). Tools of TDA effectively shown changes in the human gut following bacterial disturbance (25), predicted wildfire severity (26), and assessed vulnerability of residential homes to climatic changes (27). The use of TDA remains rare in ecology, although TDA may be an ideal tool for many ecological applications because of its multidimensional framework (28).

**Objectives and Hypotheses:**

We provided an analysis of ecosystem states and transitions using topological data analysis (TDA) tools to better characterize the Upper Mississippi River System, which is a significant ecosystemto humans and wildlife (2). We hypothesized that this river would be in at least two states (18,23): a 'statusstatus-quo state' that is common and characterized by aa broad range of turbidity and nutrient concentrations, and a 'turbid-state' characterized by extremely high turbidity due to high suspended solids and chlorophyll *a* (Hypothesis 1). We expected at least three state variables similar to regional shallow lake ecosystems (9) that include total phosphorus, suspended solids, and chlorophyll *a* (Hypothesis 2). We hypothesized that different riverine were in different states (Hypothesis 3) given their differing water quality conditions and restoration priorities (2,29). We hypothesized TDA would detect an abrupt state transition around years 20072007–2010 when aquatic vegetation rebounded and stabilized at high prevalence in several upper reaches and water quality conditions improved (19,30) (Hypothesis 4a). We further expected that TDA could also detect gradual water quality improvements based on the proportion of sites leaving the 'turbid-state' and entering a 'status-quo state' (Hypothesis 4b).

## RESULTS

### *How many ecosystem states were detected in the UMRS? Hypothesis 1*

The TDA Mapper inputted 8 water quality variables collected from 69,307 sites over 27 years onto a single TDA structure (Figure 1). The TDA Mapper graph output had six connected components that included more than 99% of the data. Only <1% of the data points were excluded from the main TDA structure as 'outliers' or 'noise' (Figure 1 dark violet nodes). The main TDA structure had a shape of a "body" (containing ~90% of the data) and a "tail" (containing about 10% of the data), suggesting there were two main ecosystem states (in support of Hypothesis 1).

**Figure 1.** Graphical output from topological data analysis (TDA Mapper) coupled with a density-based state assignment of the topological and ecosystem states for the Upper Mississippi River

System, USA. Data inputs included 8 water quality variables collected four times each year from 1993–2020 at a total of 69,307 sampling sites. The graph is comprised of nodes (circles), which cluster sampling sites along the river based on water quality similarities, and the edges (lines) show statistical connections among nodes. An undirected graph (visualized by ignoring color scheme and focusing only on data shape) showed two topological states, indicated by the tail being less connected to the main body and thus having differing topology. The TDA Mapper was then transformed into a directed TDA Mapper graph using the topological states algorithm that further refined the shape into five states (color coded). The topological states may be conceptualized as ecosystem states, especially when further exploring data within the nodes (circles) to understand possible ecosystem state variables that classified each site within a node and distinguish among topological states. , The dark purple nodes not connected to the main structure (<1% of sites) were outliers and not classified within a state.

The topological states algorithm further refined the two ecosystem states from the TDA Mapper outputs into five ecosystem states in a directed graph (Figure 11).States 1 and 2 were nested within the TDA Mapper body and States 3–5 were nested within the TDA Mapper tail. There were five, small transition nodes (meaning the sites were classified into both states simultaneously), which lied between states 1 and 2 but couldn't be seen graphically in 2 dimensions because the nodes were small. Transition nodes were visible in a 3-dimensional TDA Mapper output, but we did not explore the transition nodes further due to rarity of transition nodes. States 1, 2, 3, 4, 5 contained 20%, 85%, 2%, <1%, and <1% of data across the six reaches and 27-year record, respectively.

*What were the state variables that defined and distinguished riverine ecosystem states? Hypothesis 2*

State 1 may be considered a 'clear-water state' because this state had the lowest total phosphorus, highest dissolved oxygen, lowest chlorophyll *a*, coldest water temperatures, slowest water velocity, and lowest suspended solids relative to the other four states (Figure 2). State 1 was differentiated from State 2 principally by lower suspended solids (Figures 2f, 2h). State 2 contained most sites (Table 1; the so-called 'status-quo' state), and the state variables differentiating State 2 from others

included total phosphorus, chlorophyll *a*, and suspended solids (in support of Hypothesis 2; Figure 2). State 2 had the greatest variability and many statistical outliers for all state variables. State 2 typically had lower nutrients but higher chlorophyll *a* relative to States 3, 4, and 5.

States 3, 4, 5 within the structure's tail resembled the 'turbid-state.' These three states were like each other for seven water quality variables (Figure 2a–2g) and differentiated from one another by the relative concentrations of suspended solids (Figure 2h). Compared to States 1 and 2, the States 3–5 had greater total phosphorus, lower dissolved oxygen, greater water depths and velocities, and orders of magnitude greater suspended solids. States 3, 4, and 5 also had higher water depths and velocity compared to States 1 and 2. Total nitrogen was the only variable that didn't vary in data distribution among the five state classifications (Figure 2a), so we determined total nitrogen was not a state variable.

**Table 1.** The distribution of sites in each upper Mississippi River reach (column 1: Pool name, field station city and state) that were classified into a topological/ecosystem state within years 1993–2019. Table 1a shows the percentage (%) of sites in each state according to reach for comparisons of ecosystem states *among the six*reaches (i.e., columns sum to 100%). Table 1b shows the percentage (%) of sites classified within a reach to compare the distribution of states *within each* reach. Sites sometimes can exist in more than one node, and therefore the percentage of sites within a state may sum to greater than 100% (i.e., rows in Table 1b sum greater than or equal to 100%).

**TABLE 1a**

| **River Reach** | **State 1** | **State 2** | **State 3** | **State 4** | **State 5** |
|---|---|---|---|---|---|
| **Pool 4 (Lake City, MN)** | 31 | 16 | 0 | 0 | 0 |
| **Pool 8 (Onalaska, WI)** | 35 | 17 | <1 | 0 | 0 |
| **Pool 13 (Bellevue, IA)** | 26 | 18 | <1 | 0 | 0 |
| **Pool 26 (Brighton, IL)** | 5 | 16 | 8 | 5 | 6 |

| | | | | | |
|---|---|---|---|---|---|
| **Open River (Jackson, MO)** | <1 | 17 | 90 | 95 | 93 |
| **La Grange (Havana, IL)** | 2 | 16 | <1 | 0 | <1 |

**TABLE 1b**

| **River Reach** | **State 1** | **State 2** | **State 3** | **State 4** | **State 5** |
|---|---|---|---|---|---|
| **Pool 4 (Lake City, MN)** | 37 | 81 | 0 | 0 | 0 |
| **Pool 8 (Onalaska, WI)** | 39 | 78 | <1 | 0 | 0 |
| **Pool 13 (Bellevue, IA)** | 27 | 82 | <1 | 0 | 0 |
| **Pool 26 (Brighton, IL)** | 7 | 95 | <1 | <1 | <1 |
| **Open River (Jackson, MO)** | <1 | 87 | 9 | 3 | 1 |
| **La Grange (Havana, IL)** | 5 | 92 | <1 | 0 | <1 |

**Figure 2.** Graphical outputs from topological data analysis (TDA Mapper) showing ecological gradients of 8 water quality variables in the Upper Mississippi River System, USA (*n*=69,307 sampling sites from 1993–2020). The color coding represents relative values for each variable; dark violet colors are relatively low values, whereas greens and yellows are relatively high values. The boxplots below each TDA Mapper graph showed the data distributions for each ecosystem state (that corresponds to the states in Figure 1). Boxplots are standardized by boxes (containing the 25$^{th}$, 50$^{th}$ (median), and 75$^{th}$ percentiles), the whiskers (containing the 25$^{th}$ and75$^{th}$ percentiles), and the dots (statistical outliers). The x-axis on the boxplots represents the five topological and possibly ecosystem states (States 1–5). The y-axis represents variable units (total nitrogen = mg $L^{-1}$; total phosphorus = mg $L^{-1}$; dissolved oxygen = mg $L^{-1}$; chlorophyll *a* = µg $L^{-1}$; water depth = cm; temperature = °C; velocity = m $sec^{-1}$; suspended solids = mg $L^{-1}$).

### *Did reaches exist in different ecosystem states? Hypothesis 3*

The six study reaches spanning 2200 km of river were classified into different topological and ecosystem states (in support of Hypothesis 3). Different states were highlighted in different locations on the main TDA structure (Figure 3) and noted by the percentage of sites in each reach's state classification (Table 1). Pool 4 (Minnesota), Pool 8 (Wisconsin), Pool 13 (Iowa), and La Grange Pool (Havana, Illinois; Illinois River) were within States 1 and 2 across the 27-year record. Therefore, Pools 4, 8, and 13 were principally categorized as within a 'status-quo state' (State 2) characterized as having a broad range of ecological conditions but often included the 'clear-water' state (27–39% sites within State 1 for these reaches). Pool 26 (Brighton, Illinois) existed in all five states, but State 2 was dominant (Table 1, Figure 3). Open River (Jackson, Missouri) was the principal reach within States 3-5. Open River was rarely in State 1, mostly within state 2, and 13% of sites were within the turbid-states of States 3–5.

**Figure 3.** Topological data analysis (TDA) showed spatial differences of water quality (8 variables; total nitrogen, total phosphorus, dissolved oxygen, chlorophyll *a*, water depth, water temperature, velocity, and suspended solids) among six reaches on the Upper Mississippi River System (years 1993–2020). The green colors highlighted where sampling sites within each reach grouped onto the primary TDA Mapper structure, and the differences indicated spatial differences among the six reaches. Dark green nodes had more sites within each node compared to the lighter green nodes. Reaches can be classified into specific ecosystem states by referencing their positions within Figure 11; for example, Pool 4 was within States 1 and 2 because the green highlights are on the left side of the TDA structure (panel a). As another

example, Open River was principally within State 2–5 because the green highlights are on the right side of the TDA structure and its tail (panel f). Reaches can also be characterized by water quality state variables in Figure 22; for example, Open River is typically characterized by high velocity and suspended solids.

*Did reaches undergo state transitions within the past 30 years? Hypothesis 4*

The six reaches varied substantially in their ecosystem state stability and the frequency of state transitions (Figure 4). Pool 26 and La Grange were stable within State 2 and occasionally State 1 and did not undergo state transitions. Pools 4, 8 and 13 displayed cyclic dynamics between State 1 and State 2 that were recurring annually due to seasonal changes in water quality. State 1 dominated in winter of most years (S1 Figure) and State 2 dominated in the other three seasons (S1 Figure). State 1 was uncommon but possible outside of winter, and State 1 was rare in summer (indicated by low proportion values of State 1 during summers). State 1 was seasonally dynamic as indicated by cyclic temporal proportion values that were greatest in winter, declined by spring and summer, and then increased by the following autumn and winter (Figure 4).

**Figure 4.** Change detection plots showed the transitions in ecosystem states within six reaches of the Upper Mississippi River System from years 1993–2020. The proportion value (y axes) is calculated as the proportion of sites classified within each state each season. Occasionally, sites were classified into multiple states, and therefore the proportion sum may exceed 1.0. Points that fall directly on the year were collected in winter, followed by spring, summer, and autumn samples. Data from 2003 was missing due to logistical constraints for collection.

**S1 Figure**. Graphical output from topological data analysis (TDA Mapper) showing a strong seasonal component to the water quality data for the Upper Mississippi River System, USA. Data inputs included 8 water quality variables collected four times each year from 1993–2020 at a total of 69,307 sampling sites. The graph is comprised of nodes (circles), which cluster sampling sites along the river based on water quality similarities, and the edges (lines) show statistical connections among nodes. We highlighted in color the seasons in which sites were sampled. Seasonality can be connected to ecosystem states by referencing Figure 1 positions; for example, winter is typically characterized by State 1.

Pool 4 and Pool 8 gradually shifted towards State 1 through time. The change detection function did not detect an abrupt state transition around years 2008–2010 in Pools 4 and 8 (refuting Hypothesis 4a). However, Pool 4 and Pool 8 had increasing minimum proportions values through time for State 1, especially after year 2010 (Figure 4). The increasing proportions values indicated State 1 (a 'clear-water' state in winter) became more prevalent over time and thus water clarity during winters improved in those two reaches since 1993 and especially since 2010 (in support of Hypothesis 4b).

Pool 13 showed different patterns in state transitions (Figure 4). The increased minimum proportion values over time in State 1 were less pronounced than in Pools 4 and 8, indicating less increase in water clarity from reduced suspended solids. Since 2015 in Pool 13, the previous pattern of transitioning to State 1 in winter had become less common and predictable compared to previous years. State 3 (a turbid-state) was first entered in 2017 and again in 2019 at some Pool 13 sites, indicating decreased water clarity from increased suspended solids.

Open River (Jackson, Missouri) transitioned between State 2 and State 3 sporadically four times over 27 years. Transitions to State 3 generally only lasted one or two seasons before returning to State 2. The four state transitions in Open River reach occurred across various seasons and years (specifically, spring-summer 1999; winter-spring 2010; winter 2014; autumn 2015).

## DISCUSSION

This is the first study to use powerful topology tools of this type to quantify ecosystem states and state transitions in a large ecosystem. Our topological analyses successfully detected patterns for multiple riverine states, the defining state variables, and differences among reaches in the Upper Mississippi River System (in support of our Hypotheses 1–3 and visualized in Figures 1–4). Our results expand the existing Mississippi River literature by using a multivariate approach to classify nearly 70,000

sites into multiple ecosystem states, identify water quality state variables, and explore state transitions. The TDA change detection analysis examined multivariate state transitions (Figure 4 and temporal proportion values), which is a powerful approach to thinking about multiple correlated variables and how they vary synchronously in space and time. The TDA detected short-term state changes based on seasonality and episodic events and provided evidence of gradual, long-term changes due to water quality improvements over three decades (Figure 4). We discuss how TDA may be used as a new technique for describing ecosystem states at multiple spatiotemporal scales, how the results will inform ecological restoration and management, and how the state concepts tested with TDA methods may be transferred to other ecosystems that exists in one or more states.

### *TDA as a novel technique for ecosystem states and transitions*

Testing ecosystem state concepts with TDA were inspired by past research of the human-gut microbiome (25,31), and our study shows TDA can also effectively identify ecosystem states and state variables from big data of large ecosystems. Our TDA results aligns well with previous studies of Upper Mississippi River System that described sharp differences among reaches and water quality changes through time (2,19). The TDA results supported another author's previous hypothesis that the Upper Mississippi River System exists in two alternative states: status-quo (States 1 and 2) and turbid-states (States 3–5) as defined by state variables of suspended solids and chlorophyll *a* (18). State 2 had very high variability across all state variables that defines the status-quo state, meaning the water quality is highly variable across large spatiotemporal scales. Likewise, the main differentiation between the rare States 3–5 were based solely on suspended solid concentrations, which may be better represented as an ecological gradient than firm state classification via TDA. Although we could have altered the TDA density algorithm to explore more sub-states within these five states, we didn't have justification for refinement and wanted to provide a reasonable number of states that were clearly differentiated for aiding applied conservation (29).

The ecosystem states concepts, the TDA methods chosen to reflect ecosystem states, and the TDA results require some subjectivity in determining usefulness towards ecological understanding and management. For example, we suggested the TDA Mapper outputs of two alternative states (Figure 1) and the TDA algorithm refining two states into five states (Figure 2) are both valuable to display and guide resource management across the Upper Mississippi River System. Users must decide at which timescale a state is 'stable.' The change detection function we used statistically determined when states had transitioned, but the user must determine that those results signify expected seasonal transitions (Figure 4, S1 Figure), 'abrupt regime shifts' (not detected in this study), or unmeaningful noise.

The TDA change detection function explored whether state transitions were dramatic and long-standing (i.e., an abrupt regime shift) or whether transitions were temporary state changes (i.e., transient dynamics) (Hypothesis 4). The TDA detected frequent transient dynamics based on seasonality and episodic state changes (Figure 4) but did not detect abrupt state changes with sharp break points in time. Future TDA analyses could include data on submersed aquatic plant biomass or species composition (32) because aquatic plants are likely another state variable (18) that may reveal abrupt shifts around 2010 in support of our Hypothesis 4. Other TDA tools like 'persistence homology' can detect specific change points (33), and thus may detect abrupt state transitions that are hypothesized to occur in rivers with long retention times (34) or following ecological restoration (18). Alternatively, the river's ecology may be highly multidimensional and complex so that there was not a single point in time where major and abrupt changes happened, but rather changes were gradual over decades and most accurately portrayed with the change detection function used herein (Figure 4). The lack of abrupt regime shifts in the Upper Mississippi River System would be different than is commonly observed within nearby shallow lake systems, which can transition to the alternative state within a few days and persist for years (8,9).

The TDA allows extraction of information at finer spatiotemporal scales but was beyond the scope of this paper but could be explored in future work. For example, information from the data within the nodes can further describe how specific sites, seasons (S1 Figure), riverine habitat strata, and years are classified within each state to further ecological understanding and prioritize ecological restorations. Sites classified within multiple states, transition nodes, or the TDA outliers could be further explored for unusual site characteristics and assessing site-level variability that affects ecosystem state.

### *Ecosystem states results can guide ecological management & restoration*

Coupling ecosystem state theories with scientific models can provide urgently needed guidance for ecological restoration and management (35,36). Previously applied uses of state theory included predicting pending state transitions (37–39), managing systems for resilience and preventing undesirable transitions (38), and managing state variables to restore or protect resilience of desirable ecosystem states (9,40). However, state classification and state transition models are rarely used in large monitoring programs because of modeling complexities and the inability of institutions to practically apply model outputs (3).

We used big data (nearly 70,000 sites and 8 variables) from long-term river monitoring (41), and these TDA results can guide structured decision making and vulnerability assessments in the Upper Mississippi River System. This is the first study in this large watershed to classify ecosystem states, which is foundational for describing the system's ecological structures and functions. Having defined ecosystem states allows practitioners to communicate which states are desirable for preservation and which are undesirable for mitigation, as initiated in (18,29,42). We identified the state variables (specifically total phosphorus, suspended solids, and chlorophyll *a*), which can guide decision making and actions for regulatory and restoration agencies by providing quantitative targets. Currently, specific regional water quality criteria are recommended for total phosphorus and chlorophyll *a*, but not

suspended solids (43). Specified targets for suspended solids concentrations are lacking, although water clarity remains a common restoration goal (29), and our TDA results echo the importance of suspended solids a state variable.

The TDA's change detection can be a novel tool for assessing state transitions and vulnerability as new, long-term data are gathered. This function effectively signaled state changes in the Upper Mississippi River System due to seasonality, episodic events, and gradual changes to water quality (Figure 4). The state transition modeling in this system can continue to reveal gradual improvements to water quality and signal vulnerability to undesirable state transitions if States 3–5 become more prominent in certain reaches, like Pool 13 (Figure 4). The TDA tool detected changes to the variability of the number of sites within a state as the proportion values, and a change in time series variability can be an early warning indicator of ecological transitions (44). The TDA could detect if more sites within Pool 13 continue to enter State 3 more frequently or persistently in the near future. The state variables of total phosphorus, chlorophyll *a*, and suspended solids identified for the Mississippi River are the same as those of lake ecosystems (8), and so assessing vulnerability to state transitions using these variables could be a warning in other aquatic systems beyond the Mississippi River. Lastly, the states could become a categorical response variable in future models that aim to assess the environmental drivers and threshold responses, although researchers should carefully consider whether such information is better addressed with gradient analyses or state classification (45).

### *Transferability of TDA to other ecosystems*

Ecologists have emphasized the need to apply ecosystem states concepts to rivers worldwide, while acknowledging most rivers do not have sufficient data to properly evaluate states (2,7,18,23). The Mississippi River has exceptional long-term data, and our results supported the concepts of alternative states in this particular river (18). We suspect that similar ecosystem states based on water quality state

variables can be found in other rivers, although we are unaware of other case studies to-date other than 'clear-water' and 'turbid-states' based on aquatic plant abundance and water quality state variables (23). The Regime Shifts Database (7) could serve as a data source to test our TDA ecosystem states framework in other rivers with similar water quality data (e.g., known hypoxia regime shifts within the Amite River, Brazo River, Chester River, and Indian River in the USA). For ecosystems without the big data like the Mississippi River, monitoring system status and trends (like state transition warnings) could focus on the three state variables in aquatic systems: total phosphorus, chlorophyll a, and suspended solids. Transferability of the TDA approach (outlined in Figure 5) will be limited to ecosystems with sufficient data, although the number of sites and variables required for useful ecological assessments with TDA are not yet known.

Overall, TDA was a novel technique that effectively addressed many hypotheses regarding the Upper Mississippi River System as characterized by predictable ecosystem states, a few state variables, and state transitions. The TDA may provide an early warning indication of vulnerability to undesirable ecosystem transitions, such as Pool 13's increasingly frequent transitions to the undesirable state 3 with high suspended sediment loading. Testing ecosystem states in freshwater systems (beyond well-studied shallow lakes) is important, while being cognizant to the limitations and misuses of the theories (45). The TDA is advantageous in that if spatiotemporal data are available, then the model outputs can be derived for a variety of spatial scales (e.g., site-, habitat-, reach-, and systemic-levels) and many temporal scales (seasonal- to multi-decadal) to match management objectives and a manager's geographical jurisdiction. Selection of state variables for TDA should be ecologically meaningful and allow managers to influence those variables to advance conservation(46). The novel coupling of ecosystem state concepts with multiple TDA tools revealed new information about the ecosystem states and state transitions of the Mississippi River, and this approach (Figure 5) may be transferred to any ecosystem with big data to help classify states and understand their vulnerability to state transitions.

## METHODS

### *Study Area, Ecosystem State Context, and Long-term Data*

The Upper Mississippi River System flows ~2200 km from Minneapolis, MN to the confluence with the Ohio River in Cairo, IL and includes the ~~navigable~~ Illinois River (2). The UMRS has 29 locks and dams have created impounded areas upriver of each lock and dam to maintain a deep main channel for barge traffic, but the locks and dams do not modify water residence time or stop flooding. Lock and dams were installed in the late 1930's which has greatly modified the river's physical and hydrological template. Aquatic plants were abundant after lock and dam construction and then were abruptly reduced in the late 1980's for reasons unknown. Aquatic plants gradually returned and remained abundant since 2010 even under extended flood conditions from 2017–2019 (30). We studied six navigable river reaches named Pool 4, Pool 8, Pool 13, Pool 26, ~~Pool La Grange,~~ and Open River that span multiple environmental gradients like geomorphology, hydrology, and water quality (2).The study reaches were a high restoration priority and had long-term data on water quality.

We assessed ecosystem states based on water quality data at 69,307 sampling sites. Data were collected four times annually (over a two-week sampling episode) in each season from 1993–2020 across six study reaches. This is a 27-year consecutive record, except, except no sampling was done in 2003. The water quality data were collected using a standardized protocol whereby sites were selected by a stratified random sampling design with effort distributed proportionally across strata. Strata were defined based on enduring geomorphic features and included the main channel, contiguous backwaters, impounded areas above the lock and dams, and lotic side channels (47).

Using ecological literature and *a priori* hypotheses (as (as stated in the Introduction), we chose eight water quality variables thought to be important for defining ecosystem states and ensured the variables were not highly correlated (Pearson's $r < 0.45$). The 8 variables included: total nitrogen = mg $L^{-1}$; total

phosphorus = mg $L^{-1}$; dissolved oxygen = mg $L^{-1}$; chlorophyll *a* = μg $L^{-1}$; water depth = cm; temperature = °C; velocity = m $sec^{-1}$; suspended solids = mg $L^{-1}$. Although the TDA can incorporate more variables, we carefully selected 8 variables based on ecological relevance to state theory and the ability to manipulate these variables for restoration (e.g., water velocity and nutrient inputs may be manipulated; see (29).

*Quantifying ecosystem states and the state variables*

To quantify the ecosystem states, we used several topological data analysis tools (Figure 5), which are dimension reduction techniques that interpret complex data (like multi-faceted water quality data, like ours herein) using the data's geometric and topological features. All data have underlaying shape, and this can be visualized and interpreted with TDA to find hidden properties (e.g., ecosystem states and state variables), recognize complex spatial patterns (e.g., how ecosystems may differ across space), and classify large data into meaningful clusters (e.g., ecosystem states based on topological states). We performed five main tasks (Figure 5): 1.) data pre-processing, 2.) applied the undirected, TDA Mapper algorithm, 3.) performed a density-based state assignment and created a directed TDA Mapper graph, 4.) identified state variables, and 5.) created a change detection function to detect state transitions over 27 years.

**Figure 5.** Flow chart that summarized our topological data analysis (TDA) methods in sequential order (arrows and gray text) and the outputted products (boxes with black text and images) for quantifying ecological states and state transitions of the Upper Mississippi River System. The original- and TDA-ready datasets and all analysis codes are available here (DOI: 10.5066/P976O6PP). In the "Undirected TDA Mapper" box, nodes and edges are connected to create a single TDA Mapper structure. n the "Directed TDA Mapper" box, the *example output* describes two local maxima nodes defining two states: a red state containing two nodes and a blue state containing one node. The grey node is a transition node and the sites within the transition nodes are classified into both the red and blue state. In the "change detection" box, we provide a *hypothetical output* of where the dominance of two states (red and blue dashed lines, which are the proportion of sites within those states) temporarily transition from the dominant red state to the blue state around years 13 to 17.

1. Data Pre-processing

The original water quality dataset (accessed 06/01/2021 from https://umesc.usgs.gov/data_library/water_quality/water1_query.shtml), had 204,345 rows (site data) and 133 columns (variables), and contained data from 1993 to 2020, except 2003. We filtered the data by stratified random sampling scheme (and filtered out 'fixed sample sites', which contained ~60% of the data set), surface water collections, and selected 8 continuous variables (because TDA cannot effectively handle binary or ordinal variables). Negative values, which represent readings below the minimum detection limit and was <0.2% of the data, were set to the minimum value of that variable in the entire dataset, and quality flag (QF) codes of '0', 'A', '8', or '64' were set to 'NA.' After we removed three unreasonable and extreme outliers (TN > 25 mg/L), the final data size was 69,307 rows by 8 columns. Three of the variables (total nitrogen, total phosphorus and velocity), per the study design, were only collected at two thirds of the sampling sites. The TDA does not allow missing values, therefore we compared multiple interpolation methods including kriging, inverse distance weighting of various distance thresholds, polynomial regression of various degrees, regression trees, and random forests. We chose a random forests algorithm as the top performing interpolation method (when compared to the mean absolute error to the other methods), which had > 93% accuracy for all interpolated variables. The other occasional missing values were imputed with the median value for each variable. We used robust scaling, so each variable's differing measurement units were accounted for in the multivariate analysis, and robust scaling calculates the distance between input values using the median and interquartile range.

2. The TDA Mapper Algorithm & Topological States Definition

The Topological Data Analysis (TDA) Mapper algorithm visualizes high dimensional data by incorporating dimension reduction techniques and user-selected clustering algorithms to classify sites into topological states (48). We suggest that topological states may indicate ecosystem states if the

input variables are ecologically relevant and state variables are later revealed to classify sites within the TDA structure. The TDA Mapper algorithm takes in point cloud data and produces a 'simplicial complex', which is undirected graph that shows data-shape based on many nodes and edges (Figure 5). The nodes are clusters of sites that are similar based on the data (and the number of sites within each node can vary), and the edges signify interactions or connections among nodes. Nodes that are farther apart in 2-dimensional space indicate the sites are quite different. Essentially, the undirected TDA Mapper creates a single shape of all the data, consisting of nodes and edges. We used Keppler Mapper (a TDA Mapper) written in Python (van Veen et al. 2019) on the TDA-ready data set. The TDA Mapper algorithm was from (48).

The parameters of the TDA Mapper algorithm determine the shape of the data and are chosen by the user. The TDA Mapper parameters include filter function, size of the hypercubes, percent overlap, and clustering algorithm. As of 2022, there were not established methods for selecting these TDA parameter values of our interest with the TDA Mapper, except see (49), so we explored multiple parameters and observed their effects on the simplicial complex. The TDA Mapper graphs using different parameters should be consistent and insensitive to relatively small changes in parameter values. To ensure the shape of the TDA graph persists for various parameter choices, we ran the TDA Mapper for many combinations of these parameter values: cube size ([219,100], [273.75, 125], [328.5, 150], [383.25, 175], [438, 200]) and percent overlap (30%, 40%, and 50%). The TDA Mapper graphs persisted through these parameter combinations and verified our final parameter selections were appropriate and that the TDA Mapper algorithm captured the true shape of the underlying data space.

The final parameters we chose included a principal component analysis as the filter function, the cube size of [273, 125], and DBSCAN as the clustering algorithm with the following justifications. The first two principal components (as determined from principal component analysis, PCA) were used as the filter function. We used the following heuristics to determine the optimal cube size and percent

overlap parameters like (25): each node must contain no more than 10% of the data, and at least 90% of the data must be used in the main TDA Mapper structure that includes the body, tail, and outliers. In other words, at least 90% of the data must be used to create the TDA Mapper and up to 10% of data could be acceptably lost as 'noise.' We quantified and reported in the results the amount of data expressed in the final TDA Mapper output. We used the ratio between the explained variances of the first two PCA components of our filter function as the ratio of the components in the cube size parameter. We used the cube size of [273, 125]] because the PCA with those values explained the most variance. We also used 50% overlap, which was not a sensitive parameter when exploring 40–60% overlaps. For our clustering algorithm, we chose the default parameter 'DBSCAN' within Keppler Mapper. To determine DBSCAN's optimal minimum samples and epsilon parameters, we used the Elbow method that uses a minimum samples parameter of two times the number of data attributes. The epsilon values are selected by the following process:

1. Finding the average distance of each data points k-nearest neighbors (kNN)
2. Sorting and plotting the averages kNN distances for all data points
3. Finding the point of greatest curvature. Since eight water quality variables were used to produce the simplicial complex, we used 16 as our minimum samples. The epsilon value used was 1.5 and calculated using the Kneed package (Satopa et al. 2022).

3. Density-based state assignment

Next, we use a topological states algorithm to refine the simplicial complex (i.e., the undirected TDA Mapper output) into a directed TDA Mapper graph, which further refines the data's shape into more topological states (Figure 5). We applied a density algorithm to the undirected TDA Mapper graph to produce a directed graph with defined states as adopted and modified from (25). We used k-nearest neighbor (kNN) distance. Each node is assigned a density of

$$D(V) = \frac{n^2}{\sum_{i \in V} kNN(i,k)}$$

, where $n^2$ counts for differing node sizes and $kNN(i,k)$ is the kNN distance of point $i$ defined as

$$kNN(i,k) = \frac{\sum_{j \in N(k)_i} d_{ij}}{k}$$

where $N(k)_i$ is the set of $k$ nearest points to the point $i, k$ is 10% of the number of samples in the dataset, and $d_{ij}$ is the distance between points $i$ and $j$. This density assignment allows converting the undirected TDA Mapper graph to a directed graph by replacing the edge between two nodes by an arrow pointing from the node with smaller density to the node with larger density. The "Topological state definitions" in algorithm 2 and Figure 5 shows how a Mapper graph is converted to a directed graph to quantify local maxima nodes.

The directed graph is grouped into topological states (and conceptually considered ecosystem states herein), and these states are determined by *local maxima* nodes towards which every arrow connected to them points (Figure 5). In other words, the number of local maxima nodes is equal to the number of states detected. The distance between a node and a local maxima node is defined to be the smallest possible number of edges connecting them. A single water quality sampling site may sometimes be placed into more than one node, and thus classified in more than one state, if two nodes in different states have an edge between them and intersection of points. If a node is equidistant from two or more local maxima, that node is called a 'transition node' and is assigned to both local maxima (i.e., classified in both states). Sampling sites with more than one state classification or in transition nodes represented sites that are not easily classified by the topological state's definition.

4. Identifying state variables

A state variable is statistically considered aa variable most positively associated with data clustering into a given topological state and the variable's data distribution is different from that of other states. We included eight water quality variables for the TDA to capture the multidimensionality of ecosystem state

concepts. To determine state variables, we examined the gradients of each water quality variable's data ranges displayed on the TDA Mapper structure in relation to where the state was defined on the TDA Mapper graph that classified states. We also compared the data distributions among the five state classifications using boxplots for each variable. If the median (50$^{th}$ percentile) and boxplot whiskers (25$^{th}$–75$^{th}$ percentiles) did not overlap among the states, we considered that a likely state variable. We did not run formal statistical analysis for 'statistical significance' among the five boxplots (e.g., analysis of variance among the states) because the large number of data points (*n*=6969,307) and degrees of freedom precluded that option due to risk of Type 1 error (i.e., false positive).

### *Quantifying ecosystem state transitions*

5. Change detection function

We tested whether states were stable or transitioned over 27 years. We analyzed the state composition of each reach during each season (autumn, winter, spring, and summer). On average, each season-reach combination contained 122 datapoints. We defined 'proportion values' for a reach-season-state pair as:

$$f(x_{state}) = \frac{x_{state}}{n}$$

where $x_{state}$ is the number of sites within a reach classified within a particular state during that season, and $n$ is the total number of sites in any state collected during that reach-season combination. A hypothetical example showing aa single state transition between two states based on the temporal changes in proportion values is in Figure 5.

## ACKNOWLEDGMENTS

The data collection, data analysis, manuscript preparations, and authors DL and WB were funded as part of the U.S. Army Corps of Engineers' Upper Mississippi River Restoration Program (https://umesc.usgs.gov/ltrm-home.html). The National Science Foundation's "Research Experience for Undergraduates" (REU) Program Award Number:1559663 funded authors CM, AS, AL, KD, FM for data analysis and manuscript preparation. This research was performed in part using computational resources supported by the Academic & Research Computing group at Worcester Polytechnic Institute. We sincerely thank Gregory Sandland, James Pierce, Roger Haro, and Richard Erickson for REU Program leadership and support in 2021. We thank Douglas Krouth (UWL) for initiating the first TDA Mappers. We thank four anonymous reviewers for greatly improving this manuscript. Any use of trade, firm, or product names is for descriptive purposes only and does not imply endorsement by the U.S. Government.

## REFERENCES

1. Dodds WK, Whiles MR. Freshwater Ecology: Concepts and Environmental Applications of Limnology. 3rd ed. Academic Press; 2019.
2. Houser J, Bouska K, De Jager N, Larson D, Ickes B, Jankowski K, et al. Ecological Status and Trends of the Upper Mississippi and Illinois Rivers. U.S. Geological Sur-vey Open-File Report 2022–1039; 2022. 1–218 p.
3. Bestelmeyer B, Ash A, Brown JR, Densambuu B, Fernández-Giménez M, Johanson J, et al. State and transition models: theory, applications, and challenges. In: Briske D, editor. Rangeland Systems: Processes, Management and Challenges [Internet]. Cham: Springer; 2017. p. 303–45. Available from: http://www.springer.com/series/412
4. Gunderson L, Holling C. Panarchy: understanding transformations in systems of humans and nature. Washington D.C.: Island Press; 2002.
5. Carpenter S, Walker B, Anderies JM, Abel N. From Metaphor to Measurement: Resilience of What to What? In: Ecosystems. 2001. p. 765–81.
6. Suding KN, Gross KL, Houseman GR. Alternative states and positive feedbacks in restoration ecology. Trends Ecol Evol. 2004;19(1):46–53.

7. Biggs R, Peterson GD, Rocha JC. The regime shifts database: A framework for analyzing regime shifts in social-ecological systems. Ecol Soc. 2018 Oct 1;23(3):018473.

8. Scheffer M. Ecology of shallow lakes. 1st ed. London: Kluwer Academic Publishers; 1993.

9. Larson DM, Cordts SD, Hansel-Welch N. Shallow lake management enhanced habitat and attracted waterbirds during fall migration. Hydrobiologia. 2020;846(16):3364–79.

10. Steneck RS, Graham MH, Bourque BJ, Corbett D, Erlandson JM, Estes JA, et al. Kelp forest ecosystems: biodiversity, stability, resilience and future. Environ Conserv [Internet]. 2002;29(4):436–59. Available from: https://about.jstor.org/terms

11. Bestelmeyer BT, Okin GS, Duniway MC, Archer SR, Sayre NF, Williamson JC, et al. Desertification, land use, and the transformation of global drylands. Front Ecol Environ. 2015 Feb 1;13(1):28–36.

12. Briggs JM, Blair JM, Mccarron J. An ecosystem in transition:cause and consequences of the conversion of mesic grassland to shrubland. Bioscience. 2005;55(7):561–72.

13. Mumby PJ, Hastings A, Edwards HJ. Thresholds and the resilience of Caribbean coral reefs. Nature. 2007 Nov 1;450(7166):98–101.

14. Larson DM, Dodds WK, Veach AM. Removal of Woody Riparian Vegetation Substantially Altered a Stream Ecosystem in an Otherwise Undisturbed Grassland Watershed. Ecosystems. 2019;22(1):64–76.

15. Zinnert JC, Nippert JB, Rudgers JA, Pennings SC, González G, Alber M, et al. State changes: insights from the U.S. Long Term Ecological Research Network. Ecosphere. 2021 May 1;12(5).

16. Martin BE, Walsh JR, Vander Zanden MJ. Rise of a native apex predator and an invasive zooplankton cause successive ecological regime shifts in a North Temperate Lake. Limnol Oceanogr. 2022 Feb 1;67(S1):S163–72.

17. Dent CL, Cumming GS, Carpenter SR. Multiple states in river and lake ecosystems. Philos Trans R Soc B Biol Sci. 2002 May 29;357(1421):635–45.

18. Bouska KL, Houser JN, De Jager NR, Drake DC, Collins SF, Gibson-Reinemer DK, et al. Conceptualizing alternate regimes in a large floodplain-river ecosystem: Water clarity, invasive fish, and floodplain vegetation. J Environ Manage [Internet]. 2020 Jun;264:110516. Available from: https://linkinghub.elsevier.com/retrieve/pii/S0301479720304503

19. Burdis RM, DeLain SA, Lund EM, Moore MJC, Popp WA. Decadal trends and ecological shifts in backwater lakes of a large floodplain river: Upper Mississippi River. Aquat Sci. 2020 Apr 1;82(2):1–19.

20. Shang J, Zhang W, Chen X, Li Y, Niu L, Wang L, et al. How environmental stress leads to alternative microbiota states in a river ecosystem: A new insight into river restoration. Water Res. 2021 Sep 15;203:1–9.

21. Larson DM, Carhart A, Lund E. Aquatic vegetation types identified during early and late phases of vegetation recovery in the Upper Mississippi River, in press. Ecosphere. 2023;

22. Diamond JS, Moatar F, Cohen MJ, Poirel A, Martinet C, Maire A, et al. Metabolic regime shifts and ecosystem state changes are decoupled in a large river. Limnol Oceanogr. 2022 Feb 1;67(S1):S54–70.

23. Ibanez C, Penuelas J. Changing nutrients changing rivers. Science (80- ). 2019 Aug 16;365(6454):637–8.

24. Dale MRT. Applying Graph Theory in Ecological Research. 1st ed. Cambridge: Cambridge University Press; 2017.

25. Chang WK, VanInsberghe D, Kelly L. Topological analysis reveals state transitions in human gut and marine bacterial communities. npj Biofilms Microbiomes. 2020 Dec 1;6(1):1–10.

26. Bendick R, Hoylman ZH. Topological data analysis reveals parameters with prognostic skill for extreme wildfire size. Environ Res Lett. 2020 Oct 1;15(10).

27. Yuvaraj M, Dey AK, Lyubchich V, Gel YR, Poor VH. Topological clustering of multilayer networks. Proc Natl Acad Sci. 2021;118(21):1–9.

28. Blevins AS. Extending topological data analysis in biological systems. University of Pennsylvania; 2019.

29. McCain K, Schmueker S, De Jager N. Habitat Needs Assessment - II : Linking Science to Management Perspectives. Rock Island, IL: U.S. Army Corps of Engineers; 2018.

30. Larson DM, Lund E, Carhart A, Drake D, Houser J, Bales K, et al. Status and Trends of Upper Mississippi River's Aquatic Vegetation from 1998 to 2019. In: Houser J, Bouska K, De Jager N, Ickes B, Jankowski KJ, Larson D, et al., editors. Status and Trends of the Upper Mississippi and Illinois Rivers. 3rd ed. U.S. Geological Sur-vey Open-File Report 2022–1039; 2022. p. 1–218.

31. Greenblum S, Turnbaugh PJ, Borenstein E. Metagenomic systems biology of the human gut microbiome reveals topological shifts associated with obesity and inflammatory bowel disease. Proc Natl Acad Sci U S A. 2012 Jan 10;109(2):594–9.

32. Mason TJ, Keith DA, Letten AD. Detecting state changes for ecosystem conservation with long-term monitoring of species composition: Ecol Appl. 2017 Mar 1;27(2):458–68.

33. Islambekov U, Yuvaraj M, Gel YR. Harnessing the power of topological data analysis to detect change points. In: Environmetrics. John Wiley and Sons Ltd; 2020.

34. Hilt S, Köhler J, Kozerski HP, van Nes EH, Scheffer M. Abrupt regime shifts in space and time along rivers and connected lake systems. Oikos. 2011 May;120(5):766–75.

35. Scheffer M, Bascompte J, Brock W, Brovkin V. Early-warning signals for critical transitions. Nature [Internet]. 2009 [cited 2017 Aug 21];461(7260):53–9. Available from: http://search.proquest.com/openview/7734e5b77c0e9dd2198216184823e65d/1?pq-origsite=gscholar&cbl=40569

36. Suding KN, Hobbs RJ. Threshold models in restoration and conservation: a developing framework. Vol. 24, Trends in Ecology and Evolution. 2009. p. 271–9.

37. Scheffer M, Carpenter SR. Catastrophic regime shifts in ecosystems: Linking theory to observation. Trends Ecol Evol. 2003;18(12):648–56.

38. Biggs R, Carpenter SR, Brock WA. Turning back from the brink: Detecting an impending regime shift in time to avert it. Proc Natl Acad Sci [Internet]. 2009;106(3):826–31. Available from: www.pnas.org/cgi/content/full/

39. Jiang J, Huang ZG, Seager TP, Lin W, Grebogi C, Hastings A, et al. Predicting tipping points in mutualistic networks through dimension reduction. Proc Natl Acad Sci U S A. 2018 Jan 23;115(4):E639–47.

40. Samhouri JF, Levin PS, Ainsworth CH. Identifying thresholds for ecosystem-based management. PLoS One. 2010 Jan 26;5(1):1–10.

41. U.S. Army Corps of Engineers. Upper Mississippi River Restoration Program [Internet]. 2022 [cited 2022 Aug 29]. Available from: https://www.mvr.usace.army.mil/Missions/Environmental-Stewardship/Upper-Mississippi-River-Restoration/

42. De Jager NR, Rogala JT, Rohweder JJ, Van Appledorn M, Bouska KL, Houser JN, et al. Indicators of Ecosystem Structure and Function for the Upper Mississippi River System. OFR20181143. 2018.

43. U.S. Environmental Protection Agency. Ambient Water Quality Criteria Recommendations: Information Supporting the Development of State and Tribal Nutrient Criteria Lakes and Reservoirs in Nutrient Ecoregion IX. 2000;EPA 822-B-00-019(December):1–108.

44. Carpenter S, Brock W. Rising variance: a leading indicator of ecological transition. Ecol Lett. 2006;9:311–8.

45. Capon SJ, Lynch AJJ, Bond N, Chessman BC, Davis J, Davidson N, et al. Regime shifts, thresholds and multiple stable states in freshwater ecosystems; a critical appraisal of the evidence. Sci Total Environ. 2015 Nov 15;534:122–30.

46. Groffman PM, Baron JS, Blett T, Gold AJ, Goodman I, Gunderson LH, et al. Ecological thresholds: The key to successful environmental management or an important concept with no practical application? Ecosystems. 2006;9(1):1–13.

47. Soballe DM, Fischer JR. Long Term Resource Monitoring Program Procedures: Water Quality Monitoring. U.S. Army Corps of Engineers Technical Report 2004-T002-01; 2004. 1–134 p.

48. Singh G, Mémoli F, Carlsson G. Topological Methods for the Analysis of High Dimensional Data Sets and 3D Object Recognition. Eurographics Symposium on Point-Based Graphics. Eurographics Symposium on Point-Based Graphics; 2007.

49. Kamruzzaman M, Kalyanaraman A, Krishnamoorthy B, Hey S, Schnable PS. Hyppo-X: A Scalable Exploratory Framework for Analyzing Complex Phenomics Data. IEEE/ACM Trans Comput Biol Bioinforma. 2021 Jul 1;18(4):1535–48.

**FIGURES**

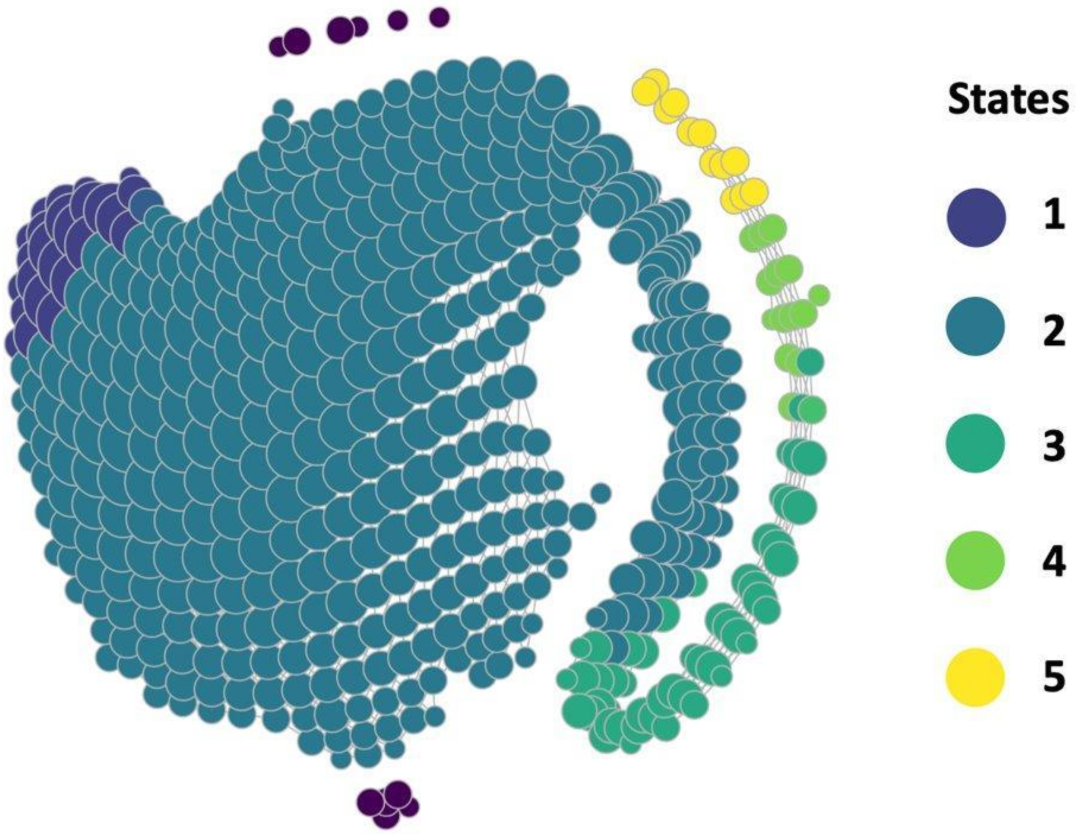


**Figure 1.**

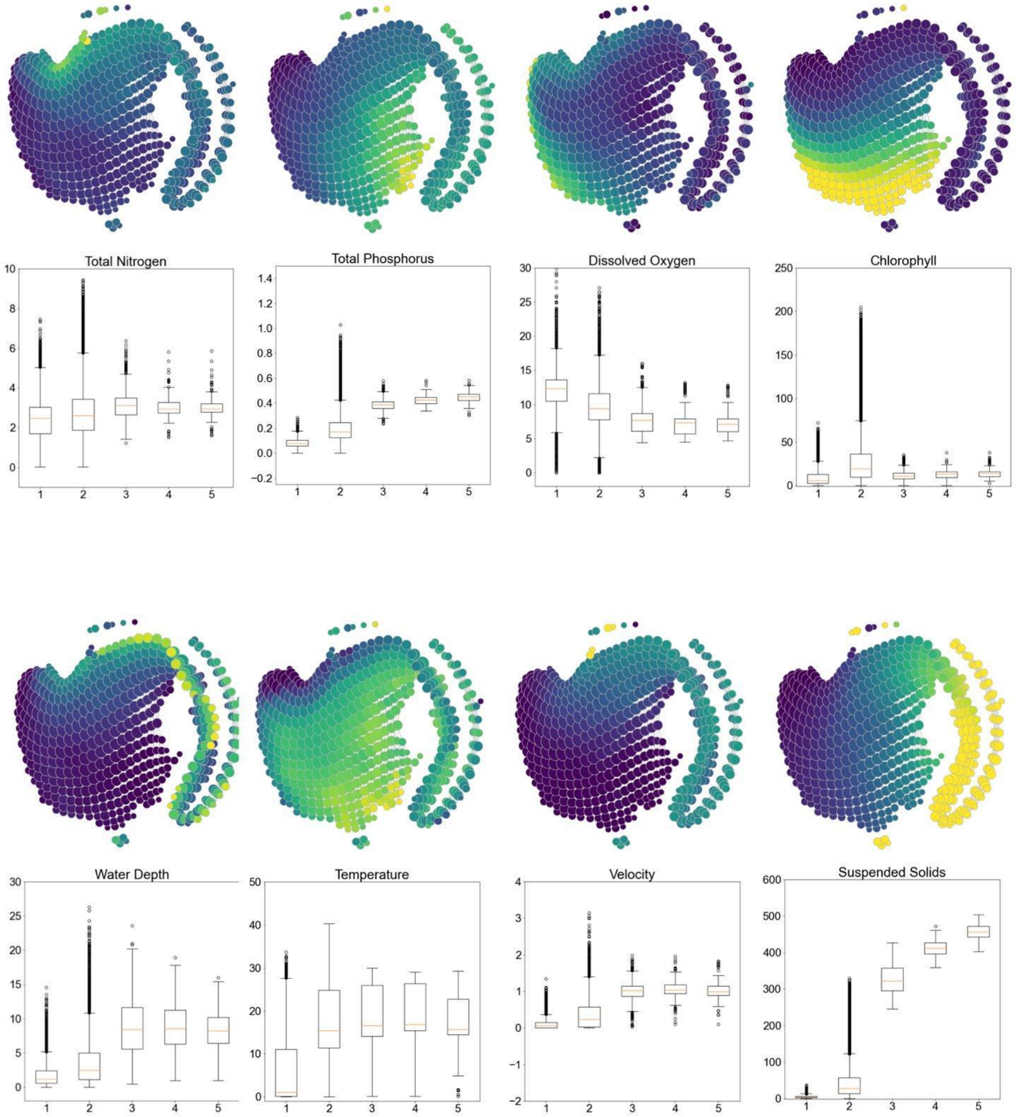
Total Nitrogen
Total Phosphorus
Dissolved Oxygen
Chlorophyll
Water Depth
Temperature
Velocity
Suspended Solids

**Figure 2.**

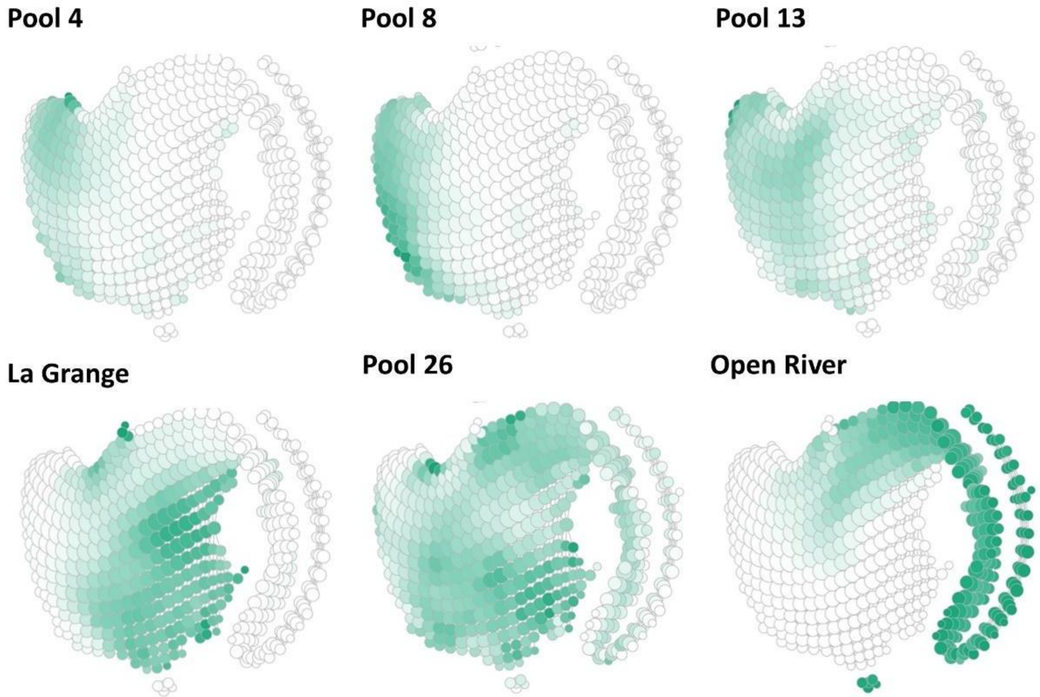


**Figure 3.**

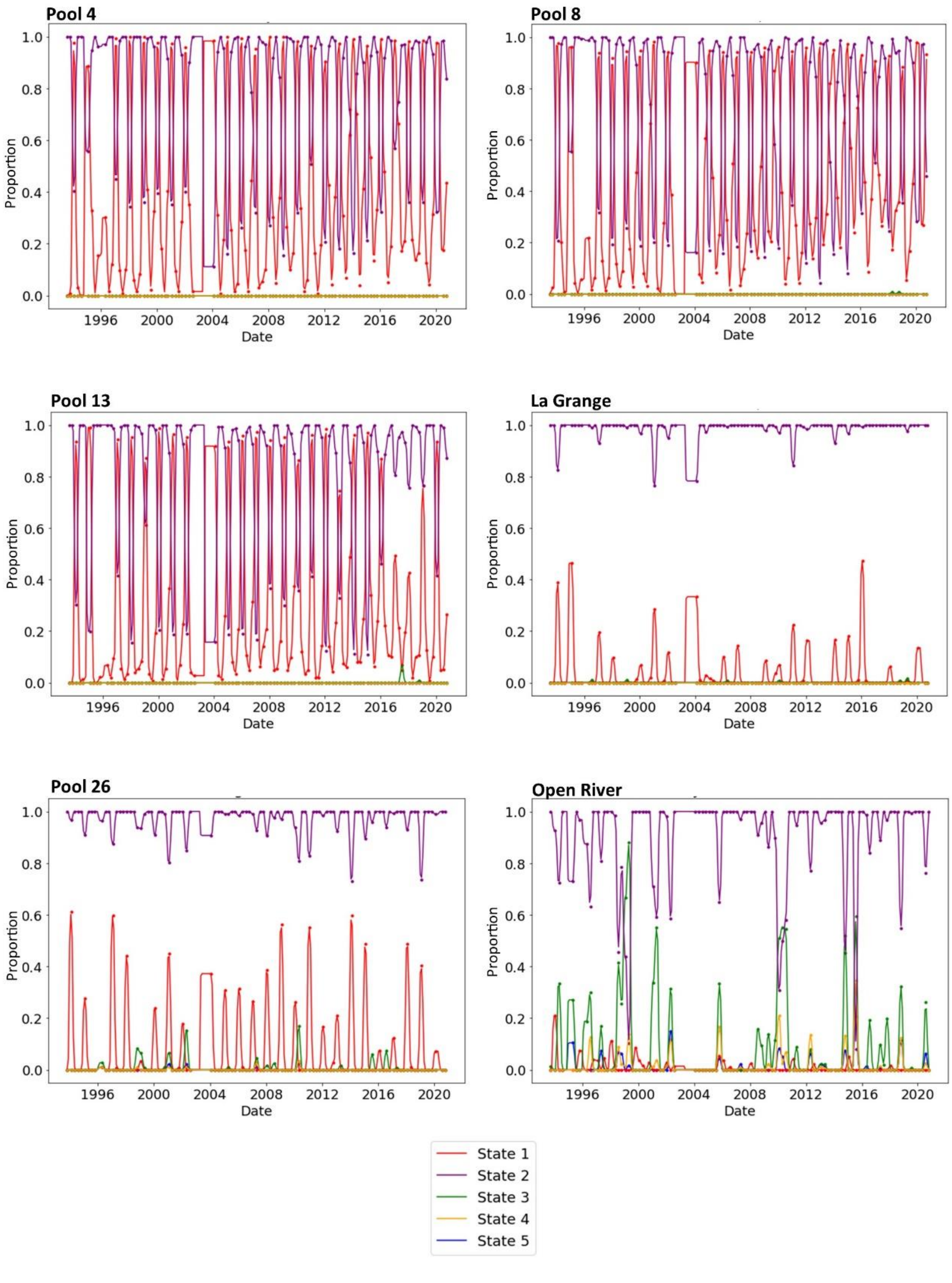
Pool 4
Pool 8
Pool 13
La Grange
Pool 26
Open River
Proportion
Date
1996 2000 2004 2008 2012 2016 2020
0.0 0.2 0.4 0.6 0.8 1.0
State 1
State 2
State 3
State 4
State 5

**Figure 4.**

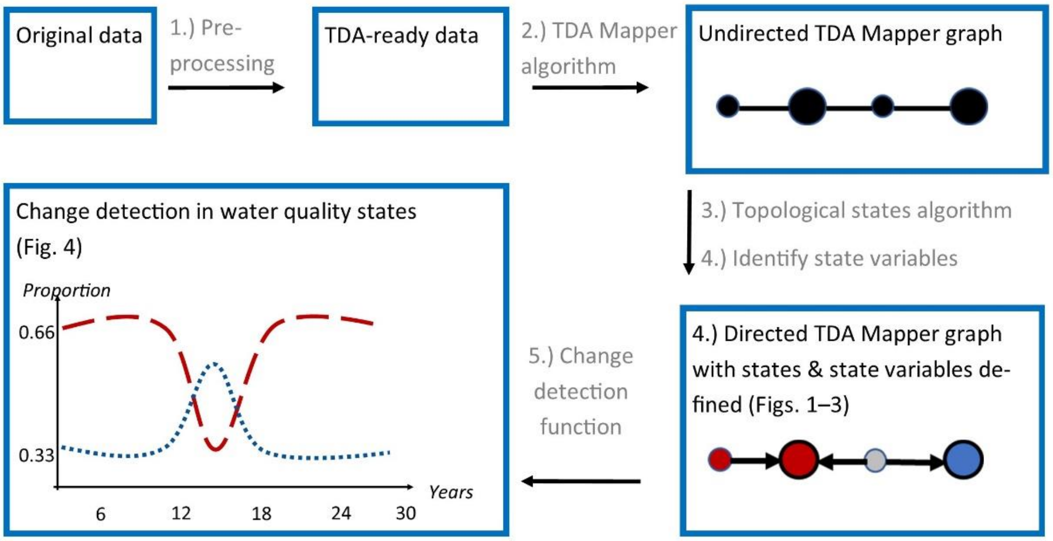
Original data
1.) Pre-processing
TDA-ready data
2.) TDA Mapper algorithm
Undirected TDA Mapper graph
3.) Topological states algorithm
4.) Identify state variables
4.) Directed TDA Mapper graph with states & state variables defined (Figs. 1–3)
5.) Change detection function
Change detection in water quality states (Fig. 4)
Proportion
0.66
0.33
Years
6
12
18
24
30

**Figure 5.**

## SUPPORTING INFORMATION

**S1 Figure**. Graphical output from topological data analysis (TDA Mapper) showing a strong seasonal component to the water quality data for the Upper Mississippi River System, USA. Data inputs included 8 water quality variables collected four times each year from 1993–2020 at a total of 69,307 sampling sites. The graph is comprised of nodes (circles), which cluster sampling sites along the river based on water quality similarities, and the edges (lines) show statistical connections among nodes. We highlighted in color the seasons in which sites were sampled. Seasonality can be connected to ecosystem states by referencing Figure 1 positions; for example, winter is typically characterized by State 1.

Click here to access/download
**Supporting Information**
Larson TDA river PLOS 15March2023 supporting information.docx

# Title: Quantifying ecosystem states and state transitions of the Upper Mississippi River System using topological data analysis

**Commented [HAN1]:** Great work!

**Commented [LDM2R1]:** Thank you to Dr. Anna Hess for the USGS Supervisory Review to ensure peer reviews were reconciled and supported by the Agency.

**Authors:** Danelle M. Larson[1‡],Wako Bungula[2], Casey McKean[3], Alaina Stockdill[3], Amber Lee[3], Frederick Miller[3], Killian Davis[3]

**Affiliations:**
[1] U.S. Geological Survey, 2630 Fanta Reed Road, La Crosse, WI 54603; dmlarson@usgs.gov; no fax; ORCID: 0000-0001-6349-6267; [‡] Corresponding Author: dmlarson@usgs.gov
[2] University of Wisconsin La-Crosse, Department of Mathematics and Statistics, 1027 Cowley Hall, La Crosse, WI 54603
[3] University of Wisconsin La-Crosse, Research Experience for Undergraduates Program, 1027 Cowley Hall, La Crosse, WI 54603

**Corresponding author:** Danelle M. Larson, dmlarson@usgs.gov

**Commented [LDM3]:** We appreciate the edits by USGS internal reviewer, Kathi Jo Jankowski, and will accept all tracked changes. Each comment was responded to herein.

**Commented [LDM4]:** We sincerely thank three anonymous reviewers through the journal, PLOS Computational Biology. All revisions are tracked here and comments responded to in the separate file named, "PLOS reviews_response to reviewers.docx"

**Commented [JKJ5]:** Do they have any other institutional affiliations (their colleges) to include? Might be good for their colleges to be listed

**Commented [LDM6R5]:** We preferred to keep it to UWL because that was the funding and affiliated college with this work.

**Running title:** quantifying riverine states via topology

## ABSTRACT

Aquatic systems worldwide can exist in multiple ecosystem states (i.e., a recurring collection of biological and chemical attributes), and effectively characterizing multidimensional states will aid protection of desirable states and guide restoration~~restorations~~. The Upper Mississippi River System is a large, floodplain river spanning 2200 km with a 30-year water quality monitoring data set and ~~to with~~and ~~information needs to depict~~ ecosystem states need depicting ~~and~~ that can guide river restoration. We coupled this highly dimensional dataset with multiple topological data analysis (TDA) techniques to classify ecosystem states, identify state variables, and detect state transitions over 30 years. Across the entire ~~river~~ system, TDA identified five ecosystem states. State 1 was characterized by exceptionally clear, clean, and ~~cold water~~cold-water conditions typical of winter (i.e., a 'clear-water state'); State 2 had the greatest range of environmental conditions (i.e., a 'status-quo state'); and States 3, 4, and 5 had extremely ~~very~~ high concentrations of suspended solids (i.e., 'turbid-states', with State 5 as the most turbid). The TDA mapped clear patterns of the ecosystem states across several riverine navigation reaches~~pools,~~ and seasons~~, and habitat types~~ that furthered ecological understanding. State variables were identified as suspended solids, chlorophyll *a*, and total phosphorus, which~~similar to~~ are also state~~s~~ variables of shallow lakes worldwide. The TDA change detection function showed short-term state transitions based on seasonality and episodic events, and~~as well as provided~~ also provided evidence of gradual, long-term changes due to water quality improvements over three decades. ~~Some pools underwent seasonal state transitions each year, entering State 1 in winter and State 2 in other seasons. One pool transitioned sporadically several times between States 2 and 3. The TDA was a novel technique that showed the river was characterized by predictable ecosystem states and a few state variables, and pools experienced state transitions based on seasonal or episodic events.~~ These results can inform decision making and guide actions for regulatory and restoration agencies by assessing the status and trends of this important river and ~~providing~~ provide quantitative targets for state variables. The TDA change~~ if management aims to purposely shift the turbid-state to more desirable conditions~~

~~like States 1 and 2. The TDA correlation function~~ detection function may serve as a new tool for predicting the vulnerability to undesirable state transitions in this system and other ecosystems with sufficient data. ~~These~~ Coupling ecosystem state concepts and TDA ~~methodologies~~ tools can be transferred to any ecosystem with large data to help classify states and understand their vulnerability to state transitions.~~transferred to any ecosystem to help classify states and possibly predict vulnerability and causes of state transitions.~~

**Keywords:** alternative stable ecological state, ~~floodplain river,~~ ecological regime shift, ecosystem state change, topology, topological state, water quality, vulnerability

## AUTHOR SUMMARY

**Commented [LDM7]:** Added per the requirements of the journal, PLOS Computational Biology. It's a 200 word statement intended to be understood by laymen. The abstract can be more technical

The Mississippi River in the USA is a large, complex ecosystem that had many changes to water quality since the 1990s. We used a new set of mathematical tools called 'topological data analysis' to help identify common water quality conditions or 'states' of this important river. We also explored when and why the states have changed over the past 25 years.~~and vulnerability to change.~~ The tools identified five common water quality states, distinguished by nutrients~~either by~~ and clear waters or muddy waters. State changes were commonly detected as seasonal transitions in the higher latitude reaches. The state changes also~~were not abrupt but~~ showed that ~~rather~~ water quality had improved or declined gradually over many years, depending on the river reach. Reaches~~River reaches~~ named Pool 4 and Pool 8 had become clearer over time. Pool 13 became muddier over time with several undesirable state changes. Three other river reaches were consistently muddy. Our study is a good example of how these topological data analysis tools may help others managing ecosystems by improving communications about what states exist and which are desirable. ~~(for example, the clear water state is desirable).~~ Using these tools with long-term data can also alert ecosystem managers to the ongoing, complex changes and vulnerability to undesirable changes so they ~~can~~ may intervene with restoration.

**Author contributions:**

~~DL and WB conceptualized study, secured~~Larson and Bungula contributed to conceptualization, funding acquisition, formal analysis, data curation, supervision, and writing-original draft preparation. ~~funding, curated data, and shared data; a~~All authors ~~prepared data and analyzed data; AS, AL, and CM wrote final analysis codes and created figures; DL and WB wrote first paper draft; all authors~~contributed to data curation, investigation, visualization, and writing-review and editing. ~~edited paper drafts and approved of journal submission.~~

**Data availability statement:**

All data were publicly accessed on 1 June, 2021 through the Upper Mississippi River Restoration Program's *Long-term Resource Monitoring* (https://umesc.usgs.gov/ltrm-home.html). ~~The data used in this analysis is provided in the attached Supporting Information file, 'Mississippi River TDA data set.csv.'~~ Our final datasets and analysis scripts ~~will be~~are publicly and permanently archived in ~~GitLab~~ ScienceBase (DOI: 10.5066/P976O6PP~~XXXXXXXX~~) ~~following peer review (in progress) through the U.S. Geological Survey (in progress) and immediately prior to publication~~.

# INTRODUCTION

Many ecosystems worldwide are undergoing severe and rapid changes and managing ecosystem states and states transitions are a time-sensitive and difficult challenge. Large floodplain rivers are especially degraded and continue to experience major changes to hydrology and water quality (1,2). Monitoring ecosystem states and states transitions with long term data and advanced analytical tools is critical for protecting and restoring ecosystems that humanity values or depends on (3).

Ecosystem states, or alternative states, ~~(i.e.,~~are a recurring collection of biological and chemical attributes that arise from structuring processes and have some resilience to changes within its state space~~; for detailed conceptual and applicable theory, please review~~ (4–6)~~)~~. Characterizing and defining the state is always multidimensional, but typically only a few ~~'~~state variables~~'~~ can properly characterize

**Commented [LDM8]:** Reviewer 3 suggested changing 'six pools' to 'six reaches' to better reflect the geomorphology. We did this throughout.

**Commented [JKJ9]:** Would be helpful to add a citation for this definition – doesn't it also have to do with their resilience/resistance to change?

In general, I think you may want to better describe state theory upfront to link it to the way you statistically evaluate states using TDA – so the linkages between the theory and the analysis are clear for the reader

**Commented [LDM10R9]:** We added a new paragraph that describes and define ecosystem states, state variables, why it matters, and cites seminal papers. Thank you!

**Commented [HAN11]:** I would suggest retaining the references only here, and deleting this small section so that it reads more smoothly

**Commented [LDM12R11]:** Deleted the small section as suggested

the ecosystem state. State variables are features of the biological community and physical environment that characterize the structure and functions of an ecosystem (6). State transitions are either temporary or permanent changes to state variables that alter the ecosystem state. In contrast, 'regime shifts' are state transitions that are substantial~~ and~~ , persistent through time, and difficult to reverse with restoration (7). Identifying ecosystem states and ~~its~~ the associated state variables is essential for describing the ecosystem and setting clear and reasonable ecological restoration targets.

Ecosystem states are well defined in many types of ecological systems, but not for large rivers. Many ecosystems have at least two alternative states often defined by the lower trophic levels, such as the plant-dominated or phytoplankton-dominated states in lakes (8,9), marine kelp forest or kelp-deforestation (10), mesic grasslands or desertification (11), mesic grasslands or shrublands (12), and diverse coral reefs or macroalgal-dominated reefs (13). ~~State characterization is always multidimensional, and typically has only a few state variables although sometimes is defined by complex biological communities (Benedek et al. 2007, Larson et al. 2022).~~ A few examples of state transitions include the restoration of aquatic vegetation and water quality in shallow lakes (9), altered disturbance regimes and subsequent restorations in tallgrass prairies (14,15), and successive state shifts and a novel state arising from multiple stressors in a deep lake (16).

Ecosystem states have been conceptualized (17,18) but not well tested in rivers until recently (19–22). The lack of research may be attributed to the difficulty in defining a riverine~~state in a river because rivers are highly dynamic, routinely disturbed, and~~ states because ~~-~~the multivariate methodology to tackle a state definition is complex and data is often sparse. The global scientific and management community calls for applying an ecosystem states framework to rivers, while acknowledging most rivers do not have sufficient data to properly evaluate states (7,18,23). When sufficient data are available, identifying the states and drivers of the transition between states~~, and their constraints~~ is key for ecological restoration planning, post-project monitoring, and adaptive management (6).

**Commented [HAN13]:** A suggestion to strengthen this statement (which is already very good)

**Commented [LDM14R13]:** Accepted insertion

**Commented [JKJ15]:** Is that always true that they are defined by primary producers?

**Commented [LDM16R15]:** No always true. Could be defined by the presence or absence of a top predator, for example.

Changed to 'often defined by lower trophic levels'…

Thanks!

**Commented [JKJ17]:** Do you mean presence or absence of kelp forests here? A little confusing as stated since I think of terrestrial forests when you say "deforestation"…

**Commented [LDM18R17]:** Clarified, 'kelp-deforestation', because its human caused loss (not just kelp forest absence)

**Commented [JKJ19]:** Not sure what you mean by "constraints"? The variables that maintain a system in a given state?

**Commented [LDM20R19]:** Removed 'constraints' because another definition that does not need defining for this Introduction. Thanks.

State concepts are complex and multidimensional, and therefore requires substantial data, advanced and novel analysis techniques for disentangling patterns, and expertise for its ecological interpretations. More analytical tools for 'big data' that can reasonably predict ecosystem states and impending state transitions will help conserve, restore, and manage ecosystem resilience (3). Topological data analysis (TDA) provides a powerful suite of tools for disentangling patterns and relationships among multidimensional data and presenting data visually (24). Tools of TDA~~ have~~ effectively shown changes in the human gut following bacterial disturbance (25), predicted wildfire severity (26), and assessed vulnerability of residential homes to climatic changes (27). The use of TDA remains rare in ecology, although TDA may be an ideal tool for many ecological applications because of its multidimensional framework (28).

**Objectives and Hypotheses:**

We provided an analysis of ecosystem states and transitions using topological data analysis (TDA) tools to better characterize the Upper Mississippi River System, which is a significant ecosystem~~ for~~ to humans and wildlife (2). We hypothesized that this river would be in at least two states (18,23): a 'status~~clear-water~~status-quo state' that is common and characterized by a~~clean and clear water~~a broad range of turbidity and nutrient concentrations, and a 'turbid-state' characterized by extremely high turbidity due to high suspended solids and chlorophyll *a* (Hypothesis 1). We expected at least three state variables similar~~ based on nutrients and turbidity~~ to regional shallow lake ecosystems (9) that include total phosphorus, suspended solids, and chlorophyll *a* (Hypothesis 2). We ~~) but included up to eight hypothesized state variables within TDA. We explored whether~~hypothesized that different riverine~~ pools~~ were in different states (Hypothesis 3) given their differing water quality conditions and restoration priorities (2,29). We ~~expected~~ hypothesized TDA would detect an abrupt state transition around years 2007~~2008~~2007–2010 when aquatic vegetation rebounded and stabilized at high prevalence in several

**Commented [LDM21]:** I needed to change this terminology throughout the manuscript. The Mississippi is not in a clear-water state, 95% of the time!, as the data suggests. I renamed the body 'status-quo state'. State 1 could be a clear-water state given its conditions (Figure 2).

**Commented [JKJ22]:** You don't define a "state variable", maybe useful to define ecological states and state variables earlier?

**Commented [LDM23R22]:** Great idea. We created a new paragraph starting on line 81 (second Intro paragraph) to do so.

**Commented [JKJ24]:** This differed among pools though? Earlier in Pool 8 than other places?

**Commented [LDM25R24]:** True. We rephrased our hypothesis with more detail: "). We expected TDA would detect an abrupt state transition around years 2007–2010 when aquatic vegetation rebounded and stabilized at high prevalence in several upper pools of the river (Burdis et al. 2020, Larson et al. 2022) and water quality conditions improved (Houser et al. 2022) (Hypothesis 4a)."

upper reaches~~pools of the river~~ and water quality conditions improved~~–~~ (19,30) (Hypothesis 4a).~~), and~~ We further expected that TDA could also detect gradual water quality improvements based on the proportion of sites leaving the 'turbid-state' and entering a '~~clear-water~~ status-quo state' (Hypothesis 4b).

## RESULTS

*How many ecosystem states were detected in the UMRS? Hypothesis 1*

The TDA Mapper inputted 8 water quality variables collected from 69,307 sites over 27 years onto a single TDA structure~~graph~~ (Figure 1). The TDA Mapper graph output ~~has~~ had six connected components that~~where the main component represented~~ included more than 99% of the data. ~~(Figure 1).~~ Only <1% of the data points ~~and~~ were excluded from the main TDA structure as 'outliers' or 'noise' (Figure 1 dark violet nodes). The main ~~component~~ TDA structure had a shape of a "body" (containing ~90% of the data) and a "tail" ~~","~~ (containing about 10% of the data), suggesting there were ~~at least~~ two main ecosystem states ~~as determined by topology~~ (in support of Hypothesis 1).

**Figure 1.** Graphical output from topological data analysis (TDA Mapper) coupled with a density-based state assignment of the ~~potential~~ topological and ecosystem states for the Upper Mississippi River System, USA. Data inputs included 8 water quality ~~state~~ variables collected four times each year from 1993–2020 at a total of 69,307 sampling sites. The graph is comprised of nodes (circles), which cluster sampling sites along the river based on water quality similarities, and the edges (lines) show statistical connections among nodes. An undirected ~~TDA Mapper~~ graph (visualized by ignoring color scheme and focusing only on data shape) showed two topological states, indicated by the tail being less connected to the main body and thus having~~different topological states~~ differing topology. The TDA Mapper was then transformed into a directed TDA Mapper graph using the topological states algorithm that further refined the shape into five ~~topological~~ states (color coded). ~~, which~~ The topological states may ~~can~~ be conceptualized as ecosystem states, especially when further exploring data within the nodes (circles) to understand possible ecosystem state variables that classified each site within a node and distinguish among topological states. ~~,), and most,~~ ~~Most sites were within State 2 of the main body.~~ The dark purple nodes not connected to the main structure (<1% of sites) were outliers and not classified within a state.

**Commented [JKJ26]:** I think there was a more gradual change in water quality conditions over time..

**Commented [LDM27R26]:** Yes, we agree (hypothesis 4b). If there was a more abrupt state transition, we expect it would occur around years 2008-2010 due to plants.

**Commented [HAN28]:** I'm assuming that including the caption here in the text is in the PLOS Comp Biology guidelines but I could not find it, so I just suggest double-checking to make sure?

**Commented [LDM29R28]:** Yes, its guidelines of PLOS. Thank you.

**Commented [LDM30]:** We don't know which variables are 'state' variables yet.

**Commented [HAN31]:** Should these be double-spaced as well?

**Commented [LDM32R31]:** No double spacing of figure legends.

**Commented [JKJ33]:** Very funky!
You should define the circles (nodes?) and lines (transitions?)

And, what do the size of the dots mean? What do the proximity to each other mean?

**Commented [LDM34R33]:** Authors Larson and Bungula heavily revised this figure legend to clarify.

The topological states algorithm further refined the two ecosystem states from the TDA Mapper outputs ~~classified 76,669 sites from the UMRS over 27 consecutive years~~ into five ecosystem states in a directed graph (Figure 1~~2~~1 ~~with colors~~).~~) by using 8 water quality variables (total nitrogen, total phosphorus, dissolved oxygen, chlorophyll *a*, water depth, temperature, velocity, and suspended solids). These five distinct states were refined and nested within the body and tail of the TDA structure; specifically,~~ States 1 and 2 were nested within the TDA Mapper body~~,~~ and States 3–5 were nested within the TDA Mapper tail. There were five, small transition nodes (meaning the sites were classified into both states simultaneously)~~,) in the TDA structure,~~ which lied between states 1 and 2 but couldn't be seen graphically in 2 dimensions because the nodes were small. Transition nodes were visible in a 3-dimensional TDA Mapper output, but we did not explore the transition nodes further due to rarity of transition nodes. States 1, 2, 3, 4, 5 contained 20%, 85%, 2%, <1%, and <1% of data across the six reaches and 27-year record, respectively.

**Commented [HAN35]:** Not sure if that explanation is necessary?

**Commented [LDM36R35]:** Accepted the deletion, 'with colors'

*What were the state variables that defined and distinguished riverine ecosystem states? Hypothesis 2*

State 1 may be considered a 'clear-water state' because this state had the lowest total phosphorus, highest dissolved oxygen, lowest chlorophyll *a*, coldest water temperatures, slowest water velocity, and lowest suspended solids relative to the other four states (Figure 2). State 1 was differentiated from State 2 principally by lower suspended solids (Figures 2f, 2h). State 2 contained most sites (Table 1; the so-called 'status-quo' state), and the state variables differentiating State 2 from others included total phosphorus, chlorophyll *a*, and suspended solids (in support of Hypothesis 2; Figure 2). State 2 had the greatest variability and many statistical outliers for all state variables. State 2 typically had lower nutrients but higher chlorophyll *a* relative to States 3, 4, and 5.

States 3, 4, 5 within the structure's tail resembled the 'turbid-state.' These three states were like each other for seven water quality variables (Figure 2a–2g) and differentiated from one another by the relative concentrations of suspended solids (Figure 2h). Compared to States 1 and 2, the States 3–5 had greater total phosphorus, lower dissolved oxygen, greater water depths and velocities, and orders of magnitude greater suspended solids. States 3, 4, and 5 also had higher water depths and velocity compared to States 1 and 2. Total nitrogen was the only variable that didn't vary in data distribution among the five state classifications (Figure 2a), so we determined total nitrogen was not a state variable.

**Table 1.** The distribution of sites in each upper Mississippi River ~~pool~~ reach (column 1: Pool name, field station city and state) that were classified into a topological/ecosystem state within years 1993–2019. Table 1a shows the ~~proportion~~ percentage (%) of sites in each state according to reach ~~river pool~~ for comparisons of ecosystem states *among the six*reaches~~*pools*.~~ (~~i.e.~~i.e., columns sum to 100%). Table 1b shows the ~~proportion~~ percentage (%) of sites classified within a reach~~pool~~ to compare the distribution of states *within each* reach~~*pool*~~. Sites sometimes can exist in more than one node, and therefore the percentage of sites within a state may sum to greater than 100%~~%~~ (i.e., rows in Table 1b sum ~~to~~greater than or equal to 100%).

**TABLE 1a**

| **River Reach ~~pool~~** | **State 1** | **State 2** | **State 3** | **State 4** | **State 5** |
|---|---|---|---|---|---|
| **Pool 4 (Lake City, MN)** | 31~~%~~ | 16~~%~~ | 0~~%~~ | 0~~%~~ | 0~~%~~ |
| **Pool 8 (Onalaska, WI)** | 35~~%~~ | 17~~%~~ | <1~~%~~ | 0~~%~~ | 0~~%~~ |
| **Pool 13 (Bellevue, IA)** | 26~~%~~ | 18~~%~~ | <1~~%~~ | 0~~%~~ | 0~~%~~ |
| **Pool 26 (Brighton, IL)** | 5~~%~~ | 16~~%~~ | 8~~%~~ | 5~~%~~ | 6~~%~~ |
| **Open River (Jackson, MO)** | <1~~%~~ | 17~~%~~ | 90~~%~~ | 95~~%~~ | 93~~%~~ |
| **La Grange** | 2~~%~~ | 16~~%~~ | <1~~%~~ | 0~~%~~ | <1~~%~~ |

**Formatted:** Font: Italic

**Formatted:** Font: Italic

**Formatted Table**

| | | | | | |
|---|---|---|---|---|---|
| **(Havana, IL)** | | | | | |
| ~~**Total sites**~~ | ~~**100%**~~ | ~~**100%**~~ | ~~**100%**~~ | ~~**100%**~~ | ~~**100%**~~ |

**TABLE 1b**

| **River Reach~~Pool~~** | **State 1** | **State 2** | **State 3** | **State 4** | **State 5** |
|---|---|---|---|---|---|
| **Pool 4 (Lake City, MN)** | 37 | 81 | 0 | 0 | 0 |
| **Pool 8 (Onalaska, WI)** | 39 | 78 | <1 | 0 | 0 |
| **Pool 13 (Bellevue, IA)** | 27 | 82 | <1 | 0 | 0 |
| **Pool 26 (Brighton, IL)** | 7 | 95 | <1 | <1 | <1 |
| **Open River (Jackson, MO)** | <1 | 87 | 9 | 3 | 1 |
| **La Grange (Havana, IL)** | 5 | 92 | <1 | 0 | <1 |

**Formatted Table**

**Commented [JKJ37]:** Missing % symbols?

**Commented [LDM38R37]:** Good catch.

We removed the % in Table 1A because its better reported in the figure legend text.

**Figure 2.** Graphical outputs from topological data analysis (TDA Mapper) showing ecological gradients of 8 water quality ~~state~~ variables in the Upper Mississippi River System, USA (*n*=69,307 sampling sites from 1993–2020). The color coding represents relative values for each variable; dark violet colors are relatively low values, whereas greens and yellows are relatively high values. The boxplots below each TDA Mapper graph showed the data distributions for each ecosystem state (that corresponds to the states in Figure 1). Boxplots are standardized by boxes (containing the $25^{th}$, $50^{th}$ (median), and $75^{th}$ percentiles), the whiskers (containing the ~~$0^{th}$~~ $25^{th}$ and ~~$100^{th}$~~ -$75^{th}$ percentiles~~, excluding outliers~~), and the dots (statistical outliers). The x-axis on the boxplots represents the five topological and possibly ecosystem states (States 1–5). The y-axis represents variable units (total nitrogen = mg L$^{-1}$; total phosphorus = mg L$^{-1}$; dissolved oxygen = mg L$^{-1}$; chlorophyll *a* = µg L$^{-1}$; water depth = cm; temperature = °C; velocity = m sec$^{-1}$; suspended solids = mg L$^{-1}$).

### *Did ~~pools~~ reaches exist in different ecosystem states? Hypothesis 3*

The six study ~~pools~~ reaches spanning 2200 km of river were classified into different ~~ecosystem~~ topological~~…~~ and ecosystem states (in support of Hypothesis 3). Different~~Evidence for the different~~ states ~~were shown by the highlighting~~were highlighted in different locations on the main TDA structure (Figure 3) and noted by the percentage of sites in each ~~pool~~ reach's state classification ~~classified to a state~~ (Table 1). Pool 4 (Minnesota), Pool 8 (Wisconsin), Pool 13 (Iowa), and La Grange Pool (Havana, Illinois; Illinois River) were within States 1 and 2 across the 27-year record. Therefore, Pools 4, 8, and 13 were principally categorized as within a 'status-quo state' (State 2) characterized as having a broad range of ecological conditions but~~that sometimes seasonally~~ often included the 'clear-water' state (27–39% sites within State 1 for these reaches). Pool 26 (Brighton, Illinois) existed in all five states, but State 2 was dominant~~dominate~~ (Table 1, Figure 3).~~ate.~~ Open River (Jackson, Missouri) was the principal ~~pool~~ reach within States 3-5. Open River was rarely in State 1, mostly within state 2, and 13% of sites were within the turbid-states of States 3–5.

**Figure 3.** Topological data analysis (TDA) showed spatial differences of water quality (8 variables; total nitrogen, total phosphorus, dissolved oxygen, chlorophyll *a*, water depth, water temperature, velocity, and suspended solids) among six ~~one primary data structure for 8 water quality state variables from six navigation pools~~ reaches on the Upper Mississippi River System

(years 1993–2020). The~~, and the~~ green colors highlighted where sampling sites within each ~~pool~~ reach grouped onto the primary TDA Mapper structure, and the differences indicated spatial differences among the six reaches. ~~navigation pools.~~ Dark green nodes had more~~relatively many~~ sites within ~~the~~ each node compared to the lighter green nodes. ~~containing fewer less sites. Different positions on these TDA Mapper graphs were highlighted in green according to pool~~~~reach, indicating these pools have belong to different ecosystem states based on water quality. Pools~~ Reaches can be classified into specific ecosystem states by referencing their positions within Figure 1~~2~~1; for example, ~~the~~ Pool 4 was within States 1 and 2 because the green highlights are on the left side of the TDA structure (panel a). As another example, Open River ~~pool~~ was principally within State 2–5 ~~(also see Table 1) States 3, 4, and 5~~ because the green highlights are on the right side of the TDA structure and its tail (panel f). ~~Pools~~ Reaches can also be characterized by water quality state variables in Figure 2~~3~~2; for example, Open River ~~pool~~ is typically characterized by high velocity and suspended solids.

*Did ~~pools~~ reaches undergo state transitions ~~or was there state stability over~~within the past 30 years? Hypothesis 4*

The six ~~pools~~ reaches varied substantially in their ecosystem state stability and the frequency of state transitions (Figure 4). Pool 26 and La Grange ~~Pool~~ were stable within State 2 and occasionally State 1 and did not undergo state transitions. Pools 4, 8 and 13 displayed cyclic dynamics between State 1 and State 2 that were recurring annually due to seasonal changes in water quality. State 1 dominated in winter of most years (S1 Figure) and State 2 dominated in the other three seasons (S1 Figure). State 1 was uncommon but possible outside of winter, and State 1 was rare in summer (indicated by low ~~correlation~~ proportion values of State 1 during summers). State 1 was seasonally dynamic as indicated by cyclic temporal proportion~~correlation~~ values that were greatest in winter, declined by spring and summer, and then increased by the following autumn and winter (Figure 4).

**Figure 4.** Change~~Temporal correlation function~~ detection plots showed the transitions in ecosystem states within six ~~pools~~ reaches of the Upper Mississippi River System from years 1993–2020. The ~~correlation~~ proportion value (y axes) is calculated as the proportion of sites classified within each state each season. ~~compared to the number of sites collected during that season (~125 sites per season per year per pool).~~

**Commented [JKJ39]:** Interesting! Cool how you see the reaches spatially transition across the graphs. And, Pool 26 and La Grange maps seem to be more variable than others

**Commented [LDM40R39]:** Indeed, thank you for the compliments. We revised the figure legend slightly for more clarifications.

**Commented [JKJ41]:** Neat to see the greater seasonality in the upper pools versus the lower pools in this plot

**Commented [LDM42R41]:** Thank you!

Occasionally, sites were classified into multiple states, and therefore the ~~correlation~~ proportion sum may exceed 1.0. ~~If correlation lines from States 1–5 never crossed in the time series, that pool had state stability and had not experienced state changes. If correlation lines crossed temporarily, this indicated instable and transient dynamics due to seasonal effects or episodic events. If a state had a monotonic decrease in correlation and then correlation lines crossed, this could have indicated a long-lasting state change or ecological ‘regime shift.’~~ Points that fall directly on the year were collected in winter, followed by spring, summer, and autumn samples. Data from 2003 was missing due to logistical constraints for collection.

**S1 Figure**. Graphical output from topological data analysis (TDA Mapper) showing a strong seasonal component to the water quality data for the Upper Mississippi River System, USA. Data inputs included 8 water quality variables collected four times each year from 1993–2020 at a total of 69,307 sampling sites. The graph is comprised of nodes (circles), which cluster sampling sites along the river based on water quality similarities, and the edges (lines) show statistical connections among nodes. We highlighted in color the seasons in which sites were sampled. Seasonality can be connected to ecosystem states by referencing Figure 1 positions; for example, winter is typically characterized by State 1.

**Commented [LDM43]:** Added since revisions

Pool 4 and Pool 8 gradually shifted towards State 1 through time.~~, albeit gradually.~~ The change ~~temporal correlation~~detection function did not detect an~~a dramatic~~ abrupt ~~ecological regime~~ state shift transition around years 2008–2010 in Pools 4 and 8 (refuting Hypothesis 4a). ~~, as indicated by a lack of monotonic decrease in correlations proportions between States 1 and 2 (Figure 4).~~ However, Pool 4 and Pool 8 had increasing minimum proportions ~~correlation~~ values through time for State 1, especially after year 2010 (Figure 4). The increasing proportions~~correlation~~ values indicated State 1 (a ‘clear-water’ state~~conditions~~ in winter) became more prevalent over time and thus ~~water quality conditions~~water clarity during winters improved in those two ~~pools~~ reaches since 1993 and especially since 2010 (in support of Hypothesis 4b).

Pool 13 showed different patterns in state transitions (Figure 4). The increased minimum ~~correlation~~ proportion values over time in State 1 were less pronounced than in Pools 4 and 8, indicating ~~less water quality improvements~~less increase in water clarity from reduced suspended solids. Since 2015 in Pool

13, the ~~expected~~ previous pattern of transitioning to State 1 in winter had become less common and predictable compared to previous years. State 3 (a turbid-state ~~with very high suspended solids~~) was first entered in 2017 and again in 2019 at some Pool 13 sites, indicating decreased~~declining water quality conditions~~ water clarity from ~~increasing~~ increased suspended solids.

Open River (Jackson, Missouri) transitioned between State 2 and State 3 ~~four times~~ sporadically four times over 27 years. Transitions to State 3 generally only lasted one or two seasons before returning to State 2. ~~– State 3 became predominate, which only lasted one or two seasons before returning to State 2.~~ The four state~~transitions between States 2 and 3~~ transitions in Open River reach occurred across various seasons and years (specifically, spring-summer 1999; winter-spring 2010; winter 2014; autumn 2015).

**Commented [JKJ44]:** That's interesting – more winter rain events?

**Commented [LDM45R44]:** Thanks. More winter rain events or lingering increases in SS over the previous seasons could be the cause. We tried to insert these spectulations in the Discussion, but it became too lengthy and specific to this system. Therefore, we don't include the possible reasons this happened and focus on how TDA can detect these changes.

**Formatted:** Font: (Default) +Body (Calibri)

**Formatted:** Font: +Body (Calibri), Font color: Black

**Commented [LDM46]:** Moved from below, as suggested by reviewer3

## DISCUSSION

This is the first study to use powerful topology tools of this type to quantify ecosystem states and state transitions in a~~landscapes and riverscapes~~a large ecosystem. Our topological analyses ~~successfully revealed multiple riverine states~~successfully detected patterns for multiple riverine states, the defining state variables, and differences among ~~pools~~ reaches in the Upper Mississippi River System (in support of our Hypotheses 1–3 and visualized in Figures 1–4). Our results expand the existing Mississippi River literature by using a multivariate approach to classify nearly 70,000 sites into multiple ecosystem states, identify water quality state variables, and explore state transitions. The TDA change detection analysis examined multivariate state transitions (Figure 4 and temporal proportion values), which is a powerful approach to thinking about multiple correlated variables and how they vary synchronously in space and time. The TDA detected short-term state changes based on seasonality and episodic events and provided evidence of gradual, long-term changes due to water quality improvements over three decades (Figure 4). We discuss how TDA may be used as a new technique for

**Commented [HAN47]:** This is up to you and your team–making a statement that a project is completely novel is usually challenged by reviewers/editors and you are free to make it, but writing it this way helps to account somewhat for the possibility that there's a study out there that you haven't seen or reviewed

**Commented [LDM48R47]:** I appreciate your suggestion and retain the insertion.

describing ecosystem states at multiple spatiotemporal scales, how the results will inform ecological restoration and management, and how the state concepts tested with TDA methods may be transferred to ~~any~~ other ecosystems that exists in one or more states.

*TDA as a novel technique for ecosystem states and transitions*

Testing ecosystem state concepts with TDA were inspired by past research of the human-gut microbiome (25,31), and our study shows TDA can also effectively identify ~~disentangle states~~ecosystem states and state variables from big data of large ecosystems. Our TDA results aligns well with previous studies of Upper Mississippi River System that ~~examined~~ described ~~broad ecological gradients~~sharp differences among ~~pools~~ reaches and water quality changes through time (2,19). The TDA results supported another author's previous ~~-~~hypothesis that the Upper Mississippi River System exists in two ~~alternate~~ alternative states: ~~egimes f clear-water~~status-quo (States 1 and 2) and turbid-states (States 3–5) as defined by state variables of ~~water clarity due to~~ suspended solids and chlorophyll *a* (18). ~~However,~~ State 2 had very high variability across all state variables that defines the status-quo state, meaning the water quality is highly variable across large spatiotemporal scales. ~~to be of practical use unless comparing directly to States 3–5.~~ Likewise, the main differentiation between the rare States 3–5 were based solely on suspended solid concentrations, which may be better represented as an ecological gradient than firm state classification via TDA. Although we could have altered the TDA density algorithm to explore more sub-states within these five states, we didn't have justification for refinement and wanted to provide a reasonable number of states that were clearly differentiated for aiding applied conservation (29).

The ecosystem states concepts, the TDA methods chosen to reflect ecosystem states, and the TDA results require some subjectivity in determining usefulness towards ecological understanding and management. For example, we suggested the TDA Mapper outputs of two alternative states (Figure 1)

**Commented [JKJ49]:** Does it? If there are five states? And two of them seem to mainly reflect seasonal variability? At what time scale does a state need to be "stable" to be defined as a "state"? Or is that not the expectation?

**Commented [LDM50R49]:** I carefully considered this comment.

I revised the first 2 results paragraph to better show there are two dominant states, shown as a body and tail in TDA Mapper. Next, a different algorithm refined TDA Mapper to include 5 states.

Throughout, I changed my subjective state names to 'status-quo' and 'turbid'. I didn't like naming States 1 and 2, which had 95% of the data, as 'clear-water.' The Mississippi is not clear most of the time! Status-quo was redefined in the Objectives statement.

The stability question inspired us to write another paragraph (Lines 283-291). on the admitted subjectivity required by the user for determining what is meaningful.

**Commented [HAN51]:** Interesting statement, can you briefly suggest future research that would help support the use of the two alternative states within this paragraph? What else do you think needs to be looked into in order to make the information more applicable to management?

**Commented [LDM52R51]:** Good suggestion. In the following section, "ecosystem states results can guide ecological restoration and management" we added new paragraphs to this.

and the TDA algorithm refining two states into five states (Figure 2) are both valuable to display and guide resource management across the Upper Mississippi River System. Users must decide at which ~~time-scale~~timescale a state is ‘stable.’ The change detection function we used statistically determined when states had transitioned, but the user must determine that those results signify expected seasonal transitions (Figure 4, S1 Figure), ‘abrupt regime shifts’ (not detected in this study), or unmeaningful noise. ~~Our results expand the existing knowledge by using a multivariate approach to classify nearly 70,000 sites into multiple ecosystem states, identify water quality state variables, and explore state transitions. The TDA trend analysis examined multivariate (state) transitions (Figure 5 and temporal correlation results), which is a powerful approach to thinking about multiple correlated variables and how they vary synchronously in space and time rather than assessments of single variables and their changes as in Houser (2022).~~

The TDA change detection function explored whether state transitions were dramatic and long-standing (i.e., an abrupt regime shift) or whether transitions were temporary state changes (i.e., transient dynamics) (Hypothesis 4). The TDA detected frequent transient dynamics based on seasonality and episodic state changes (Figure 4) but did not detect abrupt state changes with sharp break points in time. Future TDA analyses could include data on submersed aquatic plant biomass or species composition (32) because aquatic plants are likely another state variable (18) that may reveal abrupt shifts around 2010 in support of our Hypothesis 4. Other TDA tools like ‘persistence homology’ can detect specific change points (33), and thus may detect abrupt state transitions that are hypothesized to occur in rivers with long retention times (34) or following ecological restoration (18). Alternatively, the river’s ecology may be highly multidimensional and complex so that there was not a single point in time where major and abrupt changes happened, but rather changes were gradual over decades and most accurately portrayed with the change~~temporal correlation~~ detection function used herein (Figure 4). The lack of abrupt regime shifts in the Upper Mississippi River System would be different than is commonly

**Commented [JKJ53]:** I might avoid this statement.. as that’s not really an accurate characterization of the Status and Trends report which evaluated multiple indicator variables and includes multivariate analyses..

I would agree it’s most common in a management context to think about single variables, but there are multiple examples of water quality studies that do multivariate analyses or clustering approaches, so maybe rephrase!

**Commented [LDM54R53]:** Agreed. We will remove this statement entirely as suggested.

**Commented [LDM55]:** Moved to the first paragraph in the Discussion

observed within nearby shallow lake systems, which can transition to the alternative state within a few days and persist for years (8,9).

The TDA ~~also~~ allows extraction of information at finer spatiotemporal scales but was beyond the scope of this paper but could be explored in future work.~~.~~ For example, information from the data within the nodes can further describe how specific sites, seasons (S1 Figure), riverine habitat strata, and years are classified within each state to further ecological understanding and prioritize ecological restorations. Sites classified within multiple states, transition nodes, or the TDA outliers could be further explored for unusual site characteristics and assessing site-level variability that affects ecosystem state.

### *Ecosystem states results can guide ecological management & restoration*

Coupling ecosystem state theories with scientific models can provide urgently needed guidance for ecological restoration and management (35,36). Previously applied uses of state theory included predicting pending state transitions (37–39), managing systems for resilience and preventing undesirable transitions (38), and managing state variables to restore or protect resilience of desirable ecosystem states (9,40). However, state classification and state transition models are rarely used in large monitoring programs because of modeling complexities and the inability of institutions to practically apply model outputs (3).

We used big data (nearly 70,000 sites and 8 variables) from long-term river monitoring (41), and these TDA results can guide structured decision making~~, adaptive management,~~ and vulnerability assessments in the Upper Mississippi River System. This is the first study in this large watershed to classify ecosystem states, which is foundational for describing the system's ecological structures and functions. Having defined ecosystem states allows practitioners to communicate which states are desirable for preservation and which ~~regimes~~ are undesirable for mitigation, as initiated in (18,29,42). We identified the state variables (specifically total phosphorus, suspended solids, and chlorophyll *a*),

which can guide decision making and actions for regulatory and restoration agencies by providing quantitative targets. Currently, specific regional water quality criteria are recommended for total phosphorus and chlorophyll *a*, but not suspended solids (43). Specified ~~There remains a lack of a~~ ~~s~~targets for suspended solids concentrations are lacking, although water clarity remains a common restoration goal (29), and our TDA results echo the importance of suspended solids a state variable.

The TDA's change~~correlation function~~ detection can be a novel tool for assessing state transitions and vulnerability as new, long-term data are gathered. This function effectively signaled state changes in the Upper Mississippi River System due to seasonality, episodic events, and gradual changes to water quality (Figure 4). The state transition modeling in this system can continue to reveal gradual improvements to water quality and signal vulnerability to undesirable state transitions if States 3–5 become more prominent in certain reaches, like Pool 13 (Figure 4). ~~The correlation function gave an early warning signal of imminent state transitions in Pool 13.~~ The TDA tool detected changes to the variability of the number of sites within a state as the proportion values, and a change in time series variability can be an early warning indicator of ecological transitions (44)~~indicated Pool 13 may be vulnerable to transitioning to the undesirable turbid-state (State 3; Figure 4) because it has transitioned to State 3 repeatedly only in the last few years of a 30-year record and the high suspended solid concentrations characteristic of State 3 are undesirable in this system (McCain et al. 2018).~~ The TDA could detect if more sites within Pool 13 continue to enter State 3 more frequently or persistently in the ~~near future~~near future. The state variables of total phosphorus, chlorophyll *a*, and suspended solids identified for the Mississippi River are the same as those of lake ecosystems (8), and so assessing vulnerability to state transitions using these variables could be a warning in other aquatic systems beyond the Mississippi River. Lastly, the states could become a categorical response variable in future models that aim to assess the environmental drivers and threshold responses, although researchers

**Formatted:** Font: Italic

should carefully consider whether such information is better addressed with gradient analyses or state classification (45).

*Transferability of TDA to other ecosystems*

Ecologists have emphasized the need to apply ecosystem states concepts to rivers worldwide, while acknowledging most rivers do not have sufficient data to properly evaluate states (2,7,18,23). The Mississippi River has exceptional long-term data, and our results supported the concepts of alternative states in this particular river (18). We suspect that similar ecosystem states based on water quality state variables can be found in other rivers, although we are unaware of other case studies to-date other than 'clear-water' and 'turbid-states' based on aquatic plant abundance and water quality state variables (23). The Regime Shifts Database (7) could serve as a data source to test our TDA ecosystem states framework in other rivers with similar water quality data (e.g., known hypoxia regime shifts within the Amite River, Brazo River, Chester River, and Indian River in the USA). For ecosystems without the big data like the Mississippi River, monitoring system status and trends (like state transition warnings) could focus on the three state variables in aquatic systems: total phosphorus, chlorophyll a, and suspended solids. Transferability of the TDA approach (outlined in Figure 5) will be limited to ecosystems with sufficient data, although the number of sites and variables required for ~~is not well known~~useful ecological assessments with TDA are not yet known.

Overall, TDA was a novel technique that effectively addressed many hypotheses regarding the Upper Mississippi River System as characterized by predictable ecosystem states, a few state variables, and state transitions. The TDA may provide an early warning indication of vulnerability to undesirable ecosystem transitions, such as Pool 13's increasingly frequent transitions to the undesirable state 3 with high suspended sediment loading. Testing ecosystem states in freshwater systems (beyond well-studied shallow lakes) is important, while being cognizant to the limitations and misuses of the theories (45).

The TDA is advantageous in that if spatiotemporal data are available, then the model outputs can be derived for a variety of spatial scales (e.g., site-, habitat-, reach~~poo~~-, and systemic-levels) and many temporal scales (seasonal- to multi-decadal) to match management objectives and a manager's geographical jurisdiction. Selection of state variables for TDA should be ecologically meaningful and allow managers to influence those variables to advance conservation (46). The novel coupling of ecosystem state concepts with multiple~~and TDA methodologies~~ TDA tools revealed new information about the ecosystem states and ~~can be transferred to any ecosystem to help classify states and predict their vulnerability to~~ state transitions of the Mississippi River, and this approach (Figure 5) may~~.~~ ~~can~~ be transferred to any ecosystem with big~~large~~ data to help classify states and understand their vulnerability to state transitions.

## ~~MATERIALS AND~~ METHODS

### *Study Area, Ecosystem State Context, and Long-term Data*

The ~~upper~~ Upper Mississippi River System flows ~2200 km from Minneapolis, MN to the confluence with the Ohio River in Cairo, IL and includes the navigable Illinois River (2). The~~across the USA and its~~ UMRS has 29 locks and dams have created impounded areas~~'pools'~~ upriver of each ~~impoundment.~~lock and dam to maintain a deep main channel for barge traffic, but the locks and dams do not modify water residence time or stop flooding. Lock and dams were installed in the late 1930's~~.~~ which has greatly modified the river's physical and hydrological template~~.~~. Aquatic plants were abundant after lock and dam construction and then were abruptly ~~lost~~ reduced in the late 1980's for reasons unknown. Aquatic plants gradually returned and ~~has~~ remained ~~resilient~~ abundant since 2010 even under extended flood conditions from 2017–2019 (30). ~~.~~ We studied six navigable river ~~tionpools (herein called 'pools' for brevity)~~reaches named Pool 4, Pool 8, Pool 13, Pool 26, Pool La Grange ~~(Illinois River)~~ , and Open River that span multiple environmental gradients like geomorphology, hydrology, and water quality (2)~~.~~

**Commented [JKJ56]:** Depending on the data that are available? LTRM has all data at all of those scales, how would you handle it if another river system didn't?

**Commented [LDM57R56]:** Great point. Added: ". The TDA is advantageous in that if spatiotemporal data are available, then the model outputs can be derived for a variety of spatial scales (e.g., site-, habitat-, pool-, and systemic-levels) and temporal scales (seasonal- to multi-decadal)…"

**Commented [JKJ58]:** If you're including the La Grange reach, technically should be the "Upper Mississippi River System", so maybe add in additional information about the IL River here?

**Commented [LDM59R58]:** Great. We added, Upper Mississippi River System, mention the Illinois River, and cite Houser 2022.

**Commented [JKJ60]:** Worth including a statement that locks and dams don't modify residence time – they modify the river, but not in the way that a hydro dam does and it seems people often assume they do (who don't know the river!)

**Commented [LDM61R60]:** Good point! We added this on lines 460-462.

~~(Houser 2022).~~ The study ~~area~~ reaches ~~was~~ were a high restoration priority and had long-term data on water quality.

We assessed ecosystem states based on water quality data at 69,307 sampling sites.~~sitesacross the six pools~~. Data were ~~(~~collected four times annually~~,~~ (over a two-week sampling episode) in each season from ~~years~~ 1993–2020 across six study reaches.~~pools~~ ~~),which~~ This is a 27-year consecutive record, except~~.~~, ~~but~~except no sampling was done in 2003.~~ due to funding constraints~~. The water quality data ~~was~~ were collected ~~by~~ using a ~~long-term,~~ standardized protocol whereby sites were selected by a stratified random sampling design with~~and~~ effort distributed proportionally ~~and~~ across strata. Strata were ~~were~~ defined based on enduring geomorphic features and included ~~which include~~ ~~habitat types like~~ the main channel ~~borders~~, contiguous backwaters, impounded areas above the lock and dams, and lotic side channels (47).~~(see Soballe and Fischer 2004 for full description of sampling design)~~

Using ecological literature and *a priori* hypotheses (as ~~(hypotheses 1-4b~~as stated in the Introduction), we chose eight water quality variables thought to be important for defining ecosystem states and ensured the variables were not highly correlated (Pearson's $r < 0.45$). The 8 variables included: total nitrogen = mg L$^{-1}$; total phosphorus = mg L$^{-1}$; dissolved oxygen = mg L$^{-1}$; chlorophyll *a* = µg L$^{-1}$; water depth = cm; temperature = °C; velocity = m sec$^{-1}$; suspended solids = mg L$^{-1}$. Although the TDA can incorporate more variables, we carefully selected 8 variables based on ecological relevance to state theory and the ability to manipulate these variables for restoration (e.g., water velocity and nutrient inputs may be manipulated; see (29).

### *Quantifying ecosystem states and the state variables*

To quantify the ecosystem states, we used several topological data analysis tools (Figure 5), which are dimension reduction techniques that interpret complex data (like multi-faceted water quality data, like ours herein) using the data's geometric and topological features. All data have underlaying

**Commented [JKJ62]:** Cite Status and Trends here?

**Commented [LDM63R62]:** Will do!

**Commented [JKJ64]:** Missing 2003…

**Commented [LDM65R64]:** Thanks. We retain your added phrase: "…but no sampling was done in 2003 due to funding constraints."

**Commented [JKJ66]:** What were you're a priori hypotheses?

**Commented [LDM67R66]:** Reference hypotheses 1-4b in Introduction

shape, and this can be visualized and interpreted with TDA to find hidden properties (e.g., ecosystem states and state variables), recognize complex spatial patterns (e.g., how ecosystems may differ across space), and classify large data into meaningful clusters (e.g., ecosystem states based on topological states). Wedoes X, Y, Z… w performed fivefour main tasks (Figure 5): 1.) data pre-processing, 2.) developed a novel? Applied the?applied the undirected, TDA Mapper algorithm, 3.) performed a density-based state assignment and created a directed TDA Mapper graph-, 4.) identifyingidentifiedying state variables, and 5.) Temporal Correlation created a change detection functionfFunction to detect state transitions over 27 years.

**Figure 5.** Flow chart that summarized our topological data analysis (TDA) methods in sequential order (arrows and gray text) and the outputted products (boxes with black text and images) for quantifying ecological states and state transitions of the Upper Mississippi River System. The original- and TDA-ready datasets and all analysis codes are available here (DOI: 10.5066/P976O6PPXXXX). In the "Undirected TDA Mapper" box, nodes and edges are connected to create a single TDA Mapper structure. In In "the "Directed TDA Mapper" box, the *example output* describes two local maxima nodes defining two states: a red state containing two nodes and a blue state containing one node. The grey node is a transition node and thedoes not belong tothe sites within the transition nodes are classified into both the red and blue state. In the "change detection" box, we provide a *hypothetical output* of where the dominance of two states (red and blue dashed lines, which are the proportion of sites within those states) temporarily transition from the dominant red state to the blue state around years 13 to 17.

1. Data Pre-processing

The original water quality dataset (accessed 06/01/2021 from https://umesc.usgs.gov/data_library/water_quality/water1_query.shtml), had 204,345 rows (site data) and 133 columns (variables), and contained data from 1993 to 2020, except 2003. We filtered the data by stratified random sampling scheme (and filtered out 'fixed sample sites', which contained ~60% of the data set), surface water collections, and selected 8 continuous variables (because TDA cannot effectively handle binary or ordinal variables). Negative values, which represent readings below the



**Commented [JKJ68]:** Continuous makes me think of sensors..

**Commented [LDM69R68]:** We retain continuous because TDA cannot handle binary and ordinal data, but added that.

minimum detection limit and was <0.2% of the data, were set to the minimum value of that variable in the entire dataset, and quality flag (QF) codes of ‘0’, ‘A’, ‘8’, or ‘64’ were set to ‘NA.’ ~~Duplicate site identifiers were combined with the arithmetic mean value replacing the existing column values.~~ After we removed three unreasonable and extreme outliers (TN > 25 mg/L), the final data size was 69,307 rows by 8 columns. Three of the variables (total nitrogen, total phosphorus and velocity), per the study design, were only collected at two thirds of the sampling sites.~~The three variables with ~66% of the values per the study design were total nitrogen, total phosphorus, and water velocity.~~ The TDA does not allow missing values, therefore ~~to To address missingness,~~ we compared ~~seven~~ multiple interpolation methods including kriging, inverse distance weighting of various distance thresholds, polynomial regression of various degrees, regression trees, and random forests. ~~and utilized~~We chose a random forests algorithm as the top ~~preforming~~ performing interpolation method (when compared to the mean absolute error to the other methods), which ~~that~~ had ~~greater than~~> 93% accuracy for all interpolated variables~~.~~ ~~(Larson et al. in review).~~ The other occasional missing values were imputed with the median value for each variable. ~~Two water clarity variables, turbidity and secchi disk depth, were not used because they were highly correlated with suspended solids.~~ We used robust scaling so each variable’s differing measurement units were accounted for in the multivariate analysis, and robust scaling calculates the distance between input values using the median and interquartile range.

2. The TDA Mapper Algorithm & Topological States Definition

The Topological Data Analysis (TDA) Mapper algorithm visualizes high dimensional data by incorporating dimension reduction techniques and user-selected clustering algorithms ~~–~~to classify sites into topological states (48). We suggest that topological states may indicate ecosystem states if the input variables are ecologically relevant and state variables are later revealed to classify sites within the TDA structure. The TDA Mapper algorithm takes in point cloud data and produces a ‘simplicial complex’, which is undirected graph that~~showing a TDA structure from a collection~~ shows data-shape based on ~~of~~

**Commented [JKJ70]:** Those negative values represent the negative of the minimum detection limit of the particular parameter

**Commented [LDM71R70]:** Added this comment to text

**Commented [JKJ72]:** So these were spatial and temporal estimates of missing data? Very cool you were able to do that. but maybe give a bit more description on how since you were filling 33% of the dataset…

**Commented [LDM73R72]:** Yeah, it’s powerful. We added that random forests had <93% prediction accuracy.

**Commented [LDM74]:** Response to PLOS reviewer 1

**Commented [JKJ75]:** What is robust scaling?? Is that a type of scaling metric?

**Commented [LDM76R75]:** Yes. We added: “We used robust scaling so each variable’s differing measurement units were accounted for in the multivariate analysis, and robust scaling calculates the distance between input values using the median and interquartile range.”

**Commented [JKJ77]:** If you’re considering “topological states” to be equivalent to “ecological states” I think a more clearly worded definition of what a topological state is and how it’s defined would go a long ways..

**Commented [LDM78R77]:** We added: “The Topological Data Analysis (TDA) Mapper algorithm visualizes high dimensional data by incorporating dimension reduction techniques and user-selected clustering algorithms (Singh et al. 2007) to classify sites into topological states. We suggest that topological states may indicate ecosystem states if the input variables are ecologically relevant and ecosystem state variables are later revealed to classify sites within the TDA structure.’

**Commented [LDM79R77]:** WAKO, do you like this?

**Commented [LDM80R77]:** Wako and I agreed on these revisions at an in-person on 2/14/23 and 2/16/23.

**Commented [JKJ81]:** Does it have its own clustering algorithms? Or do you specify one?

**Commented [LDM82R81]:** Clarified, ‘user-selected algorithms’

many nodes and edges (Figure 5). The nodes are clusters of sites that are similar based on the data (and the number of sites within each node can vary), and the edges signify interactions or connections among nodes. Nodes that are farther apart in ~~2 dimensional~~2-dimensional space indicate the sites are quite different. Essentially, the undirected TDA Mapper creates a single shape of all the data, consisting of nodes and edges. We used Keppler Mapper (a TDA Mapper) written in Python (van Veen et al. 2019) on the TDA-ready data set. ~~Next, we use a topological states algorithm to refine the simplicial complex (i.e., the undirected TDA Mapper output) into a directed TDA Mapper graph, which further refines the data's shape into topological states.~~ The TDA Mapper algorithm was fromm~~and topological state algorithm includ~~ (48)~~, Chang et al. 2020):~~.

~~[TDA Mapper algorithm]~~

~~Input: X, input dataset of continuous variables~~
~~Filter X using *f* : X → Z, where dim(Z) < dim(X)~~
~~Cover *f* with a finite cover U~~
~~Obtain $f^{-1}$(U), a finite cover of X~~
~~Apply DBSCAN on the overlapping bins~~
~~Output: a simplicial complex (undirected graph) S, where nodes represent clusters and edges represent non-empty intersection between two nodes.~~

~~[Topological State definition & algorithm]~~

~~Input: Undirected Mapper graph, S~~
~~Compute density of each node of S~~
~~Output: Create a directed graph G by creating directed edges from lower density nodes to higher density nodes~~
~~Determine number of topological states by number of local maxima~~
~~Assign each site to the state associated with the nearest local maxima~~

The parameters of the TDA Mapper algorithm determine the shape of the data and are chosen by the user. The TDA Mapper parameters include filter function, size of the hypercubes, percent overlap, and clustering algorithm. As of 2022, there were not established methods for selecting ~~values for these parameters?parameters~~these TDA parameter values of our interest with the TDA Mapper, except see (49), so we explored multiple parameters and observed their effects on the simplicial complex. The TDA

**Commented [JKJ83]:** What are nodes and edges? What do they signify?

**Commented [LDM84R83]:** We added: "The nodes are clusters of sites that are similar based on the data, and the edges signify interactions or connections among nodes." We also added this to Figure 1 legend for help with interpretation.

**Formatted:** Underline

**Commented [JKJ85]:** I think you need to define these variables (what X, Z, U..)

As worded, this doesn't really help me understand what the algorithm is doing – would people who know the technique need to see this?

**Commented [LDM86R85]:** This algorithm is not really needed here. We explain the process in detail and cite the original paper, so we deleted. Thanks!

**Commented [LDM87]:** Moved below

**Formatted Table**

**Commented [JKJ88]:** What do those parameters do?

**Commented [LDM89R88]:** Added: "The parameters of the TDA Mapper algorithm determine the shape of the data, and are chosen by…"

Mapper graphs using different parameters should be consistent and insensitive to relatively small changes in parameter values. To ensure the shape of the TDA graph persists for various parameter choices ~~near the values stated above~~, we ran the TDA Mapper for many combinations of these parameter values: cube size ([219,100], [273.75, 125], [328.5, 150], [383.25, 175], [438, 200]) and percent overlap (30%, 40%, and 50%). The~~As expected, the~~ TDA Mapper graphs persisted through these parameter ~~changes~~ combinations and verified our final parameter selections were appropriate and that the TDA Mapper algorithm captured the true shape of the underlying data space.

The final parameters we chose included a principal component analysis as the filter function, the cube size of [273, 125], and DBSCAN as the clustering algorithm with the following justifications. The first two principal components (as determined from principal component analysis, PCA) were used as the filter function. We used the following heuristics to determine the optimal cube size and percent overlap parameters like (25): each node must contain no more than 10% of the data~~-~~, and at least 90% of the data must be used in the main TDA Mapper structure that includes the body, tail, and outliers. In other words, at least 90% of the data must be used to create the TDA Mapper and up to 10% of data could be acceptably lost as 'noise.' We quantified and reported in the results the amount of data expressed in the final TDA Mapper output.~~and at least 90% of the data must be used in the main structure.~~ We used the ratio between the explained variances of the first two PCA components of our filter function as the ratio of the components in the cube size parameter. ~~Since the explained variances of the first two PCA components were [0.427, 0.195], we~~ We used the cube size of [273, 125]~~].~~ ~~, since~~ because the PCA with those values explained the most variance. We also used 50% overlap, which was not a sensitive parameter when exploring 40–60% overlaps. For our clustering algorithm, we chose the default parameter 'DBSCAN' within Keppler Mapper. To determine DBSCAN's optimal minimum samples and epsilon parameters, we used the Elbow method that uses a minimum samples parameter of two times the number of data attributes. The epsilon values are selected by the following process:

**Commented [JKJ90]:** PCA of what? And why was it done?

**Commented [LDM91R90]:** The PCA is the approach chosen for the filter function parameter.

**Commented [LDM92R90]:** Wako and I agreed on these revisions at an in-person on 2/14/23 and 2/16/23.

1. Finding the average distance of each data points k-nearest neighbors (kNN)
2. Sorting and plotting the averages kNN distances for all data points
3. Finding the point of greatest curvature. Since eight water quality variables were used to produce the simplicial complex, we used 16 as our minimum samples. The epsilon value used was 1.5 and calculated using the Kneed package (Satopa et al. 2022).

3. Density-based state assignment

Next, we use a topological states algorithm to refine the simplicial complex (i.e., the undirected TDA Mapper output) into a directed TDA Mapper graph, which further refines the data's shape into more topological states (Figure 5). ~~-~~We applied a density algorithm to the undirected TDA Mapper graph to produce a directed graph with defined states as adopted and modified from (25). We used k-nearest neighbor (kNN) distance. Each node is assigned a density of

$$D(V) = \frac{n^2}{\sum_{i \in V} kNN(i,k)}$$

~~$$D(V) = \frac{n^2}{\sum_{i \in V} kNN(i,k)}$$~~

, where $n^2$ counts for differing node sizes and $kNN(i,k)$ is the kNN distance of point $i$ defined as

$$kNN(i,k) = \frac{\sum_{j \in N(k)_i} d_{ij}}{k}$$

~~$$kNN(i,k) = \frac{\sum_{j \in N(k)_i} d_{ij}}{k}$$~~

where $N(k)_i$ is the set of $k$ nearest points to the point $i, k$ is 10% of the number of samples in the dataset, and $d_{ij}$ is the distance between points $i$ and $j$. This density assignment allows converting the undirected TDA Mapper graph to a directed graph by replacing the edge between two nodes by an arrow pointing from the node with smaller density to the node with larger density. The "Topological state definitions~~State Definition~~" in algorithm ~~1~~ 2 and Figure 5 shows how a Mapper graph is converted to a directed graph to quantify local maxima nodes.



**Commented [JKJ93]:** Some parameters not showing in equation?

**Commented [LDM94R93]:** The parameters are included in this equation, but there was some strange dashing with no meaning. We replaced with the correct formulas.

**Commented [JKJ95]:** What's the difference between a directed and undirected graph? Why do you do both?

**Commented [LDM96R95]:** Earlier in this section (lines 393-402) and Figure 5 roadmap, we have added more about directed and undirected graphs.

The directed graph is grouped into topological states (and conceptually considered ecosystem states herein), and these states are determined by *local maxima* nodes towards which every arrow connected to them points (Figure 5). In other words, the number of local maxima nodes is equal to the number of states detected. The distance between a node and a local maxima node is defined to be the smallest possible number of edges connecting them. A single water quality sampling site may sometimes be placed into more than one node, and thus classified in more than one state, if two nodes in different states have an edge between them and intersection of points. If a node is equidistant from two or more local maxima, that node is called a 'transition node' and is assigned to both local maxima (i.e., classified in both states). Sampling sites with more than one state classification or in transition nodes represented sites that are not easily classified by the topological states definition.

3.4. Identifying state variables

A state variable is statistically considered athea variable most positively associated with data clustering into a given topological state and the variable's data distribution is different from that of other states.. 2. We included eight water quality variables for the TDA to capture the multidimensionality of ecosystem state concepts. To determine state variables, we examined the gradients of each water quality variable's data ranges displayed on the TDA Mapper structure in relation to where the state was defined on the TDA Mapper graph that classified states. We also compared the data distributions among the five state classifications using boxplots for each variable. If the median (50$^{th}$ percentile) and boxplot whiskers (25$^{th}$–75$^{th}$ percentiles) did not overlap among the states, we considered that a likely state variable. We did not run formal statistical analysis for 'statistical significance' among the five boxplots (e.g., analysis of variance among the states) because the high large number of data points ($n$=6976,66969,307) and degrees of freedom precluded that option due to risk of Type 1 error (i.e., false positive).

*Quantifying ecosystem state transitions*

~~4.~~5. Change ~~Temporal Correlation Functi~~detection function

We tested whether states were stable or transitioned over 27 years. We analyzed the state composition of each ~~pool~~ reach during each season (autumn, winter, spring, and summer). On average, each season-reach combination contained 122 datapoints. We defined~~(see Soballe and Fischer 2004 for variation in sample size by strata and pool). We definedtemporal correlation function~~ 'proportion values' for a ~~pool~~reach-season-state pair as:

$$f(x_{state}) = \frac{x_{state}}{n}$$

where $x_{state}$ is the number of sites within a reach ~~collected~~ classified within a particular state during that season, and $n$ is the total number of sites in any state collected during that reach-season combination. ~~In other words, the temporal correlation function is the proportion of sites in that state compared to the total number of sites collected during that season.~~ A hypothetical example showing a~~one~~a single state transition between two states based on the temporal changes in proportion values is ~~found~~ in Figure 5.

**Commented [JKJ97]:** I'm not sure I understand how this is "temporal correlation function" - are you then evaluating how that changes by season? For a given season across years?

**Commented [LDM98R97]:** We redefined as: "We defined a temporal correlation function for a pool-state pair as:

$$f(x_{state}) = \frac{x_{state}}{n}$$

where $x_{state}$ is the number of sites that lie within nodes of that state during that season, and $n$ is the total number of sites in any state collected during that season."

## ACKNOWLEDGMENTS

The data collection, data analysis, manuscript preparations,~~This work and~~ and authors DL and WB were funded as part of the U.S. Army Corps of Engineers' Upper Mississippi River Restoration Program (https://umesc.usgs.gov/ltrm-home.html). The National Science Foundation's "Research Experience for Undergraduates" (REU) Program Award Number:1559663 funded authors CM, AS, AL, KD, FM for data analysis and manuscript preparation. ~~(https://beta.nsf.gov/funding/opportunities/research-experiences-undergraduates-reu).~~ This research

was performed in part using computational resources supported by the Academic & Research Computing group at Worcester Polytechnic Institute.  We sincerely thank Gregory Sandland, James Pierce, Roger Haro, and Richard Erickson for REU Program leadership and support in 2021. We thank Douglas Krouth (UWL) for initiating the first TDA Mappers. We thank four anonymous reviewers for greatly improving this manuscript. Any use of trade, firm, or product names is for descriptive purposes only and does not imply endorsement by the U.S. Government.

**REFERENCES**

1. Dodds WK, Whiles MR. Freshwater Ecology: Concepts and Environmental Applications of Limnology. 3rd ed. Academic Press; 2019.

2. Houser J, Bouska K, De Jager N, Larson D, Ickes B, Jankowski K, et al. Ecological Status and Trends of the Upper Mississippi and Illinois Rivers. U.S. Geological Sur-vey Open-File Report 2022–1039; 2022. 1–218 p.

3. Bestelmeyer B, Ash A, Brown JR, Densambuu B, Fernández-Giménez M, Johanson J, et al. State and transition models: theory, applications, and challenges. In: Briske D, editor. Rangeland Systems: Processes, Management and Challenges [Internet]. Cham: Springer; 2017. p. 303–45. Available from: http://www.springer.com/series/412

4. Gunderson L, Holling C. Panarchy: understanding transformations in systems of humans and nature. Washington D.C.: Island Press; 2002.

5. Carpenter S, Walker B, Anderies JM, Abel N. From Metaphor to Measurement: Resilience of What to What? In: Ecosystems. 2001. p. 765–81.

6. Suding KN, Gross KL, Houseman GR. Alternative states and positive feedbacks in restoration ecology. Trends Ecol Evol. 2004;19(1):46–53.

7. Biggs R, Peterson GD, Rocha JC. The regime shifts database: A framework for analyzing regime shifts in social-ecological systems. Ecol Soc. 2018 Oct 1;23(3):018473.

8. Scheffer M. Ecology of shallow lakes. 1st ed. London: Kluwer Academic Publishers; 1993.

9. Larson DM, Cordts SD, Hansel-Welch N. Shallow lake management enhanced habitat and attracted waterbirds during fall migration. Hydrobiologia. 2020;846(16):3364–79.

10. Steneck RS, Graham MH, Bourque BJ, Corbett D, Erlandson JM, Estes JA, et al. Kelp forest ecosystems: biodiversity, stability, resilience and future. Environ Conserv [Internet]. 2002;29(4):436–59. Available from: https://about.jstor.org/terms

11. Bestelmeyer BT, Okin GS, Duniway MC, Archer SR, Sayre NF, Williamson JC, et al. Desertification, land use, and the transformation of global drylands. Front Ecol Environ. 2015 Feb 1;13(1):28–36.

12. Briggs JM, Blair JM, Mccarron J. An ecosystem in transition:cause and consequences of the conversion of mesic grassland to shrubland. Bioscience. 2005;55(7):561–72.

13. Mumby PJ, Hastings A, Edwards HJ. Thresholds and the resilience of Caribbean coral reefs. Nature. 2007 Nov 1;450(7166):98–101.

14. Larson DM, Dodds WK, Veach AM. Removal of Woody Riparian Vegetation Substantially Altered a Stream Ecosystem in an Otherwise Undisturbed Grassland Watershed. Ecosystems. 2019;22(1):64–76.

15. Zinnert JC, Nippert JB, Rudgers JA, Pennings SC, González G, Alber M, et al. State changes: insights from the U.S. Long Term Ecological Research Network. Ecosphere. 2021 May 1;12(5).

16. Martin BE, Walsh JR, Vander Zanden MJ. Rise of a native apex predator and an invasive zooplankton cause successive ecological regime shifts in a North Temperate Lake. Limnol Oceanogr. 2022 Feb 1;67(S1):S163–72.

17. Dent CL, Cumming GS, Carpenter SR. Multiple states in river and lake ecosystems. Philos Trans R Soc B Biol Sci. 2002 May 29;357(1421):635–45.

18. Bouska KL, Houser JN, De Jager NR, Drake DC, Collins SF, Gibson-Reinemer DK, et al. Conceptualizing alternate regimes in a large floodplain-river ecosystem: Water clarity, invasive fish, and floodplain vegetation. J Environ Manage [Internet]. 2020 Jun;264:110516. Available from: https://linkinghub.elsevier.com/retrieve/pii/S0301479720304503

19. Burdis RM, DeLain SA, Lund EM, Moore MJC, Popp WA. Decadal trends and ecological shifts in backwater lakes of a large floodplain river: Upper Mississippi River. Aquat Sci. 2020 Apr 1;82(2):1–19.

20. Shang J, Zhang W, Chen X, Li Y, Niu L, Wang L, et al. How environmental stress leads to alternative microbiota states in a river ecosystem: A new insight into river restoration. Water Res. 2021 Sep 15;203:1–9.

21. Larson DM, Carhart A, Lund E. Aquatic vegetation types identified during early and late phases of vegetation recovery in the Upper Mississippi River, in press. Ecosphere. 2023;

22. Diamond JS, Moatar F, Cohen MJ, Poirel A, Martinet C, Maire A, et al. Metabolic regime shifts and ecosystem state changes are decoupled in a large river. Limnol Oceanogr. 2022 Feb 1;67(S1):S54–70.

23. Ibanez C, Penuelas J. Changing nutrients changing rivers. Science (80- ). 2019 Aug 16;365(6454):637–8.

24. Dale MRT. Applying Graph Theory in Ecological Research. 1st ed. Cambridge: Cambridge University Press; 2017.

25. Chang WK, VanInsberghe D, Kelly L. Topological analysis reveals state transitions in human gut and marine bacterial communities. npj Biofilms Microbiomes. 2020 Dec 1;6(1):1–10.

26. Bendick R, Hoylman ZH. Topological data analysis reveals parameters with prognostic skill for extreme wildfire size. Environ Res Lett. 2020 Oct 1;15(10).

27. Yuvaraj M, Dey AK, Lyubchich V, Gel YR, Poor VH. Topological clustering of multilayer networks. Proc Natl Acad Sci. 2021;118(21):1–9.

28. Blevins AS. Extending topological data analysis in biological systems. University of Pennsylvania; 2019.

29. McCain K, Schmueker S, De Jager N. Habitat Needs Assessment - II : Linking Science to Management Perspectives. Rock Island, IL: U.S. Army Corps of Engineers; 2018.

30. Larson DM, Lund E, Carhart A, Drake D, Houser J, Bales K, et al. Status and Trends of Upper Mississippi River's Aquatic Vegetation from 1998 to 2019. In: Houser J, Bouska K, De Jager N, Ickes B, Jankowski KJ, Larson D, et al., editors. Status and Trends of the Upper Mississippi and Illinois Rivers. 3rd ed. U.S. Geological Sur-vey Open-File Report 2022–1039; 2022. p. 1–218.

31. Greenblum S, Turnbaugh PJ, Borenstein E. Metagenomic systems biology of the human gut microbiome reveals topological shifts associated with obesity and inflammatory bowel disease. Proc Natl Acad Sci U S A. 2012 Jan 10;109(2):594–9.

32. Mason TJ, Keith DA, Letten AD. Detecting state changes for ecosystem conservation with long-term monitoring of species composition: Ecol Appl. 2017 Mar 1;27(2):458–68.

33. Islambekov U, Yuvaraj M, Gel YR. Harnessing the power of topological data analysis to detect change points. In: Environmetrics. John Wiley and Sons Ltd; 2020.

34. Hilt S, Köhler J, Kozerski HP, van Nes EH, Scheffer M. Abrupt regime shifts in space and time along rivers and connected lake systems. Oikos. 2011 May;120(5):766–75.

35. Scheffer M, Bascompte J, Brock W, Brovkin V. Early-warning signals for critical transitions. Nature [Internet]. 2009 [cited 2017 Aug 21];461(7260):53–9. Available from: http://search.proquest.com/openview/7734e5b77c0e9dd2198216184823e65d/1?pq-origsite=gscholar&cbl=40569

36. Suding KN, Hobbs RJ. Threshold models in restoration and conservation: a developing framework. Vol. 24, Trends in Ecology and Evolution. 2009. p. 271–9.

37. Scheffer M, Carpenter SR. Catastrophic regime shifts in ecosystems: Linking theory to observation. Trends Ecol Evol. 2003;18(12):648–56.

38. Biggs R, Carpenter SR, Brock WA. Turning back from the brink: Detecting an impending regime shift in time to avert it. Proc Natl Acad Sci [Internet]. 2009;106(3):826–31. Available from: www.pnas.org/cgi/content/full/

39. Jiang J, Huang ZG, Seager TP, Lin W, Grebogi C, Hastings A, et al. Predicting tipping points in mutualistic networks through dimension reduction. Proc Natl Acad Sci U S A. 2018 Jan 23;115(4):E639–47.

40. Samhouri JF, Levin PS, Ainsworth CH. Identifying thresholds for ecosystem-based management. PLoS One. 2010 Jan 26;5(1):1–10.

41. U.S. Army Corps of Engineers. Upper Mississippi River Restoration Program [Internet]. 2022 [cited 2022 Aug 29]. Available from: https://www.mvr.usace.army.mil/Missions/Environmental-Stewardship/Upper-Mississippi-River-Restoration/

42. De Jager NR, Rogala JT, Rohweder JJ, Van Appledorn M, Bouska KL, Houser JN, et al. Indicators of Ecosystem Structure and Function for the Upper Mississippi River System. OFR20181143. 2018.

43. U.S. Environmental Protection Agency. Ambient Water Quality Criteria Recommendations:

Information Supporting the Development of State and Tribal Nutrient Criteria Lakes and Reservoirs in Nutrient Ecoregion IX. 2000;EPA 822-B-00-019(December):1–108.

44. Carpenter S, Brock W. Rising variance: a leading indicator of ecological transition. Ecol Lett. 2006;9:311–8.

45. Capon SJ, Lynch AJJ, Bond N, Chessman BC, Davis J, Davidson N, et al. Regime shifts, thresholds and multiple stable states in freshwater ecosystems; a critical appraisal of the evidence. Sci Total Environ. 2015 Nov 15;534:122–30.

46. Groffman PM, Baron JS, Blett T, Gold AJ, Goodman I, Gunderson LH, et al. Ecological thresholds: The key to successful environmental management or an important concept with no practical application? Ecosystems. 2006;9(1):1–13.

47. Soballe DM, Fischer JR. Long Term Resource Monitoring Program Procedures: Water Quality Monitoring. U.S. Army Corps of Engineers Technical Report 2004-T002-01; 2004. 1–134 p.

48. Singh G, Mémoli F, Carlsson G. Topological Methods for the Analysis of High Dimensional Data Sets and 3D Object Recognition. Eurographics Symposium on Point-Based Graphics. Eurographics Symposium on Point-Based Graphics; 2007.

49. Kamruzzaman M, Kalyanaraman A, Krishnamoorthy B, Hey S, Schnable PS. Hyppo-X: A Scalable Exploratory Framework for Analyzing Complex Phenomics Data. IEEE/ACM Trans Comput Biol Bioinforma. 2021 Jul 1;18(4):1535–48.

**FIGURES**

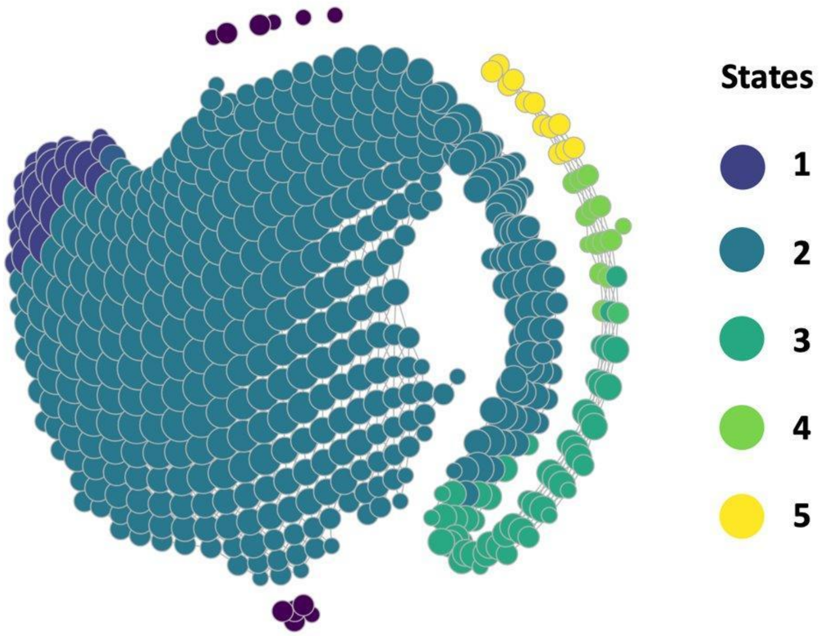


**Figure 1.**

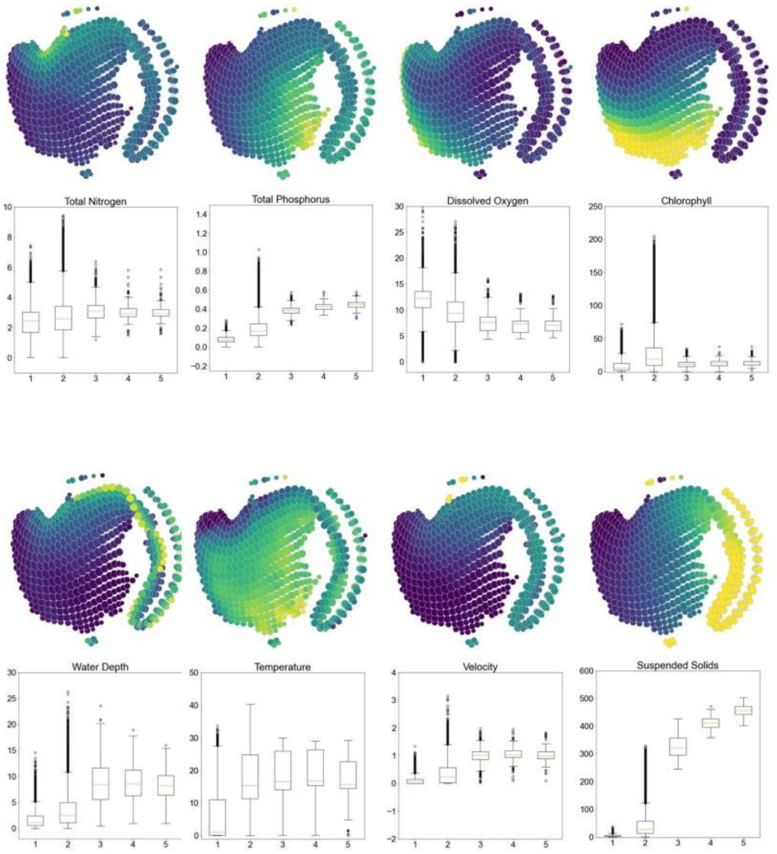
Total Nitrogen
Total Phosphorus
Dissolved Oxygen
Chlorophyll
Water Depth
Temperature
Velocity
Suspended Solids

**Figure 2.**

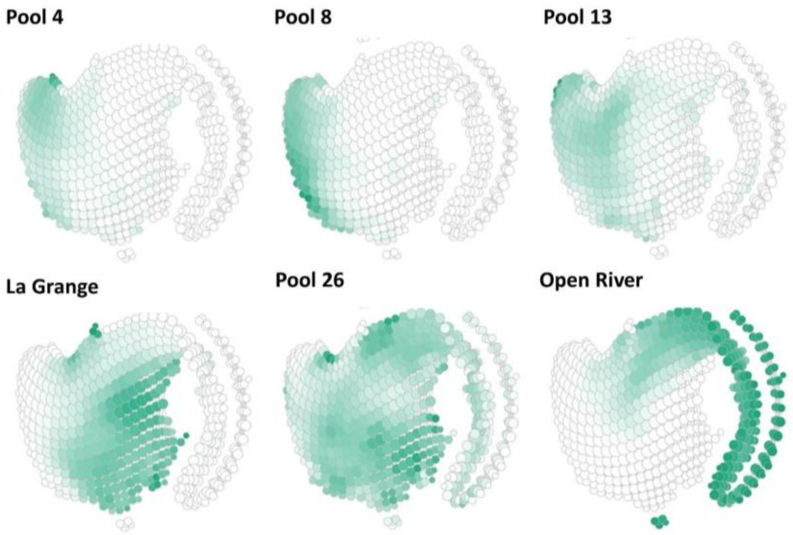


**Figure 3.**

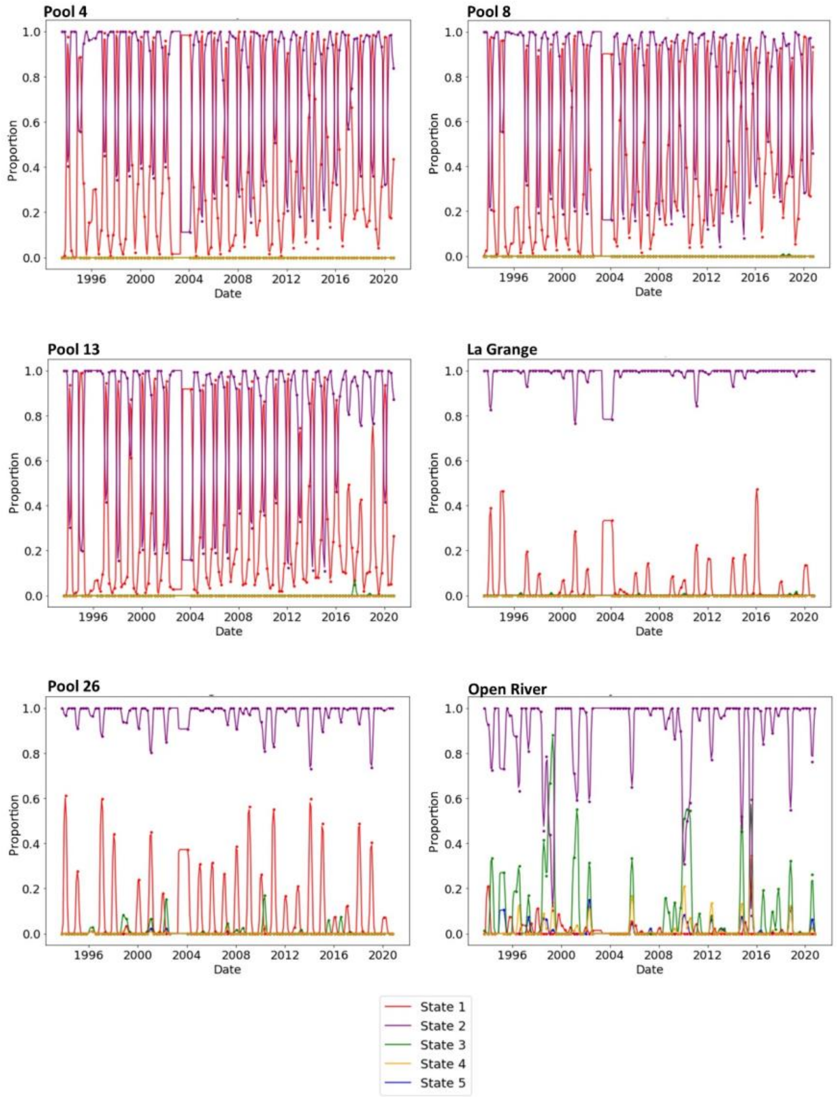
Pool 4
Pool 8
Pool 13
La Grange
Pool 26
Open River
Proportion
Date
State 1
State 2
State 3
State 4
State 5

**Figure 4.**

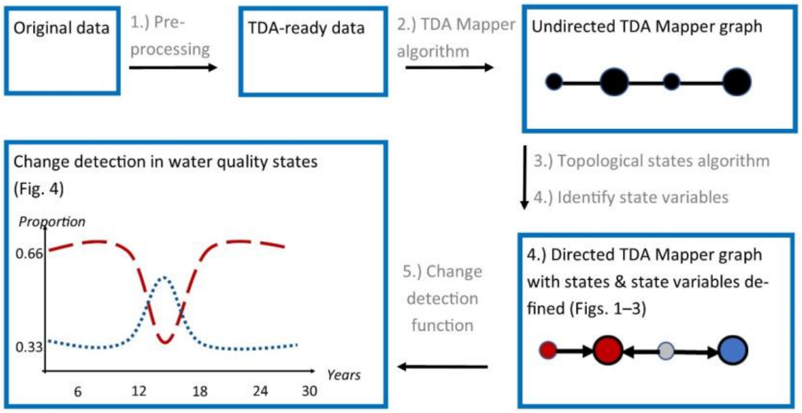
Original data
1.) Pre-processing
TDA-ready data
2.) TDA Mapper algorithm
Undirected TDA Mapper graph
3.) Topological states algorithm
4.) Identify state variables
4.) Directed TDA Mapper graph with states & state variables defined (Figs. 1–3)
5.) Change detection function
Change detection in water quality states (Fig. 4)
Proportion
0.66
0.33
Years
6
12
18
24
30

**Figure 5.**

## SUPPORTING INFORMATION

**S1 Figure**. Graphical output from topological data analysis (TDA Mapper) showing a strong seasonal component to the water quality data for the Upper Mississippi River System, USA. Data inputs included 8 water quality variables collected four times each year from 1993–2020 at a total of 69,307 sampling sites. The graph is comprised of nodes (circles), which cluster sampling sites along the river based on water quality similarities, and the edges (lines) show statistical connections among nodes. We highlighted in color the seasons in which sites were sampled. Seasonality can be connected to ecosystem states by referencing Figure 1 positions; for example, winter is typically characterized by State 1.

**Commented [LDM99]:** Added since revisions

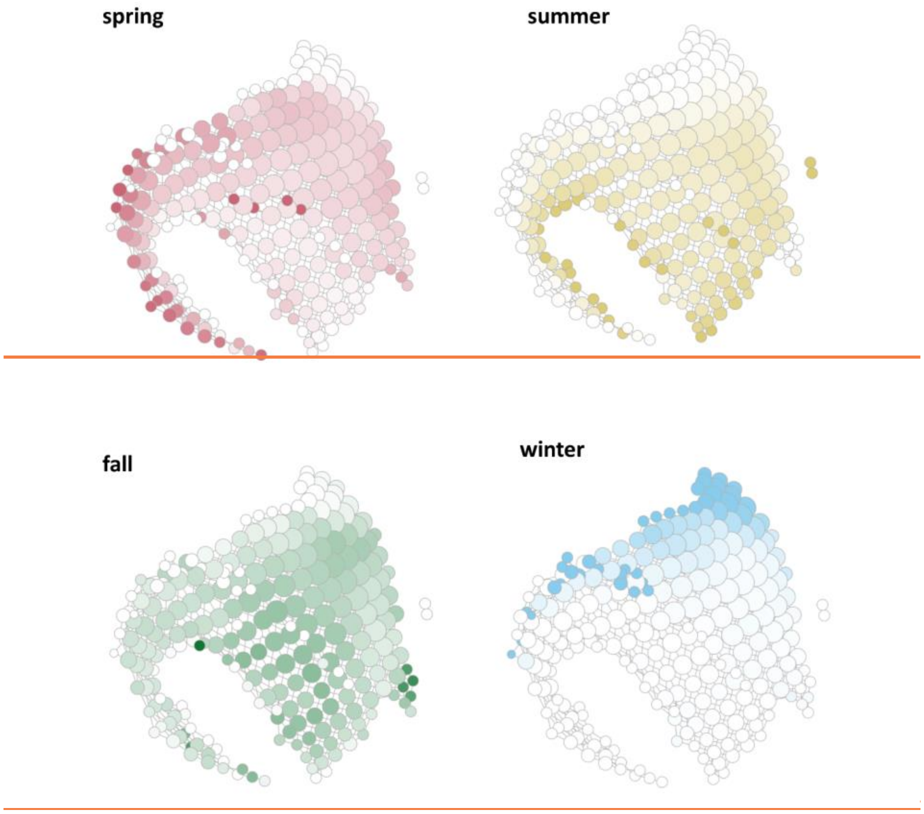
spring
summer
fall
winter

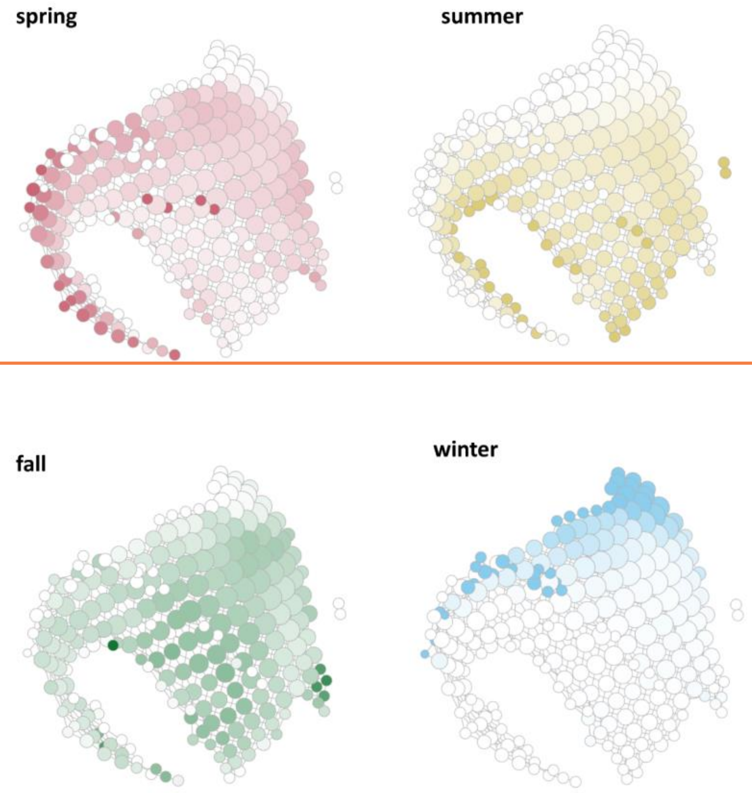


S1 Figure.
