# Supplementary material for: Quantifying ecosystem states and state transitions of the Upper Mississippi River System using topological data analysis
Source: PLoS Comput Biol. 2023 Jun 7;19(6):e1011147. doi: 10.1371/journal.pcbi.1011147 (PMC10246787; doi:10.1371/journal.pcbi.1011147)
Supplement: Attachment — Submitted filename: PCOMPBIOL-D-22-01462.docx [file pcbi.1011147.s002.docx]

This study focuses on analysis of a large-scale publicly-available dataset of water quality metrics collected from multiple sites across the Mississippi River over a span of 30 years. The authors’ goal is to synthesize the multi-dimensional data into meaningful classifications of water quality ‘states’. To do so, they apply topological data analysis (TDA) and identify 5 main ecosystem states, one of which they deem ‘healthy’, while the others were indicative of varying levels of ‘undesirable’ conditions. The authors propose 5 hypotheses relevant to this analysis, and briefly address each in the text. According to the authors, this is the first application of TDA to a river ecosystem, and they expect that this analysis will help those invested in the river’s health to better communicate river conditions and make decisions about restorative actions.

While the use of TDA to better understand the river system seems appropriate, this study fell short on a number of levels. 1) The methods were poorly described such that replicating the analysis would be impossible, 2) the interpretation of the results was often vague and not backed by statistics, 3) the analysis itself was very limited in scope, and 4) the discussion overstates the usefulness and generality of the findings.

1) The methods section states that data was downloaded from the usgs.gov website, and originally contained 204,345 rows (observations) and 133 columns (measured water quality variables). The description of how the data was filtered is very vague (p18. lines 369-374: *We filtered the data by stratified random sampling scheme, surface water, and 8 continuous variables. …)*, yet after filtering, the ‘*final data size was 69,307 rows by 8 columns’*. It is completely unclear how and why the 204k observations were filtered to only 69k, and they do provide an account of which sites were included in the final set. Furthermore, of the 133 variables, the authors ‘*chose eight water quality variables thought to be important for defining ecosystem states*’ (p17, ln 344-5). Why? Isn’t the point of TDA to find patterns in highly multidimensional data, and determine which suites of variables are important? The authors say they tested seven interpolation methods to address missingness but give no further details or results, nor do they provide any detail on how the one they chose (random forest algorithm) was implemented. The points above are major omissions in just the pre-processing section – the rest of the methods section was not any clearer.
2) TDA was performed and identified 5 states, but no confidence metrics were reported. The authors report that ‘total nitrogen was the only variable that didn’t vary across the five state classifications’ (line 171) but no statistics are used to validate that assertion, which they used to support one of their hypotheses. Similarly no statistics we provided to support the claims that ‘State 1 dominated in winter most of the years’ (line 222) or that ‘the increased minimum correlation values over time in State 1 was less pronounced than in Pools 4 and 8’ (line 227-8). These are just a few examples of many - overall no formal downstream analysis was performed beyond obtaining results from the TDA mapper algorithm.
3) The TDA results could have been used in a number of ways to provide insight into key markers of turning points in ecosystem health, and an assessment of the accuracy of these markers for future predictions or other systems. Instead, the authors state that: ‘*the states could become a categorical response variable in future models that aim to assess the environmental drivers and threshold responses’* (line 315) yet do not perform this analysis themselves, and ‘*The TDA also allows extraction of information at finer spatiotemporal scales but was beyond the scope of this paper’* (line 276-7). The authors did not remark on whether these same states might apply to other river systems or do any analysis of whether there were meaningful substates within the 5 main states.
4) The authors claim these results will be useful for making decisions about restorative actions for the Mississippi river, but do not give any clear examples of how one would go about this. Their discussion section on transferability of TDA to other ecosystems says that *‘TDA may provide an early warning indication of vulnerability’*, but do not say what those warnings might be in this system, or whether the same variables would be a warning in other systems. If the whole analysis must be performed from scratch to gain insight into other systems, it is unclear whether the methodology is in fact generalizable; since this dataset is exceptional in terms of scope and depth of data collection, it seems unlikely that this same analysis would succeed in other less well-sampled systems. The last sentence of the paper is that *‘state concepts and TDA methodologies can be transferred to any ecosystem … ‘* but that was true regardless of the work presented here. Overall it is unclear whether this work offers a significant contribution.

Other points:

- The phrase ‘state variable’ was used many times, but the definition was never given, nor were criteria defined for
- Why were three state variables hypothesized (Line 109) ? What was this number based on? Why were 8 variables chosen for the analysis?
- The methods should be summarized succinctly in the main text so the description of results has context. As is, the first sentence of the results section is ‘*The TDA Mapper graph output has six connected components…*’ (line 118), but neither the TDA Mapper tool itself nor its expected output has been described. What do nodes and components represent?
- Line 125 (and elsewhere) states that there were 69,307 sites, while line 130 states that there were 76,669 sites – which is correct?
- The caption for Table 1 is not sufficient to clearly explain the difference between the two tables (1a and 1b) – this is made more confusing by the fact that ‘%’ signs are included in 1a but not 1b, while both are supposed to be proportions.
- Captions for figures 3 and 4 should contain description of the plots, not interpretation – that belongs in the main text
- Line 310: *The TDA tool indicated Pool 13 may be vulnerable to transitioning to the undesirable turbid state (State 3; Figure 4) because it has transitioned to State 3 repeatedly only in the last few years of a 30-year record*… So, transitioning to state 3 is an early warning sign of transitioning to state 3?
- Line 451-453: *We did not run formal statistical analysis for ‘statistical significance’ among the five boxplots (e.g., analysis of variance among the states) because the high number of data points (n=76,669) and degrees of freedom precluded that option.*  It does not make sense to me why statistical tests were not used to compare the distributions; a large number of observations should not be a problem. Furthermore, *n* is reported elsewhere as 69,307 – where did the number reported here come from?
- Lines 455-464: The authors define a ‘temporal correlation function’ as simply the proportion of all measurements that are assigned to a given state. I do not see how this can be called a ‘correlation’ – yet this is exactly what is plotted in Figure 4, and the y-axis is labeled ‘correlation’.
