# Supplementary material for: Quantifying ecosystem states and state transitions of the Upper Mississippi River System using topological data analysis
Source: PLoS Comput Biol. 2023 Jun 7;19(6):e1011147. doi: 10.1371/journal.pcbi.1011147 (PMC10246787; doi:10.1371/journal.pcbi.1011147)
Supplement: Attachment — Submitted filename: PLOSCompBio Response to Reviewers April 2023.docx [file pcbi.1011147.s006.docx]

PCOMPBIOL-D-22-01462R1

Response to reviews (in blue text):

Reviewer #1: This is an excellent applied manuscript on TDA and a new inspiration to the TDA community. Thank you so much for a thorough review and support!

Reviewer #2: The additions and edits the authors made have improved the clarity of the methods and motivations, and the additional discussion has better placed this study in context with respect to its relevance for other rivers. I would still suggest the authors proofread carefully! But otherwise I think the study stands as a nice application to an important dataset of an analytic method that is useful for synthesizing large-scale data into meaningful and actionable conclusions. We fully appreciate the reviewers’ comments. Authors have carefully revised and proof-read the manuscript with tracked changes. Thanks again.

Reviewer #3: The authors have substantially revised their previously submitted manuscript. Most of my previous comments have been satisfactorily addressed. My remaining comments are provided using the adobe comments tool in the attached pdf. Thank you so much. We responded to each comment within the pdf and reference the line numbers that were changed in the Word document. We also tracked all changes in the revised Word document.
